# Supplementary material for: Exploring the values and preferences of children and adolescents with obesity and their parents/caregivers concerning diet or physical activity interventions for weight management: Mega-ethnography of qualitative syntheses
Source: PLoS One. 2026 Jan 20;21(1):e0340875. doi: 10.1371/journal.pone.0340875 (PMC12818672; doi:10.1371/journal.pone.0340875)
Supplement: S1 Appendix — (DOCX) [file pone.0340875.s001.docx]

## **S1 Appendix. Search strategy for reviews 1-5 [Ovid MEDLINE]**

23/01/2022 Search history sorted by search number descending

18 limit 17 to yr="2010 - 2021" 182

17 15 and 16 190

16 (Child or children or pediatr$ or paediatric$ or Child, Preschool or Adolescent$ or Adolescence or Youth or Youths or parent or parents or mother or mothers or father or fathers or relative or relatives or caregiver$ or Minors or minors$ or boy or boys or boyhood or girl$ or kid or kids or schoolchild$ or schoolchild or school child$ or juvenil$ or youth$ or teen$ or under$age$ or pubescen$).mp. [mp=title, abstract, original title, name of substance word, subject heading word, floating sub-heading word, keyword heading word, organism supplementary concept word, protocol supplementary concept word, rare disease supplementary concept word, unique identifier, synonyms] 4745369

15 13 and 14 433

14 (obesity or obese or obesogenic).mp. [mp=title, abstract, original title, name of substance word, subject heading word, floating sub-heading word, keyword heading word, organism supplementary concept word, protocol supplementary concept word, rare disease supplementary concept word, unique identifier, synonyms] 363377

13 1 or 2 or 3 or 4 or 5 or 6 or 7 or 8 or 9 or 10 or 11 or 12 18685

12 (critical interpretive synthesis or (qualitative adj4 systematic$) or (qualitative adj4 review) or (qualitative adj4 synthes$)).mp. [mp=title, abstract, original title, name of substance word, subject heading word, floating sub-heading word, keyword heading word, organism supplementary concept word, protocol supplementary concept word, rare disease supplementary concept word, unique identifier, synonyms] 8756

11 (meta-study or metastudy or meta study or meta synthese or meta syntheses or meta synthesis or metasynthes$ or meta-synthes$ or meta synthesise or meta synthesised or meta synthesist or meta synthesized or meta synthesizing or metasummar$ or meta-summar$ or meta summary or metanarrative$ or meta-narrative$ or meta narrative$).mp. [mp=title, abstract, original title, name of substance word, subject heading word, floating sub-heading word, keyword heading word, organism supplementary concept word, protocol supplementary concept word, rare disease supplementary concept word, unique identifier, synonyms] 1754

10 ((synthesis and ("qualitative literature" or "qualitative research")) or ("systematic review" and ("qualitative research" or "qualitative literature" or "qualitative studies"))).mp. [mp=title, abstract, original title, name of substance word, subject heading word, floating sub-heading word, keyword heading word, organism supplementary concept word, protocol supplementary concept word, rare disease supplementary concept word, unique identifier, synonyms] 4570

9 ((("quality assessment" or "critical appraisal" or checklist$) and ("mixed method" or "mixed methods" or "mixed studies" or "mixed study" or "mixed research")) or ("Mixed Methods Appraisal Tool" or MMAT)).mp. [mp=title, abstract, original title, name of substance word, subject heading word, floating sub-heading word, keyword heading word, organism supplementary concept word, protocol supplementary concept word, rare disease supplementary concept word, unique identifier, synonyms] 1041

8 (("literature search" or "literature searching" or "literature searches") and ("mixed method" or "mixed methods" or "mixed studies" or "mixed study" or "mixed research")).mp. [mp=title, abstract, original title, name of substance word, subject heading word, floating sub-heading word, keyword heading word, organism supplementary concept word, protocol supplementary concept word, rare disease supplementary concept word, unique identifier, synonyms] 213

7 ((("systematic review" or "systematic reviews") and ("mixed method" or "mixed methods" or "mixed studies" or "mixed study" or "mixed research")) or ((synthesis or syntheses) and ("mixed method" or "mixed methods" or "mixed studies" or "mixed study" or "mixed research"))).mp. [mp=title, abstract, original title, name of substance word, subject heading word, floating sub-heading word, keyword heading word, organism supplementary concept word, protocol supplementary concept word, rare disease supplementary concept word, unique identifier, synonyms] 2147

6 ((Noblit and Hare) or (CERQUAL or CONQUAL) or (JBI-QARI or QualSys)).mp. [mp=title, abstract, original title, name of substance word, subject heading word, floating sub-heading word, keyword heading word, organism supplementary concept word, protocol supplementary concept word, rare disease supplementary concept word, unique identifier, synonyms] 325

5 ((("quality assessment" or "critical appraisal" or checklist$) and ("qualitative literature" or "qualitative research" or "qualitative paper" or "qualitative papers" or "qualitative studies" or qualitative study or realist)) or (synthesis and ("qualitative literature" or "qualitative research")) or ("systematic review" and ("qualitative research" or "qualitative literature" or "qualitative studies"))).mp. [mp=title, abstract, original title, name of substance word, subject heading word, floating sub-heading word, keyword heading word, organism supplementary concept word, protocol supplementary concept word, rare disease supplementary concept word, unique identifier, synonyms] 5517

4 (("literature search" or "literature searching" or "literature searches") and ("qualitative literature" or "qualitative research" or "qualitative paper" or "qualitative papers" or "qualitative studies" or qualitative study or realist)).mp. [mp=title, abstract, original title, name of substance word, subject heading word, floating sub-heading word, keyword heading word, organism supplementary concept word, protocol supplementary concept word, rare disease supplementary concept word, unique identifier, synonyms] 676

3 (meta-triangulation or meta triangulation or meta triangulation or realist review or realist reviews or realist synthesis or realist syntheses or thematic synthesis or thematic syntheses or ((synthesis or syntheses) and Thematic analysis) or ((systematic review or systematic reviews) and (Thematic analysis or framework synthesis or framework syntheses))).mp. [mp=title, abstract, original title, name of substance word, subject heading word, floating sub-heading word, keyword heading word, organism supplementary concept word, protocol supplementary concept word, rare disease supplementary concept word, unique identifier, synonyms] 2475

2 ((("integrative synthesis" or "integrative syntheses") and qualitative) or (("integrative review" or "integrative reviews") and qualitative) or ("interpretive synthesis" or "interpretive syntheses") or (Mega-ethnograph$ or megaethnograph$ or "mega ethnograph$" or (meta-ethnog$ or metaethnog$ or "meta ethnograph$") or ("meta interpretation" or "meta interpretive") or meta interpretation or meta interpretive or (Meta-method$ or "meta method$" or metamethod$) or "narrative synthesis" or "narrative syntheses")).mp. [mp=title, abstract, original title, name of substance word, subject heading word, floating sub-heading word, keyword heading word, organism supplementary concept word, protocol supplementary concept word, rare disease supplementary concept word, unique identifier, synonyms] 4656

1 ("Qualitative systematic review" or "qualitative systematic reviews" or "qualitative evidence synthesis" or "qualitative evidence syntheses" or "qualitative research synthesis" or "qualitative research syntheses" or "Qualitative synthesis" or "qualitative syntheses").mp. [mp=title, abstract, original title, name of substance word, subject heading word, floating sub-heading word, keyword heading word, organism supplementary concept word, protocol supplementary concept word, rare disease supplementary concept word, unique identifier, synonyms] 2750
