# Supplementary material for: Exploring the values and preferences of children and adolescents with obesity and their parents/caregivers concerning diet or physical activity interventions for weight management: Mega-ethnography of qualitative syntheses
Source: PLoS One. 2026 Jan 20;21(1):e0340875. doi: 10.1371/journal.pone.0340875 (PMC12818672; doi:10.1371/journal.pone.0340875)
Supplement: S2 Appendix — (DOCX) [file pone.0340875.s002.docx]

### **S2 Appendix.** **Review author reflexivity**

The author team represents diverse professional backgrounds with varied research experiences and expertise that could have biased their input in conducting this review. All of them are experienced systematic reviewers, including reviews of reviews (AB, CC, ME) and qualitative evidence synthesis (AB, CC, JL). Two of the review team has also previously conducted reviews of reviews for the same funder on the topics of obesity in children and adolescents, infant feeding and ready-to-use therapeutic foods in children (AB, CC). These reviews included positive, negative, and mixed results. This provided us with a good platform for engaging and understanding the complexities and nuances of qualitative research in this age group.

During the screening, at abstracts / titles and full text stages, the team constantly referred to each other to resolve conflicts, which was an effective counter measure to any individual bias they may have had. Many instances called for a team decision was called upon, which further countered our biases. As is standard practice within qualitative research, the review authors [CC, AB, ME, JL, DC] who did the data coding, extraction, and synthesising, and wrote the findings, constantly reflected on how their own background and position, may have affected their analysis and writing of the findings. To minimise their biases, reviewers questioned each other’s interpretation of the data and how it fitted with the interpreted findings. They also called upon other members of the review team to verify that the findings were true reflections of the supporting data. The same process of constant discussion and being aware of their personal biases, applied during the appraisal of confidence in the findings which was done by CC and AB. A final measure to counter possible biases in the review findings, was consultations with the contact editors of this review (JP, HP).
