# Supplementary material for: Exploring the values and preferences of children and adolescents with obesity and their parents/caregivers concerning diet or physical activity interventions for weight management: Mega-ethnography of qualitative syntheses
Source: PLoS One. 2026 Jan 20;21(1):e0340875. doi: 10.1371/journal.pone.0340875 (PMC12818672; doi:10.1371/journal.pone.0340875)
Supplement: S1 Table — (DOCX) [file pone.0340875.s004.docx]

**Table S1. Reviews excluded with reasons**

Reviews excluded when checking full-text articles (identified from various sources) with main reason for exclusion as of 6^th^ June 2022 and July 2024.

|  | Abdin, S., Heath, G., & Welch, R. K. (2021). Health professionals' views and experiences of discussing weight with children and their families: A systematic review of qualitative research. Child: Care, Health and Development, 47(4), 562-574. | Excluded - only Health Professionals' views |
| --- | --- | --- |
|  | Aboueid, S., Ahmed, R., Jasinska, M., Pouliot, C., Hermosura, B. J., Bourgeault, I., & Giroux, I. (2020). Weight Communication: How Do Health Professionals Communicate about Weight with Their Patients in Primary Care Settings?. Health Communication, 1-7. | Excluded - Not Adolescents |
|  | Aceves-Martins, M., López-Cruz, L., García-Botello, M., Gutierrez-Gómez, Y. Y., & Moreno-García, C. F. (2021). Interventions to Prevent Obesity in Mexican Children and Adolescents: Systematic Review. Prevention Science, 1-24. | Excluded - Not Treatment |
|  | Adamo, K. B., & Brett, K. E. (2014). Parental perceptions and childhood dietary quality. Maternal and child health journal, 18(4), 978-995. | Excluded - No Interventions |
|  | Adom T, De Villiers A, Puoane T, Kengne AP. School-Based Interventions Targeting Nutrition and Physical Activity, and Body Weight Status of African Children: A Systematic Review. Nutrients. 2019 Dec 30;12(1):95. doi: 10.3390/nu12010095. PMID: 31905832; PMCID: PMC7019429. | Excluded - Not Qualitative |
|  | Alman KL, Lister NB, Garnett SP, Gow ML, Aldwell K, Jebeile H. Dietetic management of obesity and severe obesity in children and adolescents: A scoping review of guidelines. Obesity Reviews [Internet]. 2020 Sep 7;22(1). Available from: <http://dx.doi.org/10.1111/obr.13132> | Excluded - Not Qualitative |
|  | Alsubhi, M., et al. (2020). What factors are associated with obesity-related health behaviours among child refugees following resettlement in developed countries? A systematic review and synthesis of qualitative and quantitative evidence. Obesity Reviews 21(11): e13058. | Excluded – No Interventions |
|  | Alulis, S., & Grabowski, D. (2017). Theoretical frameworks informing family-based child and adolescent obesity interventions: A qualitative meta-synthesis. Obesity Research & Clinical Practice, 11(6), 627-639. | Excluded – Focus on Theory not Intervention |
|  | Ames, H., et al. (2020). "Communication of children's weight status: what is effective and what are the children's and parents' experiences and preferences? A mixed methods systematic review." BMC Public Health 20(1): 574. | Excluded from Review 3 (no exercise data) |
|  | Arai, L., Panca, M., Morris, S., Curtis-Tyler, K., Lucas, P. J., & Roberts, H. M. (2015). Time, monetary and other costs of participation in family-based child weight management interventions: qualitative and systematic review evidence. PLoS One, 10(4), e0123782. | Excluded – Not Qualitative (Includes stand-alone primary qualitative study) |
|  | Archibald, D., Douglas, F., Hoddinott, P., Van Teijlingen, E., Stewart, F., Robertson, C., ... & Avenell, A. (2015). A qualitative evidence synthesis on the management of male obesity. BMJ open, 5(10), e008372. | Excluded - Not Adolescents |
|  | Bagnall, A. M., et al. (2019). Whole systems approaches to obesity and other complex public health challenges: a systematic review. BMC Public Health 19(1): 8 | Excluded – No Interventions |
|  | Banna J, Bersamin A. Community involvement in design, implementation and evaluation of nutrition interventions to reduce chronic diseases in indigenous populations in the U.S.: a systematic review. Int J Equity Health. 2018 Aug 13;17(1):116. doi: 10.1186/s12939-018-0829-6. PMID: 30103753; PMCID: PMC6090789. | Excluded - Not Adolescents |
|  | Baranowski T, O'Connor T, Johnston C, Hughes S, Moreno J, Chen TA, Meltzer L, Baranowski J. School year versus summer differences in child weight gain: a narrative review. Childhood Obesity. 2014 Feb 1;10(1):18-24. | Excluded - Not Interventions |
|  | Bean MK, Caccavale LJ, Adams EL, Burnette CB, LaRose JG, Raynor HA, Wickham EP 3rd, Mazzeo SE. Parent Involvement in Adolescent Obesity Treatment: A Systematic Review. Pediatrics. 2020 Sep;146(3):e20193315. doi: 10.1542/peds.2019-3315. | Excluded RCTs only |
|  | Bennett L, Burns S. Implementing health-promoting schools to prevent obesity. Health Education. 2020 Jun 8;120(2):197-216. | Excluded – Health Promotion |
|  | Besson M, Gurviez P, Carins J. Using digital devices to help people lose weight: a systematic review. Journal of social marketing. 2020 Jul 30. | Excluded - Not Qualitative |
|  | Bianco, A., Jemni, M., Thomas, E., Patti, A., Paoli, A., Ramos Roque, J., Palma, A., Mammina, C. and Tabacchi, G., 2015. A systematic review to determine reliability and usefulness of the field-based test batteries for the assessment of physical fitness in adolescents—The ASSO Project. Int J Occup Med Environ Health, 28(3), pp.445-478. | Excluded - Not Qualitative |
|  | Biddle SJ, Petrolini I, Pearson N. Interventions designed to reduce sedentary behaviours in young people: a review of reviews. Br J Sports Med. 2014 Feb;48(3):182-6. doi: 10.1136/bjsports-2013-093078. Epub 2013 Dec 17. PMID: 24347578. | Excluded - Not Qualitative |
|  | Blower S, Swallow V, Maturana C, Stones S, Phillips R, Dimitri P, Marshman Z, Knapp P, Dean A, Higgins S, Kellar I, Curtis P, Mills N, Martin-Kerry J. Children and young people's concerns and needs relating to their use of health technology to self-manage long-term conditions: a scoping review. Arch Dis Child. 2020 Nov;105(11):1093-1104. doi: 10.1136/archdischild-2020-319103. Epub 2020 May 22. PMID: 32444448; PMCID: PMC7588410. | Excluded - Not Obesity |
|  | Boone, K, "Overcoming Improving Providers’ Attitude in Addressing Obesity in Patients.", Georgia State University, 2020. <https://scholarworks.gsu.edu/nursing_dnpprojects/25> | Excluded, only Health Professionals' views |
|  | Bradbury D, Chisholm A, Watson PM, Bundy C, Bradbury N, Birtwistle S. Barriers and facilitators to health care professionals discussing child weight with parents: A meta‐synthesis of qualitative studies. British Journal of Health Psychology. 2018 Sep;23(3):701-22. | Excluded, only Health Professionals' views |
|  | Braden, K. W. and C. R. Nigg (2016). Modifiable Determinants of Obesity in Native Hawaiian and Pacific Islander Youth. Hawai'i Journal of Medicine & Public Health : A Journal of Asia Pacific Medicine & Public Health 75(6): 162-171. | Excluded – No Interventions |
|  | Bristow C, Meurer C, Simmonds J, Snell T. Anti-obesity public health messages and risk factors for disordered eating: a systematic review. Health promotion international. 2020 Dec;35(6):1551-69. | Excluded - No Interventions |
|  | Bungay, H. and T. Vella-Burrows (2013). The effects of participating in creative activities on the health and well-being of children and young people: a rapid review of the literature. Perspectives in Public Health 133(1): 44-52. | Excluded – Not Included Intervention |
|  | Buru K, Emeto TI, Malau-Aduli AE, Malau-Aduli BS. The efficacy of school-based interventions in preventing adolescent obesity in Australia. In Healthcare 2020 Dec (Vol. 8, No. 4, p. 514). Multidisciplinary Digital Publishing Institute. | Excluded - Not Qualitative |
|  | Castronuovo, L., Guarnieri, L., Tiscornia, V., & Allemandi, L. (2021). Food Marketing, Eating Behaviors and Gender Among Children and Adolescents: A Scoping Review. | Excluded - No Interventions |
|  | Caughey, A. B., Sargeant, J. M., Møller, H., & Harper, S. L. (2021). Inuit country food and health during pregnancy and early childhood in the circumpolar north: a scoping review. International journal of environmental research and public health, 18(5), 2625. | Excluded - No Interventions |
|  | Chatham, R. E. and S. J. Mixer (2020). Cultural Influences on Childhood Obesity in Ethnic Minorities: A Qualitative Systematic Review. Journal of Transcultural Nursing 31(1): 87-99. | Excluded – No Interventions |
|  | Chatterjee A, Prinz A, Gerdes M, Martinez S. Digital Interventions on Healthy Lifestyle Management: Systematic Review. Journal of Medical Internet Research. 2021 Nov 17;23(11):e26931. | Excluded – Not Adolescents |
|  | Rosales Chavez JB, Garcia LM, Jehn M, Pereira MA, Bruening M. Relationship between different levels of the Mexican food environment and dietary intake: a qualitative systematic review. Public Health Nutr. 2020 Aug;23(11):1877-1888. doi: 10.1017/S1368980019004294. Epub 2020 Mar 27. PMID: 32216850. | Excluded – No Interventions |
|  | Chavez-Ugalde, Yanaina et al. “Conceptualizing the commercial determinants of dietary behaviors associated with obesity: A systematic review using principles from critical interpretative synthesis.” Obesity science & practice vol. 7,4 473-486. 5 Apr. 2021, doi:10.1002/osp4.507 | Excluded – No Interventions |
|  | Chriqui JF, Pickel M, Story M. Influence of school competitive food and beverage policies on obesity, consumption, and availability: a systematic review. JAMA pediatrics. 2014 Mar 1;168(3):279-86. | Excluded - Not Qualitative |
|  | Clarke, J., Fletcher, B., Lancashire, E., Pallan, M., & Adab, P. (2013). The views of stakeholders on the role of the primary school in preventing childhood obesity: a qualitative systematic review. Obesity Reviews, 14(12), 975-988. | Excluded – Health Promotion |
|  | Clarke, J. L. (2016). The role of the primary school in preventing childhood obesity (Doctoral dissertation, University of Birmingham). | Excluded - Not Qualitative |
|  | Confiac, N., et al. (2020). "Mexican American Parental Knowledge and Perceptions of Childhood Obesity: An Integrative Review." Hispanic Health Care International : The Official Journal of The National Association of Hispanic Nurses 18(2): 105-116. | Excluded – No Interventions |
|  | Coulman KD, MacKichan F, Blazeby JM, Owen‐Smith A. Patient experiences of outcomes of bariatric surgery: a systematic review and qualitative synthesis. Obesity reviews. 2017 May;18(5):547-59. | Excluded - Not Adolescents |
|  | Cui Z, Seburg EM, Sherwood NE, Faith MS, Ward DS. Recruitment and retention in obesity prevention and treatment trials targeting minority or low-income children: a review of the clinical trials registration database. Trials. 2015 Dec;16(1):1-5. | Excluded - Not Qualitative |
|  | Curtis P, Thompson J, Fairbrother H. Migrant children within Europe: a systematic review of children's perspectives on their health experiences. Public Health. 2018 May;158:71-85. doi: 10.1016/j.puhe.2018.01.038. Epub 2018 Apr 5. PMID: 29627115. | Excluded - Not Obesity |
|  | Dankiw KA, Tsiros MD, Baldock KL, Kumar S. The impacts of unstructured nature play on health in early childhood development: A systematic review. Plos one. 2020 Feb 13;15(2):e0229006. | Excluded - Not Interventions |
|  | Dattilo AM, Carvalho RS, Feferbaum R, Forsyth S, Zhao A. Hidden realities of infant feeding: Systematic review of qualitative findings from parents. Behavioral Sciences. 2020 May;10(5):83. | Excluded - Not Interventions |
|  | Demers C, Brochu A, Higgins J, Gélinas I. Complex behavioral interventions targeting physical activity and dietary behaviors in pediatric oncology: A scoping review. Pediatric Blood & Cancer. 2021 Aug;68(8):e29090. | Excluded - Not Qualitative |
|  | Deyra M, Gay C, Gerbaud L, Berland P, Pizon F. Global health determinants perceived and expressed by children and adolescents between 6 and 17 years: a systematic review of qualitative studies. Frontiers in Pediatrics. 2020 Apr 3;8:115. | Excluded - Not Obesity |
|  | Dhaliwal, J., et al. (2014). "Attrition and the management of pediatric obesity: an integrative review." Childhood Obesity 10(6): 461-473. | Excluded - Focus not on Intervention but trial. |
|  | Diep CS, Foster MJ, McKyer EL, Goodson P, Guidry JJ, Liew J. What are Asian-American youth consuming? A systematic literature review. Journal of Immigrant and Minority Health. 2015 Apr;17(2):591-604. | Excluded - Not Interventions |
|  | Dixon BN, Ugwoaba UA, Brockmann AN, Ross KM. Associations between the built environment and dietary intake, physical activity, and obesity: A scoping review of reviews. Obesity Reviews. 2021 Apr;22(4):e13171. | Excluded - Not Interventions |
|  | Dixon, M. O. (2012). "Elementary school personnel's perceptions of and recommendations for managing child obesity: A naturalistic study." Dissertation Abstracts International Section A: Humanities and Social Sciences 73(3-A): 892. | Exclude – Not Child or Parental Perspectives |
|  | Enright, G., et al. (2020). "Effectiveness of Family-Based Behavior Change Interventions on Obesity-Related Behavior Change in Children: A Realist Synthesis." International Journal of Environmental Research & Public Health [Electronic Resource] 17(11): 08. | Excluded from Review 3 (no exercise data) |
|  | Farnesi, B. C., et al. (2012). "Family-health professional relations in pediatric weight management: an integrative review." Pediatric Obesity 7(3): 175-186. | Excluded from Review 3 (no exercise data) |
|  | Fraser, J., et al. (2011). Paternal Influences on Children's Weight Gain: A Systematic Review. Fathering: A Journal of Theory, Research, and Practice about Men as Fathers 9(3): 252-267. | Excluded – No Interventions |
|  | Grootens-Wiegers, P., et al. (2020). "The “Stages Towards Completion Model”: what helps and hinders children with overweight or obesity and their parents to be guided towards, adhere to and complete a group lifestyle intervention." International Journal of Qualitative Studies on Health and Well-being 15(1). | Excluded from Review 3 (no exercise data) |
|  | Harrison M, Brodribb W, Hepworth J. A qualitative systematic review of maternal infant feeding practices in transitioning from milk feeds to family foods. Maternal & child nutrition. 2017 Apr;13(2):e12360. | Excluded – No Interventions |
|  | Hesketh, K. R., Lakshman, R., & van Sluijs, E. M. (2017). Barriers and facilitators to young children's physical activity and sedentary behaviour: a systematic review and synthesis of qualitative literature. Obesity Reviews, 18(9), 987-1017. | Excluded – No Interventions |
|  | Hnatiuk JA, Brown HE, Downing KL, Hinkley T, Salmon J, Hesketh KD. Interventions to increase physical activity in children 0–5 years old: a systematic review, meta‐analysis and realist synthesis. Obesity Reviews. 2019 Jan;20(1):75-87. | Excluded - Not Qualitative |
|  | Hoare JK, Jebeile H, Garnett SP, Lister NB. Novel dietary interventions for adolescents with obesity: A narrative review. Pediatric Obesity. 2021 May 5;16(9). Available from: <http://dx.doi.org/10.1111/ijpo.12798> | Excluded - Not Qualitative |
|  | Ibáñez, C. P. B., & Heredia, L. P. D. (2019). Características de las intervenciones realizadas con adolescentes en condición de sobrepeso y obesidad: una revisión integrativa. Revista Cuidarte, 10(3). | Excluded - Not Qualitative |
|  | Jebeile H, Lister NB, Baur LA, Garnett SP, Paxton SJ. Eating disorder risk in adolescents with obesity. Obesity Reviews. 2021 Jan 6;22(5). Available from: <http://dx.doi.org/10.1111/obr.13173> | Excluded - Not Qualitative |
|  | Kairey, L.1; Matvienko-Sikar, K.1; Kelly, C.2; McKinley, M. C.; O’Connor, E. M.4; Kearney, P. M.1; Woodside, J. V.3; Harrington, J. M.1- Supplement . T3P36 - Portion size in parents’ eyes: a mixed methods systematic review of parental portioning practices for their children. | Excluded – No Interventions |
|  | Kanji, S., Wong, E., Akioyamen, L., Melamed, O., & Taylor, V. H. (2019). Exploring pre-surgery and post-surgery substance use disorder and alcohol use disorder in bariatric surgery: a qualitative scoping review. International Journal of Obesity, 43(9), 1659-1674. | Excluded – Not Adolescents |
|  | Keyworth C, Epton T, Goldthorpe J, Calam R, Armitage CJ. Delivering opportunistic behavior change interventions: A systematic review of systematic reviews. Prevention Science. 2020 Apr;21(3):319-31. | Excluded – Not Adolescents |
|  | Klingberg, S., Draper, C. E., Micklesfield, L. K., Benjamin-Neelon, S. E., & van Sluijs, E. M. (2019). Childhood obesity prevention in Africa: a systematic review of intervention effectiveness and implementation. International journal of environmental research and public health, 16(7), 1212. | Excluded – Not Adolescent Perspectives |
|  | Klingberg, S. (2020). Childhood obesity prevention in Soweto, South Africa (Doctoral dissertation, University of Cambridge). | Excluded – Not Adolescent Perspectives |
|  | Kobes A, Kretschmer T, Timmerman G, Schreuder P. Interventions aimed at preventing and reducing overweight/obesity among children and adolescents: a meta‐synthesis. Obesity reviews. 2018 Aug;19(8):1065-79. | Excluded – Not Qualitative |
|  | König, L. M., Attig, C., Franke, T., & Renner, B. (2021). Barriers to and facilitators for using nutrition apps: systematic review and conceptual framework. JMIR mHealth and uHealth, 9(6), e20037. | Excluded – Not Adolescents |
|  | Kucharczuk AJ, Oliver TL, Dowdell EB. Social media’s influence on adolescents′ food choices: A mixed studies systematic literature review. Appetite. 2022 Jan 1;168:105765. | Excluded – No Interventions |
|  | Lakshman, R., Griffin, S., Hardeman, W., Schiff, A., Kinmonth, A. L., & Ong, K. K. (2014). Using the Medical Research Council framework for the development and evaluation of complex interventions in a theory-based infant feeding intervention to prevent childhood obesity: the baby milk intervention and trial. *Journal of obesity*, 2014. | Excluded – No Interventions |
|  | Lampe, E. W., Abber, S. R., Forman, E. M., & Manasse, S. M. (2020). Guidelines for caregivers and healthcare professionals on speaking to children about overweight and obesity: A systematic review of the gray literature. Translational Behavioral Medicine, 10(5), 1144-1154. | Excluded – Not Qualitative |
|  | Lanigan J, Adegboye A, Northstone K, Salisbury C, Singhal A. Nutrition in preschool children and later risk of obesity: a systematic review and meta analysis. Journal of Pediatric Gastroenterology and Nutrition. 2016;62:691-2. | Excluded - Not Qualitative |
|  | Leandro, C. G., et al. (2019). "Barriers and Enablers That Influence Overweight/Obesity/Obesogenic Behavior in Adolescents From Lower-Middle Income Countries: A Systematic Review." Food & Nutrition Bulletin 40(4): 562-571. | Excluded – No Interventions |
|  | Leung, M. M., Cavalcanti, O. B., El Dada, A., Brown, M., Mateo, K. F., & Yeh, M. C. (2017). Treating obesity in Latino children: A systematic review of current interventions. International Journal of Child Health and Nutrition, 6(1), 1-15. | Excluded - Not Qualitative |
|  | Li, P. P., Mackey, G., Callender, C., Dave, J. M., Olvera, N., Alford, S., & Thompson, D. (2020). Culinary education programs for children in low-income households: A scoping review. Children, 7(5), 47. | Excluded – No Interventions |
|  | Littlewood, R., Canfell, O. J., & Walker, J. L. (2020). Interventions to prevent or treat childhood obesity in Māori & Pacific Islanders: a systematic review. BMC public health, 20(1), 1-14. | Excluded - Not Qualitative |
|  | Lofton, S., Julion, W. A., McNaughton, D. B., Bergren, M. D., & Keim, K. S. (2016). A systematic review of literature on culturally adapted obesity prevention interventions for African American youth. The Journal of School Nursing, 32(1), 32-46. | Excluded - Not Qualitative |
|  | Lu, W., McKyer, E. L. J., Lee, C., Goodson, P., Ory, M. G., & Wang, S. (2014). Perceived barriers to children’s active commuting to school: a systematic review of empirical, methodological and theoretical evidence. International Journal of Behavioral Nutrition and Physical Activity, 11(1), 1-20. | Excluded - Not Qualitative |
|  | Ma, J., Lander, N., Eyre, E. L., Barnett, L. M., Essiet, I. A., & Duncan, M. J. (2021). It’s not just what you do but the way you do it: a systematic review of process evaluation of interventions to improve gross motor competence. Sports Medicine, 51(12), 2547-2569. | Excluded – Not Obesity Outcomes |
|  | Mack I, Bayer C, Schaeffeler N, Reiband N, Broelz E, Zurstiege G, Fernandez‐Aranda F, Gawrilow C, Zipfel S. Chances and limitations of video games in the fight against childhood obesity—A systematic review. European Eating Disorders Review. 2017 Jul;25(4):237-67. | Excluded - Not Qualitative |
|  | Zelenović M, Manić M, Stamenković A, Čaprić I, Božić D. Barriers to physical activity in adolescents: A systematic review. Turkish Journal of Kinesiology. 2021;7(1):22-30. | Excluded - Not Qualitative |
|  | Marks, R. (2015). Childhood obesity and parental health literacy. Advances in Obesity, Weight Management & Control, 3(3), 191-195. | Excluded – No Interventions |
|  | Marr, C., Reale, S., Breeze, P., & Caton, S. J. (2021). Grandparental dietary provision, feeding practices and feeding styles when caring for preschool‐aged grandchildren: a systematic mixed methods review. Obesity Reviews, 22(4), e13157. | Excluded – No Interventions |
|  | Marshall, S., Taki, S., Laird, Y., Love, P., Wen, L. M., & Rissel, C. (2021). Cultural adaptations of obesity‐related behavioral prevention interventions in early childhood: A systematic review. Obesity Reviews, e13402. | Excluded - Not Qualitative |
|  | Martins, J., Costa, J., Sarmento, H., Marques, A., Farias, C., Onofre, M., & Valeiro, M. G. (2021). Adolescents’ perspectives on the barriers and facilitators of physical activity: an updated systematic review of qualitative studies. International Journal of Environmental Research and Public Health, 18(9), 4954. | Excluded – No Interventions |
|  | Matizanadzo, J. T., & Paudyal, P. (2021). The delivery of obesity interventions to children and adolescents with physical disabilities: a systematic review. Journal of Public Health. | Excluded – Not Adolescent/Parent Perspectives |
|  | Mawia, M. B. Tin Tin Su, Nik Daliana Nik Farid, Meram Azzani, Parents’ Perception of Child Weight Status, Risk Factors and Health Concern of Childhood Obesity: A Systematic Review.(2020). Int. J. Life Sci. Pharma Res, 10(1), L15-32. | Excluded – No Interventions |
|  | McGill, B., Sweeting, J., Surkalim, D., Phongsavan, P., Thomas, M., & Bellew, W. (2020). New developments in the prevention of obesity among children and young people aged 0-18 Years: Rapid Evidence Review Update. New developments in the prevention of obesity among children and young people aged 0-18 years-Rapid evidence review update. | Excluded – Rapid Review of Reviews only |
|  | Messiah SE, Sacher PM, Yudkin J, Ofori A, Qureshi FG, Schneider B, Hoelscher DM, De la Cruz-Munoz N, Barlow SE. Application and effectiveness of eHealth strategies for metabolic and bariatric surgery patients: a systematic review. Digital health. 2020 Jan;6:2055207619898987. | Excluded – Not Adolescents |
|  | Militello LK, Kelly S, Melnyk BM, Smith L, Petosa R. A review of systematic reviews targeting the prevention and treatment of overweight and obesity in adolescent populations. Journal of Adolescent Health. 2018 Dec 1;63(6):675-87. | Excluded – Not Adolescent/Parent Perspectives |
|  | Moxthe, L. C., Sauls, R., Ruiz, M., Stern, M., Gonzalvo, J., & Gray, H. L. (2020). Effects of bariatric surgeries on male and female fertility: a systematic review. Journal of Reproduction & Infertility, 21(2), 71. | Excluded - Not Adolescents |
|  | Munt, A. E., Partridge, S. R., & Allman‐Farinelli, M. (2017). The barriers and enablers of healthy eating among young adults: A missing piece of the obesity puzzle: A scoping review. Obesity reviews, 18(1), 1-17. | Excluded – Not Adolescents |
|  | Muzaffar, H., Metcalfe, J. J., & Fiese, B. (2018). Narrative review of culinary interventions with children in schools to promote healthy eating: directions for future research and practice. Current developments in nutrition, 2(6), nzy016. | Excluded – No Interventions |
|  | Ng CY, Thomas-Uribe M, Yang YA, Chu MC, Liu SD, Pulendran UP, Lin BJ, Lerner DS, King AC, Wang CJ. Theory-based health behavior interventions for pediatric chronic disease management: a systematic review. JAMA pediatrics. 2018 Dec 1;172(12):1177-86. | RCTs only |
|  | Nørnberg, T. R., Houlby, L., Skov, L. R., & Peréz-Cueto, F. J. A. (2016). Choice architecture interventions for increased vegetable intake and behaviour change in a school setting: a systematic review. Perspectives in public health, 136(3), 132-142. | Excluded - Not Obesity |
|  | Norris, E., Hamer, M., & Stamatakis, E. (2016). Active video games in schools and effects on physical activity and health: a systematic review. The Journal of Pediatrics, 172, 40-46. | Excluded - Not Qualitative |
|  | O’Connor, T., et al. (2018). "Engaging Latino Fathers in Children’s Eating and Other Obesity-Related Behaviors: a Review." Current Nutrition Reports 7(2): 29-38. | Excluded – No Data |
|  | Ochieng, M. A. (2020). School nurses’ nursing interventions in the prevention of childhood obesity. PhD Thesis | Excluded – Not Adolescent/Parent Perspectives |
|  | Ochoa, A. and J. M. Berge (2017). "Home Environmental Influences on Childhood Obesity in the Latino Population: A Decade Review of Literature." Journal of Immigrant and Minority Health 19(2): 430-447. | Excluded – No Interventions |
|  | Owusu, M, A Systematic Review Of The Interactions Between And Characteristics Associated With Obesity And Depressive Symptoms In The Pediatric Population" (2013). Yale Medicine Thesis Digital Library. 1827.  <https://elischolar.library.yale.edu/ymtdl/1827>  Dissertation Abstracts International: Section B: The Sciences and Engineering 74(12-B(E)). | Longitudinal cohort studies, cross-sectional studies of cohorts and intervention studies looking for treatment-associated changes |
|  | Ozodiegwu, I. D., et al. (2019). "A qualitative research synthesis of contextual factors contributing to female overweight and obesity over the life course in sub-Saharan Africa." PLoS ONE [Electronic Resource] 14(11): e0224612. | Excluded – No Interventions |
|  | Paes, V. M., et al. (2015). "Factors influencing obesogenic dietary intake in young children (0-6 years): Systematic review of qualitative evidence." BMJ Open 5(9). | Excluded – No Interventions |
|  | Park SH. Asian parents’ perception of child weight status: a systematic review. Journal of Child and Family Studies. 2017 Sep;26(9):2363-73. | Excluded – No Interventions |
|  | Patel, T., Umeh, K., Poole, H., Vaja, I., & Newson, L. (2021). Cultural identity conflict informs engagement with self-management behaviours for South Asian patients living with type-2 diabetes: A critical interpretative synthesis of qualitative research studies. International journal of environmental research and public health, 18(5), 2641. | Excluded – Not Adolescents |
|  | Pereira AI, Barros L. Parental cognitions and motivation to engage in psychological interventions: A systematic review. Child Psychiatry & Human Development. 2019 Jun;50(3):347-61. | Excluded – Not Adolescents |
|  | Pike KM, Dunne PE, Addai E. Expanding the boundaries: Reconfiguring the demographics of the “typical” eating disordered patient. Current psychiatry reports. 2013 Nov;15(11):1-8. | Excluded – Not Adolescent/Parent Perspectives |
|  | Pocock, M., et al. (2010). "Parental perceptions regarding healthy behaviours for preventing overweight and obesity in young children: a systematic review of qualitative studies." Obesity Reviews 11(5): 338-353. | Excluded – No Interventions |
|  | Pulgaron, E. R., et al. (2016). "Grandparent Involvement and Children's Health Outcomes: The Current State of the Literature." Families Systems & Health 34(3): 260-269. | Excluded – No Interventions |
|  | Pulimeno, Manuela et al. “Children's literature to promote students' global development and wellbeing.” Health promotion perspectives vol. 10,1 13-23. 28 Jan. 2020, doi:10.15171/hpp.2020.05 | Excluded - No Interventions |
|  | Redsell SA, Slater V, Rose J, Olander EK, Matvienko‐Sikar K. Barriers and enablers to caregivers' responsive feeding behaviour: A systematic review to inform childhood obesity prevention. Obesity Reviews. 2021 Jul;22(7):e13228. | Excluded – No Interventions |
|  | Rees, R., et al. (2011). "The views of young children in the UK about obesity, body size, shape and weight: a systematic review." BMC Public Health 11: 188. | Excluded – No Interventions |
|  | Rees, R. W., et al. (2014). "'It's on your conscience all the time': a systematic review of qualitative studies examining views on obesity among young people aged 12-18 years in the UK." BMJ Open 4(4): e004404. | Excluded – No Interventions |
|  | Regber, S. and H. Jormfeldt (2019). Foster homes for neglected children with severe obesity-Debated but rarely studied. Acta Paediatrica 108(11): 1955-1964. | Excluded – No Interventions |
|  | Reilly, J. J., Hughes, A. R., Gillespie, J., Malden, S., & Martin, A. (2019). Physical activity interventions in early life aimed at reducing later risk of obesity and related non‐communicable diseases: A rapid review of systematic reviews. Obesity Reviews, 20, 61-73. | Excluded - Not Qualitative |
|  | Roberts, S. H., & Bailey, J. E. (2011). Incentives and barriers to lifestyle interventions for people with severe mental illness: a narrative synthesis of quantitative, qualitative and mixed methods studies. Journal of advanced nursing, 67(4), 690-708. | Excluded – Not Adolescents |
|  | Robertson C, Archibald D, Avenell A, Douglas F, Hoddinott P, van Teijlingen E, Boyers D, Stewart F, Boachie C, Fioratou E, Wilkins D. Systematic reviews of and integrated report on the quantitative, qualitative and economic evidence base for the management of obesity in men. Health Technology Assessment (Winchester, England). 2014 May;18(35):v. | Excluded – Not Adolescents |
|  | Ronto R, Rathi N, Worsley A, Sanders T, Lonsdale C, Wolfenden L. Enablers and barriers to implementation of and compliance with school-based healthy food and beverage policies: a systematic literature review and meta-synthesis. Public health nutrition. 2020 Oct;23(15):2840-55. | Excluded - No Interventions |
|  | Rose K, O'Malley C, Eskandari F, Lake AA, Brown L, Ells LJ. The impact of, and views on, school food intervention and policy in young people aged 11–18 years in Europe: a mixed methods systematic review. Obesity Reviews. 2021 May;22(5):e13186. | Excluded - No Interventions |
|  | Russell G, Laws R, Campbell K, Lynch J, Ball K, Denney-Wilson E. Parental influences on weight gain in infants and young children from disadvantaged families. Obesity Research & Clinical Practice. 2013(7):e122-3. | Excluded - No Interventions |
|  | Rylatt L, Cartwright T. Parental feeding behaviour and motivations regarding pre-school age children: A thematic synthesis of qualitative studies. Appetite. 2016 Apr 1;99:285-97. | Excluded - No Interventions |
|  | Sabate J, Wien M. Vegetarian diets and childhood obesity prevention. The American journal of clinical nutrition. 2010 May 1;91(5):1525S-9S. | Excluded - Not Qualitative |
|  | Sahota P, Wordley J, and Woodward J. Effective behavioural components in child and adolescent weight management programmes. *Obesity Reviews* 2011 Vol. 1) Pages 57-58 | Excluded – Conference Abstract |
|  | Scott-Sheldon, L.A., Hedges, L.V., Cyr, C., Young-Hyman, D., Khan, L.K., Magnus, M., King, H., Arteaga, S., Cawley, J., Economos, C.D. and Haire-Joshu, D., 2020. Childhood Obesity Evidence Base Project: A systematic review and meta-analysis of a new taxonomy of intervention components to improve weight status in children 2–5 years of age, 2005–2019. Childhood Obesity, 16(S2), pp.S2-21. | Excluded - Not Qualitative |
|  | Singh A, Bassi S, Nazar GP, Saluja K, Park M, Kinra S, Arora M. Impact of school policies on non-communicable disease risk factors–a systematic review. BMC public health. 2017 Dec;17(1):1-9. | Excluded - No Interventions |
|  | Skelton, J. A., et al. (2014). "A systematic review of satisfaction and pediatric obesity treatment: new avenues for addressing attrition." Journal for healthcare quality : official publication of the National Association for Healthcare Quality 36(4): 5-22. | Excluded from Review 3 (no exercise data) |
|  | Sosa, E. T. (2010). "Mexican American mothers' perceptions of childhood obesity and their role in prevention." Dissertation Abstracts International Section A: Humanities and Social Sciences 71(3-A). | Excluded – No Interventions |
|  | Sosa, E. T. (2012). "Mexican American mothers' perceptions of childhood obesity: a theory-guided systematic literature review." Health Educ Behav 39(4): 396-404. | Excluded – No Interventions |
|  | Stephen, A., Bermano, G., Bruce, D., & Kirkpatrick, P. (2013). Competencies and skills to enable effective care of severely obese patients undergoing bariatric surgery across a multi-disciplinary health care perspective: a systematic review protocol. JBI Evidence Synthesis, 11(8), 84-96. | Excluded - Protocol |
|  | Sutcliffe, K., Richardson, M., Rees, R., Melendez-Torres, G.J., Stansfield, C., Thomas, J., 2016. What are the Critical Features of Successful Tier 2 Weight Management Programmes?: A Systematic Review to Identify the Programme Characteristics, and Combinations of Characteristics, That are Associated with Successful Weight Loss. EPPI-Centre, UCL, London. | Excluded – Not Adolescents |
|  | Sutcliffe K., Melendez-Torres G.J., Burchett H.E.D., Richardson M., Rees R., Thomas J., The importance of service users’ perspectives: a systematic review of qualitative evidence reveals overlooked critical features of weight management programmes, Health Expect., in preparation. | Excluded – Not Adolescents |
|  | Trübswasser, U., et al. (2020). "Factors influencing obesogenic behaviours of adolescent girls and women in low- and middle-income countries: A qualitative evidence synthesis." Obesity Reviews. | Excluded – No Interventions |
|  | van der Kleij RM, Coster N, Verbiest M, Van Assema P, Paulussen T, Reis R, Crone M. Implementation of intersectoral community approaches targeting childhood obesity: a systematic review. obesity reviews. 2015 Jun;16(6):454-72. | Excluded - No Interventions |
|  | Vanessa Augusta Souza, B., et al. (2017). "Nursing interventions with people with obesity in Primary Health Care: an integrative review." Revista da Escola de Enfermagem da USP 51: 1-10. | Excluded from Review 3 (no exercise data) |
|  | Warr W, Aveyard P, Albury C, Nicholson B, Tudor K, Hobbs R, Roberts N, Ziebland S. A systematic review and thematic synthesis of qualitative studies exploring GPs' and nurses' perspectives on discussing weight with patients with overweight and obesity in primary care. Obes Rev. 2021 Apr;22(4):e13151. doi: 10.1111/obr.13151. Epub 2020 Dec 6. PMID: 33283435; PMCID: PMC7988601. | Excluded - Health professional perspectives |
|  | White B, Doyle J, Colville S, Nicholls D, Viner RM, Christie D. Systematic review of psychological and social outcomes of adolescents undergoing bariatric surgery, and predictors of success. Clinical obesity. 2015 Dec;5(6):312-24. | Excluded - Not Qualitative |
|  | Williamson C, Baker G, Mutrie N, Niven A, Kelly P. Get the message? A scoping review of physical activity messaging. International Journal of Behavioral Nutrition and Physical Activity. 2020 Dec;17(1):1-5. | Excluded – Not Adolescents |
