# Supplementary material for: Exploring the values and preferences of children and adolescents with obesity and their parents/caregivers concerning diet or physical activity interventions for weight management: Mega-ethnography of qualitative syntheses
Source: PLoS One. 2026 Jan 20;21(1):e0340875. doi: 10.1371/journal.pone.0340875 (PMC12818672; doi:10.1371/journal.pone.0340875)
Supplement: S2 Table — (DOCX) [file pone.0340875.s005.docx]

**S2 Table. Data extraction of included reviews (Dietary management)**

| **First Author (year of publication)** | **Date of data extraction** | **Extracted by** | **Checked by** | **Eligible for inclusion** | **Age of Children (review inclusion criteria)** | **Number of Qualitative studies** | **Data or Themes reported** |
| --- | --- | --- | --- | --- | --- | --- | --- |
| **Burchett (2018) [21]** | March- April 2022 | ME | CC | Y | 0-11 | 11 (11) | Learning how to change: Practical experiences that show you how to change, not only telling you what to change |
|  |  |  |  |  | 0-11 | 11 (11) | Getting all the family ‘on-board’: Shared understanding and a healthy home environment |
|  |  |  |  |  | 0-11 | 11 (11) | Social support: a safe space with similar others in which to gain confidence and skills |
| **Jones (2019) [23]** | March- April 2022 | ME | CC | Y | 9-18 | 24 (28) | Tailored intervention |
|  |  |  |  |  | 9-18 | 24 (28) | Active engagement |
|  |  |  |  |  | 9-18 | 24 (28) | Support – Health workers support valued; Importance of family support; Peer support valued |
|  |  |  |  |  | 9-18 | 24 (28) | Barriers to attending an obesity management programme and being healthy - Prior fears of attending interventions |
|  |  |  |  |  | 9-18 | 24 (28) | Enjoyment from learning to eat healthily |
|  |  |  |  |  | 9-18 | 24 (28) | Motivations - Weight loss as primary motivation; Adolescents recognizing personal responsibility and personal motivation for weight loss |
|  |  |  |  |  | 9-18 | 24 (28) | Maintenance - Transferring skills learnt into a home environment and routine; Longer term support |
|  |  |  |  |  | 9-18 | 24 (28) | Technology |
| **Kebbe (2017) [24]** | March- April 2022 | ME | CC | Y | 2-18 | 11 (17) | Barriers: Nutrition – Individual - Autonomy and behaviour control |
|  |  |  |  |  | 2-18 | 11 (17) | Barriers: Nutrition – Interpersonal - Family and social network |
|  |  |  |  |  | 2-18 | 11 (17) | Barriers: Nutrition – Environmental - Home environment |
|  |  |  |  |  | 2-18 | 11 (17) | Enablers: Nutrition – Individual - Biological and cognitive factors |
|  |  |  |  |  | 2-18 | 11 (17) | Enablers: Nutrition – Interpersonal - Family, professional and social network |
|  |  |  |  |  | 2-18 | 11 (17) | Enablers: Nutrition – Environmental - Home environment |
| **Kelleher (2017) [25]** | March- April 2022 | ME | CC | Y | 2-18 | 6 (13) | Modifiable factors influencing initial attendance – Facilitators - Lifestyle-focused approach |
|  |  |  |  |  | 2-18 | 6 (13) | Modifiable factors influencing continued attendance Facilitators - Family-centred approach |
|  |  |  |  |  | 2-18 | 6 (13) | Modifiable factors influencing continued attendance – Barriers - Personal and programme logistics |
| **Lachal (2013) [26]** | March- April 2022 | ME | CC | Y | 0-18 | 45 (45) | Understanding others, understanding oneself - This axis of experience demonstrates how each different group understands obesity. |
|  |  |  |  |  | 0-18 | 45 (45) | Treating others, treating oneself- Overall understanding of the provision of care |
|  |  |  |  |  | 0-18 | 45 (45) | Treating others, treating oneself- Subjective evaluation of treatment |
| **Lang (2020) [27]** | March- April 2022 | ME | CC | Y | 2-18 | 16 (16) | Intrapersonal factor: Motivation versus ambivalence towards change |
|  |  |  |  |  | 2-18 | 16 (16) | Intrapersonal factor: Managing the challenges of change |
|  |  |  |  |  | 2-18 | 16 (16) | Intrapersonal factor: Relationships with peers |
|  |  |  |  |  | 2-18 | 16 (16) | Intrapersonal factor: Relationships with health care workers |
|  |  |  |  |  | 2-18 | 16 (16) | Interpersonal factor: Family dynamics |
|  |  |  |  |  | 2-18 | 16 (16) | Intrapersonal factor: Family support |
|  |  |  |  |  | 2-18 | 16 (16) | Institutional factor: Educational institutions/ employment |
|  |  |  |  |  | 2-18 | 16 (16) | Community factors and public policy: The broader environment |
| **Liu (2021) [28]** | March- April 2022 | ME | CC | Y | 9-18 | 48 (48) | Nutrition education |
|  |  |  |  |  | 9-18 | 48 (48) | Child involvement |
|  |  |  |  |  | 9-18 | 48 (48) | Family illness experience |
|  |  |  |  |  | 9-18 | 48 (48) | Family health |
|  |  |  |  |  | 9-18 | 48 (48) | Cultivation of preference |
|  |  |  |  |  | 9-18 | 48 (48) | Family motivation |
|  |  |  |  |  | 9-18 | 48 (48) | Food preparation and availability |
|  |  |  |  |  | 9-18 | 48 (48) | Time and cost |
|  |  |  |  |  | 9-18 | 48 (48) | Parenting style |
|  |  |  |  |  | 9-18 | 48 (48) | Parental practical knowledge and attitudes |
| **Molina (2021) [20]** | March- April 2022 | ME | CC | Y | NR | 10 (44) | Primary health care promotion of healthy eating |
|  |  |  |  |  | NR | 10 (44) | Improvement of school nutrition and physical activity environments |
|  |  |  |  |  | NR | 10 (44) | Fiscal policies and regulation of food marketing and labelling |
| **Roberts (2021) [29]** | March- April 2022 | ME | CC | Y | 2-18 | 9 (12) | Barriers to treatment: Structural |
|  |  |  |  |  | 2-18 | 9 (12) | Barriers to treatment: Financial and patient and family |
|  |  |  |  |  | 2-18 | 9 (12) | Barriers to treatment: Personal behaviors, motivation and expectations |
|  |  |  |  |  | 2-18 | 9 (12) | Facilitators of treatment: Structural |
| **Zarnowiecki (2020) [30]** | March- April 2022 | ME | CC | Y | >1 years | 9 (35) | Preferred content |
|  |  |  |  |  | >1 years | 9 (35) | Preferred features and functionality |
|  |  |  |  |  | >1 years | 9 (35) | Functionality and delivery mode |
|  |  |  |  |  | >1 years | 9 (35) | Use ability, appeal and barriers |
