# Supplementary material for: Exploring the values and preferences of children and adolescents with obesity and their parents/caregivers concerning diet or physical activity interventions for weight management: Mega-ethnography of qualitative syntheses
Source: PLoS One. 2026 Jan 20;21(1):e0340875. doi: 10.1371/journal.pone.0340875 (PMC12818672; doi:10.1371/journal.pone.0340875)
Supplement: S3 Table — (DOCX) [file pone.0340875.s006.docx]

**Table S3. Data extraction of included reviews (exercise/physical activity management)**

| **First Author (year of publication)** | **Date of data extraction** | **Extracted by** | **Checked by** | **Eligible for inclusion** | **Age of Children (review inclusion criteria)** | **Number of Qualitative studies** | **Data or Themes reported** |
| --- | --- | --- | --- | --- | --- | --- | --- |
| **Burchett (2018) [21]** | March- April 2022 | CC | ME | Y | 0-11 | 11 (11) | Learning how to change: **Practical experiences** that **show you** how to change, not only telling you what to change |
|  |  |  |  |  | 0-11 | 11 (11) | **Practical physical activity** sessions were widely and emphatically praised for giving children confidence and enabling them to experience enjoyment of being active |
| **Haracz (2013) [22]** | March- April 2022 | CC | ME | Y | 9-18 | 2 (22) | Focus of occupational therapy intervention - Increasing physical activity participation |
| **Jones, (2019) [23]** | March- April 2022 | CC | ME | Y | 9-18 | 24 (28) | Active engagement |
|  |  |  |  |  | 9-18 | 24 (28) | Physical activity vs. diet |
|  |  |  |  |  | 9-18 | 24 (28) | Motivations |
|  |  |  |  |  | 9-18 | 24 (28) | Maintenance |
| **Kebbe (2017) [24]** | March- April 2022 | CC | ME | Y | 2-18 | 11 (17) | Barriers: Physical Activity – Individual |
|  |  |  |  |  | 2-18 | 11 (17) | Barriers: Physical Activity – Interpersonal |
|  |  |  |  |  | 2-18 | 11 (17) | Barriers: Physical Activity – Environmental |
|  |  |  |  |  | 2-18 | 11 (17) | Enablers: Physical activity |
| **Kelleher, (2017) [25]** | March- April 2022 | CC | ME | Y | 2-18 | 6 (13) | Modifiable factors influencing initial attendance - Facilitators |
|  |  |  |  |  | 2-18 | 6 (13) | Modifiable factors influencing continued attendance - Facilitators |
| **Lachal (2013) [26]** | March- April 2022 | CC | ME | Y | 0-18 | 45 (45) | Treating others, treating oneself- Overall understanding of the provision of care |
|  |  |  |  |  | 0-18 | 45 (45) | Treating others, treating oneself- Subjective evaluation of treatment |
| **Lang (2020) [27]** | March- April 2022 | CC | ME | Y | 2-18 | 16 (16) | Motivation versus ambivalence towards change |
|  |  |  |  |  | 2-18 | 16 (16) | Managing the challenges of change |
|  |  |  |  |  | 2-18 | 16 (16) | Relationships with peers |
|  |  |  |  |  | 2-18 | 16 (16) | Relationships with health care workers |
|  |  |  |  |  | 2-18 | 16 (16) | Family support |
|  |  |  |  |  | 2-18 | 16 (16) | Educational institutions/ employment |
|  |  |  |  |  | 2-18 | 16 (16) | The broader environment |
| **Roberts (2021) [29]** | March- April 2022 | CC | ME | Y | 2-18 | 9 (12) | Financial and patient and family |
|  |  |  |  |  | 2-18 | 9 (12) | Personal behaviors, motivation and expectations |
| **Stankov (2012) [19]** | March- April 2022 | CC | ME | Y | 9-18 | 15 (15) | Regulatory environment |
|  |  |  |  |  | 9-18 | 15 (15) | Built environment |
|  |  |  |  |  | 9-18 | 15 (15) | Inhibitory social norms |
|  |  |  |  |  | 9-18 | 15 (15) | Physical environment |
|  |  |  |  |  | 9-18 | 15 (15) | Nature of household |
|  |  |  |  |  | 9-18 | 15 (15) | Lack of social support |
|  |  |  |  |  | 9-18 | 15 (15) | Negative body image |
|  |  |  |  |  | 9-18 | 15 (15) | Perceived inferiority in social settings |
|  |  |  |  |  | 9-18 | 15 (15) | Perceived victimization |
|  |  |  |  |  | 9-18 | 15 (15) | Lack of motivation |
|  |  |  |  |  | 9-18 | 15 (15) | Lack of knowledge |
