# Supplementary material for: Exploring the values and preferences of children and adolescents with obesity and their parents/caregivers concerning diet or physical activity interventions for weight management: Mega-ethnography of qualitative syntheses
Source: PLoS One. 2026 Jan 20;21(1):e0340875. doi: 10.1371/journal.pone.0340875 (PMC12818672; doi:10.1371/journal.pone.0340875)
Supplement: S4 Table — (DOCX) [file pone.0340875.s007.docx]

**Table S4. Quality assessment of included reviews**

|  | **Burchett 2018 [21]** | **Chen 2024 [31]** | **Jones 2019 [23]** | **Kebbe 2017 [24]** | **Kelleher 2017 [25]** | **Lachal 2013 [26]** | **Lang 2021 [27]** | **Roberts 2021 [29]** | **Haracz 2013 [22]** | **Stankov 2014 [19]** | **Skogen 2022[32]** | **Liu 2021 [28]** | **Molina 2021 [20]** | **Zarnowiecki 2020 [30]** | |
| --- | --- | --- | --- | --- | --- | --- | --- | --- | --- | --- | --- | --- | --- | --- | --- |
| 1. **Aim**   **Was the research question clearly stated?** | L | M | L | L | L | L | M | L | M | L | L | L | L | L | |
| 1. **Search approach**   **Was the approach to searching for the literature appropriate for the research question?** | M | L | L | L | L | L | L | L | L | L | L | L | L | L | |
| 1. **Inclusion criteria**   **Were the inclusion/exclusion criteria clearly described?** | L | L | L | L | L | L | L | L | L | L | L | L | M | L | |
| 1. **Competence**   **Were there a sufficient number of researchers involved in the synthesis who had adequate competence?** | M | M | M | L | M | M | L | H | L | M | L | M | M | H | |
| 1. **Search strategy**   **Was the search strategy sufficient to capture the relevant literature?** | M | L | L | L | L | L | M | M | M | L | L | M | H | L | |
| 1. **Study screening**   **Was the selection of relevant studies conducted independently by more than one reviewer and with consensus?** | L | L | L | L | M | L | L | H | L | M | L | L | M | L | |
| 1. **Appraisal tool**   **Was risk of bias (or methodological quality) formally assessed using appropriate criteria?** | H | L | L | M | L | L | L | H | L | L | H | M | H | M | |
| 1. **Appraisal process**   **Was the appraisal conducted independently by more than one reviewer and with consensus?** | H | L | L | L | L | M | L | H | H | L | H | H | M | L | |
| 1. **Synthesis (method appropriateness)**   **Was the synthesis method appropriate for the research question?** | L | L | L | L | L | L | H | M | L | L | M | L | L | M | |
| 1. **Synthesis process**   **Was the synthesis conducted appropriately?** | L | L | L | L | M | L | H | M | M | L | M | L | L | M | |
| 1. **Synthesis output:**   **Were findings clearly grounded in the primary studies?** | L | L | L | L | L | L | H | L | H | L | L | L | L | H | |
| 1. **Synthesis output:**   **Did the synthesized result go beyond a summary of results from the included studies?** | H | M | M | L | L | M | L | L | M | L | M | L | H | | H |
| 1. **Confidence in finding (CERQual)**   **Was the confidence in the findings assessed with GRADE-CERQual in an appropriate way?** | H | H | L | H | H | H | H | H | H | H | H | H | H | H | |
| **Overall verdict (concerns)**  **What is your Overall Assessment?** | Moderate | Moderate | Minor | Minor | Minor | Minor | Minor | High | Moderate | Minor | Moderate | Moderate | Moderate | High | |
| **Data richness score** | 2 | 2 | 3 | 1 | 1 | 3 | 2 | 1 | 1 | 2 | 1 | 3 | 1 | 1 | |

**Notes:**

**Burchett 2018 [19]:** Search was a combination of screening existing reviews and updating two reviews' searches - these previous reviews might have missed relevant items; detailed findings from the 'views synthesis' are not reported, only the approach and the statement that the findings went beyond the included studies; no critical appraisals were conducted; no CERQual

**Chen 2024[31]:** Search strategy provided. Did not synthesize results beyond summary.

**Jones 2019 [23]:** Only queries over the competence of researchers and unclear if findings are any more than a summary of the included studies

**Kebbe 2017 [24]:** Search strategy provided, results synthesized adequately, QA undertaken.

**Kelleher 2017 [23]:** Scoring was used in QA

**Lachal 2013 [26]:** Systematic Literature review of qualitative studies; this meta-synthesis is based on the procedure described by Atkins et al., adapted from meta-ethnography.

**Lang 2021 [27]:** Full technical search details not provided

**Roberts 2021 [29]:** No reference to any more than 1 reviewer performing tasks; no validated QLT study filter used; comprehensive multi-method search strategy, but complexities of creating comprehensive terms for 'clinic-based' definition might mean some relevant studies are missed; no critical appraisal performed - justification: scoping review

**Haracz 2013 [22]:** Each of the themes draws on discussion and recommendations for practice rather than study findings.

**Stankov 2014 [19]:** No research question, experience level not provided, consensus in screening NR, no CREQUAL

**Skogen 2022[32]:** Details on QA not provided. The approach used for synthesis is not clear. No CREQUAL

**Liu 2021 [28]:** Scoring was used in QA

**Molina 2021 [20]:** Inclusion criteria are only minimally stated, and there are no exclusion criteria. The number of reviewers involved in sifting is not reported. Data extracted by one reviewer and 'reviewed' by another. No statement of competences.

**Zarnowiecki 2020 [30]:** Scoring was used in QA, the approach used for synthesis is not clear
