# Supplementary material for: Exploring the values and preferences of children and adolescents with obesity and their parents/caregivers concerning diet or physical activity interventions for weight management: Mega-ethnography of qualitative syntheses
Source: PLoS One. 2026 Jan 20;21(1):e0340875. doi: 10.1371/journal.pone.0340875 (PMC12818672; doi:10.1371/journal.pone.0340875)
Supplement: S5 Table — (DOCX) [file pone.0340875.s008.docx]

**Table S5. Summary of Qualitative findings: Perceptions of the value (diet and physical activity interventions)**

| **First Author (year of publication)** | **Age of Children** | **Number of Qualitative studies** | **Third order constructs** | **Fourth order constructs** | **Illustrative quotations** |
| --- | --- | --- | --- | --- | --- |
| **Chen (2024) [31]** | 6-18 | 15 (31) | Individual constraints: Lack of motivation | Children and adolescents can be motivated to engage with obesity health services that focus on diet or physical activity interventions because of the perceived benefits of such activities   - Benefits: weight loss - Benefits: more than just weight loss - Benefits: can differ by gender - Motivations: Observing success in others | No quotations |
| **Chen (2024) [31]** | 6-18 | 15 (31) | Individual constraints: Psychological sensitivity and vulnerability |  |  |
| **Lachal (2013) [26]** | 0-18 | 45 (45) | Treating others, treating oneself- Overall understanding of the provision of care |  |  |
| **Lang (2021) [27]** | 2-18 | 16 (16) | Motivation versus ambivalence towards change |  |  |
| **Kebbe (2017) [24]** | 2-18 | 11 (17) | Enablers: Physical activity  Enablers: Nutrition – Interpersonal - Family, professional and social network |  |  |
| **Jones, (2019) [23]** | 9-18 | 24 (28) | Motivations - Weight loss as primary motivation; Barriers to attending a weight management programme and being healthy - Prior fears of attending interventions; Physical activity vs. Diet |  |  |
| **Lachal (2013) [26]** | 0-18 | 45 (45) | Treating others, treating oneself- Subjective evaluation of treatment |  |  |
| **Lang (2021) [27]** | 2-18 | 16 (16) | Motivation versus ambivalence towards change |  |  |
|  | 2-18 | 16 (16) | Intrapersonal factor: Managing the challenges of change |  |  |
| **Kelleher (2017) [25]** | 2-18 | 6 (13) | Modifiable factors influencing continued attendance – Facilitators - Social interaction and support; Practical sessions; Family-centred approach; Programme staff |  |  |
| **Liu (2021) [28]** | 9-18 | 48 (48) | Family illness experience;  Family health; Family motivation |  |  |
| **Roberts (2021) [29]** | 2-18 | 9 (12) | Barriers to treatment: Personal behaviours, motivation and expectations |  |  |
| **Skogen (2022) [32]** | 13-18 | 6 (12) | Reactions to and coping with victimization |  |  |
| **Skogen (2022) [32]** | 13-18 | 6 (12) | Relational victimization |  |  |
| **Chen (2024) [31]** | 6-18 | 15 (31) | Individual constraints: Lack of motivation | **Children and adolescents can view the idea of diet or physical activity interventions negatively, and question the value of such activities**   - Value of intervention questioned | **Lang 2020:**  “I can just eat really good foods and be really good but it never makes that much of a difference.”  **Kebbe 2017:**  ‘It’s difficult to explain, I just don’t like it’  ‘I think the weight caused [failure in physical education class]. Because I was overweight I didn’t want to make an effort. I didn’t want to try because I knew I wouldn’t be good at it’ [24][22](22)  «It’s not opportunity. I probably could, somehow, fit it in, but it’s just, I really can’t be bothered. I want to, but actually, I don’t at the same time. It’s like, I must go to the gym, and then I don’t bother.» (Daley et al., p. 815)  **Stankov 2012**:  “. . .bored and [that the exercise] has no purpose at all.”  “I really try and sometimes give up, but most of the time I watch. It is very hard” and “It seems like I’ve tried everything, watching my diet and exercising, and nothing works.”  “I spend so much time on the computer that I think I’ve already burned up a lot of calories and am doing exercise.” ([25], p.174)  “I gained weight gradually every year, like five or ten pounds. PE didn’t do anything for me.” ([27], p. 277) |
| **Chen (2024) [31]** | 6-18 | 15 (31) | Negative feedback |  |  |
| **Lachal (2013) [26]** | 0-18 | 45 (45) | Treating others, treating oneself- Subjective evaluation of treatment |  |  |
| **Lang (2021) [27]** | 2-18 | 16 (16) | Intrapersonal factor: Motivation versus ambivalence towards change |  |  |
| **Kebbe (2017) [24]** | 2-18 | 11 (17) | Barriers: Physical activity – individual |  |  |
| **Skogen 2022 [32]** | 13-18 | 6 (12) | Reactions to and coping with victimization |  |  |
| **Stankov (2012) [19]** | 9-18 | 15 (15) | Lack of knowledge |  |  |
