# Supplementary material for: Exploring the values and preferences of children and adolescents with obesity and their parents/caregivers concerning diet or physical activity interventions for weight management: Mega-ethnography of qualitative syntheses
Source: PLoS One. 2026 Jan 20;21(1):e0340875. doi: 10.1371/journal.pone.0340875 (PMC12818672; doi:10.1371/journal.pone.0340875)
Supplement: S6 Table — (DOCX) [file pone.0340875.s009.docx]

**Table S6. Summary of Qualitative findings: Competing priorities (diet and physical activity interventions)**

| **First Author (year of publication)** | **Age of Children** | **Number of Qualitative studies** | **Third order constructs** | **Fourth order constructs** | **Illustrative quotations** |
| --- | --- | --- | --- | --- | --- |
| **Kebbe (2017) [24]** | 2-18 | 11 (17) | Barriers: Physical Activity – Individual | **Individual children, adolescents and families have many commitments, and competing priorities can represent a problem when it comes to engaging in dietary or physical activity interventions**   - Opportunities: Absence of structured activities - Opportunities: Insufficient time (for exercise or time to prepare meals, engage and implement intervention, encourage and discuss healthy diet) | **Stankov 2012**:  “. . .if I exercise, I have to exercise for a long time to burn calories, and I think this will make me very tired  so that I cannot do other things.” (p.173)  “[Girls are] not interested in working like real hard doing all that exercise stuff. Some of them like – like girls they don’t like to sweat and get their hair messsed up. . .They think like they do the wrong thing, they break their nails, it’s a crisis.” ( p.5) |
|  | 2-18 | 11 (17) | Barriers: Physical Activity – Interpersonal |  |  |
|  | 2-18 | 11 (17) | Barriers: Physical Activity – Environmental |  |  |
|  | 2-18 | 11 (17) | Barriers: Nutrition – Individual - Autonomy and behaviour control; Biological and psychological factors; Mindless eating; Logistics |  |  |
| **Lang (2021) [27]** | 2-18 | 16 (16) | Educational institutions/ employment |  |  |
|  | 2-18 | 16 (16) | Intrapersonal factor: Family support |  |  |
|  | 2-18 | 16 (16) | Managing the challenges of change |  |  |
| **Liu (2021) [28]** | 9-18 | 48 (48) | Time and cost |  |  |
| **Kebbe (2017) [24]** | 2-18 | 11 (17) | Barriers: Physical Activity - Environmental |  |  |
| **Roberts (2021) [29]** | 2-18 | 9 (12) | Barriers to treatment: Financial and patient and family |  |  |
| **Stankov (2012) [19]** | 9-18 | 15 (15) | Physical environment |  |  |
