# Supplementary material for: Exploring the values and preferences of children and adolescents with obesity and their parents/caregivers concerning diet or physical activity interventions for weight management: Mega-ethnography of qualitative syntheses
Source: PLoS One. 2026 Jan 20;21(1):e0340875. doi: 10.1371/journal.pone.0340875 (PMC12818672; doi:10.1371/journal.pone.0340875)
Supplement: S7 Table — (DOCX) [file pone.0340875.s010.docx]

**Table S7. Summary of Qualitative findings: The role of social support (diet and physical activity interventions)**

| **First Author (year of publication)** | **Age of Children** | **Number of Qualitative studies** | **Third order constructs** | **Fourth order constructs** | **Illustrative quotations** |
| --- | --- | --- | --- | --- | --- |
| **Chen (2024) [31]** | 6-18 | 15 (31) | Interpersonal: Lack of family support | **Family involvement and support is important if children and adolescents are to engage with dietary or physical activity interventions**   - Concerns over parents’ and siblings’ levels of engagement with the intervention - Preferences for high levels of engagement with the intervention by parents and siblings - Competing priorities - Parents need to act as good role models by participating in exercise and physical activity - Reliance on parents to provide access to and means of doing adequate physical activity - Reliance on parents for resource to access and to provide means of healthy food including purchasing and preparing healthy meals - Barriers: Challenging family dynamics – conflicting opinions and approaches to lifestyle change | **Kebbe 2017**: ‘I don’t really go to gyms. At my mom’s house, I have a basketball court. When I work out and I do stuff, I usually like to do it with my cousin or by myself ... because then I don’t have to prove nothing to nobody. I do what I can do’. [24]  ‘If you’re just having something for dinner and it’s ... healthy or not healthy ... it’s not like you can change it necessarily because if that’s what ... is made at home, then that’s what you’re going to eat.’  **Kelleher 2017:** ‘One parent expressed their appreciation of having ‘something like that where it’s just her and I doing something together, just the two of us, I mean I thought that was great’ while another felt ‘it was good opportunity for my child and me to do something together’.  *‘*There was a time when my daughter would say, I don’t want to go, ’cause they’re telling me I can’t eat this and can’t eat that. And I go, No we’ll go, ’cause they’re telling me the same thing. When she saw it was difficult for me too and we started getting into a routine, she started wanting to go’*.*  **Burchett 2018: ‘**How can I tell her “this is what you need to do” if she's not seeing me do it.’  **Lang 2020**: “having my mom with me really motivates me.”(p1398)  As one young person explained “… I was gonna give up until my mom and my sister, they helped me keep going.”41(p1398)  “They've got to have the support of the others in the family otherwise it's almost impossible” Health professional (Staniford et al., 2011) p238  ‘Providers valued the active involvement of parents and carers and saw a family approach as crucial: “I think that's key… because if you don't change the parents, then nothing changes at home…”’ Provider (Lucas et al., 2014) p7  Shared responsibility for making changes “How can I tell her “this is what you need to do” if she's not seeing me do it” Parent (Watson, 2012) p118  “Children are more aware nowadays, none of us like being told to do things and so it was like forming a partnership and it worked” parent (Stewart, 2008) p166  “At home we've had different fruit in our house in our fruit bowl and we've had less chocolate” child (Watson, 2012) p124 |
| **Stankov (2012) [19]** | 9-18 | 15 (15) | Lack of social support |  |  |
| **Burchett (2018) [21]** | 0-11 | 11 (11) | Getting all the family ‘on-board’: Shared understanding and a healthy home environment |  |  |
| **Kebbe (2017) [24]** | 2-18 | 11 (17) | Barriers: Physical Activity – Interpersonal |  |  |
|  | 2-18 | 11 (17) | Barriers: Nutrition – Individual - Autonomy and behaviour control |  |  |
|  | 2-18 | 11 (17) | Barriers: Nutrition – Interpersonal - Family and social network |  |  |
|  | 2-18 | 11 (17) | Enablers: Nutrition – Interpersonal - Family, professional and social network |  |  |
|  | 2-18 | 11 (17) | Enablers: Nutrition – Environmental - Home environment |  |  |
| **Jones (2019) [23]** | 9-18 | 24 (28) | Support - Professional support valued; Importance of family support; Peer support valued |  |  |
|  | 9-18 | 24 (28) | Barriers to attending a weight management programme and being healthy - Prior fears of attending interventions |  |  |
|  | 9-18 | 24 (28) | Maintenance - Transferring skills learnt into a home environment and routine; Longer term support |  |  |
| **Kelleher, (2017) [25]** | 2-18 | 6 (13) | Modifiable factors influencing continued attendance - Facilitators |  |  |
|  | 2-18 | 6 (13) | Modifiable factors influencing continued attendance - Facilitators |  |  |
|  | 2-18 | 6 (13) | Modifiable factors influencing continued attendance Facilitators - Family-centred approach |  |  |
| **Lachal (2013) [26]** | 0-18 | 45 (45) | Treating others, treating oneself- Overall understanding of the provision of care |  |  |
| **Stankov (2012) [19]** | 9-18 | 15 (15) | Lack of social support |  |  |
| **Kebbe (2017) [24]** | 2-18 | 11 (17) | Enablers: Physical activity |  |  |
| **Lang (2021) [27]** | 2-18 | 16 (16) | Interpersonal factor: Family dynamics |  |  |
|  |  |  | Intrapersonal factor: Family support |  |  |
| **Roberts (2021) [29]** | 2-18 | 9 (12) | Barriers to treatment: Financial and patient and family |  |  |
| **Liu (2021) [28]** | 9-18 | 48 (48) | Nutrition education |  |  |
|  | 9-18 | 48 (48) | Family motivation |  |  |
|  | 9-18 | 48 (48) | Food preparation and availability |  |  |
|  | 9-18 | 48 (48) | Time and cost |  |  |
|  | 9-18 | 48 (48) | Parenting style |  |  |
| **Skogen 2022 [32]** | 13-18 | 6 (12) | Significant Others |  |  |
|  |  |  |  |  |  |
| **Chen (2024) [31]** | 6-18 | 15 (31) | Interpersonal: Negative feedback from society | **Health worker support is important if children and adolescents are to engage with dietary or physical activity interventions**   - Health workers need to provide supportive, structured guidance - Advice and support from health workers is valued - Concerns over health worker levels of engagement with the intervention - Health workers need to provide optimal care, be knowledgeable, share information and be empathetic - Barrier: Negative experience with health workers and lack of support - Concerns over stigmatization | **Lang 2020**: ‘One young person reflected that “I went to you know like my GP a couple of times to try and get advice on …what I should do … [was advised to] be mindful of what the intake was and perhaps to, to exercise regularly you know with, either with friends or you know try and get support you know. So that did help a lot.”.  ‘their problem was not being taken seriously, and felt rejected when asking for help.’  **Stankov 2012**: ‘in some cases [they] were even stereotyped by teachers who would say, “It’s a little too strenuous. . . you might want to sit this one out”.  **Kebbe 2017:** ‘... my mom and coach believe in me. They are great and supportive. I need the support to keep me going.’ |
| **Kebbe (2017) [24]** | 2-18 | 11 (17) | Enablers: Physical activity |  |  |
|  | 2-18 | 11 (17) | Enablers: Nutrition – Interpersonal - Family, professional and social network |  |  |
| **Lachal (2013) [26]** | 0-18 | 45 (45) | Treating others, treating oneself- Overall understanding of the provision of care |  |  |
| **Stankov (2012) [19]** | 9-18 | 15 (15) | Physical environment |  |  |
| **Lang (2021) [27]** | 2-18 | 16 (16) | Interpersonal Factor – relationships with healthcare professionals |  |  |
| **Jones (2019) [23]** | 9-18 | 24 (28) | Support - Professional support valued; Importance of family support; Peer support valued |  |  |
|  | 9-18 | 24 (28) | Barriers to attending a weight management programme and being healthy - Prior fears of attending interventions |  |  |
| **Roberts (2021) [29]** | 2-18 | 9 (12) | Facilitators of treatment: Structural |  |  |
|  | 2-18 | 9 (12) | Barriers to treatment: Structural |  |  |
| **Zarnowiecki (2020) [30]** | >1 years | 9 (35) | Preferred features and functionality |  |  |
| **Chen (2024) [31]** | 6-18 | 15 (31) | Interpersonal: Negative feedback from society | **Peer involvement and social support is important if children and adolescents are to engage with dietary or physical activity interventions**   - Friends and peers need to act as good role models by participating in exercise and physical activity - Friends and peers need to offer support - Friends and peers Involvement and engagement with the intervention makes individual feel a sense of belonging and being accepted - Barrier: Peer pressure, influence of bad behaviour from peers | **Lang 2020**: “your friend[s] are there, and they can motivate you to do better, too’.  ‘nobody eats breakfast at school. That is just not cool….’.  “In the gym, they laugh and talk behind my back,” and “It hurts me when they say ‘hey there, fat kid.’ I try to ignore them, but it does not stop.”.  **Stankov** **2012**: You’d be the first one out (on the Pacer test) and everyone would look down on you and be like, “huh, they can’t do it; they’re overweight.” ([27], p.281)  **Burchett 2018:** ‘I found them fun because I was surrounded by different people who were in the situation that I was in.’  “Coming here with other children similar to himself and getting to speak to other parents dealing with like the same issues is really helpful for us.”  “finding out you weren't alone in this […] having an open forum to say my kid does that too, cause you feel so guilty” [21][19](19)  **Kebbe 2017:** ‘your friend[s] are there, and they can motivate you to do better, too.”  “I don’t want the salad when my friends are eating Big Macs and French fries. It’s just not cool. They’d make fun of me.”  “I’m more self-conscious when I’m eating healthily than when I’m not, I feel like people look at me like you know because you’re fat you’re going to eat unhealthily but if you’re eating healthy, I think, I don’t know, I just, just feel it’s more of a big deal that you’re eating an apple or something, they like look and wonder why.” |
| **Kebbe (2017) [24]** | 2-18 | 11 (17) | Barriers: Physical Activity - Interpersonal |  |  |
|  | 2-18 | 11 (17) | Enablers: Nutrition – Interpersonal - Family, professional and social network |  |  |
| **Burchett (2018) [21]** | 0-11 | 11 (11) | Social support: a safe space with similar others in which to gain confidence and skills |  |  |
| **Jones (2019) [23]** | 9-18 | 24 (28) | Barriers to attending a weight management programme and being healthy - Prior fears of attending interventions |  |  |
|  | 9-18 | 24 (28) | Support - Professional support valued; Importance of family support; Peer support valued |  |  |
| **Kelleher (2017) [25]** | 2-18 | 6 (13) | Modifiable factors influencing continued attendance – Facilitators - Social interaction and support; Practical sessions; Family-centred approach; Programme staff |  |  |
| **Lang (2021) [27]** | 2-18 | 16 (16) | Intrapersonal factor: Relationships with peers |  |  |
| **Roberts (2021) [29]** | 2-18 | 9 (12) | Facilitators of treatment: Structural |  |  |
| **Skogen 2022 [32]** | 13-18 | 6 (12) | Physical activity with similar others |  |  |
| **Skogen 2022 [32]** | 13-18 | 6 (12) | Significant Others |  |  |
| **Stankov (2012) [19]** | 9-18 | 15 (15) | Lack of social support |  |  |
|  | 9-18 | 15 (15) | Negative body image |  |  |
|  | 9-18 | 15 (15) | Perceived victimization |  |  |
|  | 9-18 | 15 (15) | Lack of motivation |  |  |
|  | 9-18 | 15 (15) | Regulatory environment |  |  |
|  | 9-18 | 15 (15) | Negative body image / Perceived inferiority in social settings |  |  |
