# Supplementary material for: Exploring the values and preferences of children and adolescents with obesity and their parents/caregivers concerning diet or physical activity interventions for weight management: Mega-ethnography of qualitative syntheses
Source: PLoS One. 2026 Jan 20;21(1):e0340875. doi: 10.1371/journal.pone.0340875 (PMC12818672; doi:10.1371/journal.pone.0340875)
Supplement: S8 Table — (DOCX) [file pone.0340875.s011.docx]

**Table S8. Summary of Qualitative findings: The environment (exercise and physical activity interventions)**

| **First Author (year of publication)** | **Age of Children** | **Number of Qualitative studies** | **Third order constructs** | **Fourth order constructs** | **Illustrative quotations** |
| --- | --- | --- | --- | --- | --- |
| **Chen (2024) [31]** | 6-18 | 15 (31) | Environmental: Lack of policy safeguards | **Physical resources at school (spaces, equipment and facilities) need to be accessible and appropriate if children and adolescents are to engage with physical activity interventions**   - Appropriate changing and exercise facilities need to be accessible at time of intervention and beyond (school) | ‘“. . .the worst bit was getting changed and getting into the uniform for PE, ...’ [19]. |
| **Chen (2024) [31]** | 6-18 | 15 (31) | Environmental: Lack of built environment support |  |  |
| **Jones (2019) [23]** | 9-18 | 24 (28) | Maintenance |  |  |
| **Lang (2021) [27]** | 2-18 | 16 (16) | Educational institutions/ employment |  |  |
| **Stankov (2012) [19]** | 9-18 | 15 (15) | Regulatory environment |  |  |
|  | 9-18 | 15 (15) | Built environment |  |  |
|  | 9-18 | 15 (15) | Perceived inferiority in social settings |  |  |
| **Chen (2024) [31]** | 6-18 | 15 (31) | Environmental: Lack of built environment support | **Physical resources at home and in the community (safe spaces to play and exercise) need to be accessible and appropriate if children and adolescents are to engage with physical activity interventions**   - Appropriate exercise facilities need to be accessible at time of intervention and beyond (home and community) - Safety |  |
| **Kebbe (2017) [24]** | 2-18 | 11 (17) | Barriers: Physical Activity – Interpersonal |  | ‘I need someone to walk with me. My mom doesn’t want me walking around by myself. She says that she doesn’t trust the guys in the neighborhood’. |
|  | 2-18 | 11 (17) | Barriers: Physical Activity – Environmental |  |  |
| **Lang (2021) [27]** | 2-18 | 16 (16) | The broader environment |  |  |
| **Stankov (2012) [19]** | 9-18 | 15 (15) | Physical environment | **If the weather conditions are inappropriate to the type of planned physical activity, then children and adolescents may not be motivated to engage in physical activity**   - Opportunities: weather can limit scope for exercise and physical activity | No quotations |
| **Kebbe (2017) [24]** | 2-18 | 11 (17) | Barriers: Physical Activity – Environment |  |  |
| **Kelleher (2017) [25]** | 2-18 | 6 (13) | Modifiable factors influencing continued attendance – Barriers - Personal and programme logistics[more general about programme] | **Resource, programme and facilities at school, home and in the community need to be accessible for children, adolescents and their families to engage with dietary interventions**   - Accessibility to programmes and facilities: Clinic Location - Accessibility to programme and facilities: Timing and frequency of appointments - Accessibility to programme and facilities: Logistic and transport - Availability and access to healthy food: Options at school - Availability and access to healthy food: Social and cultural environment - Resource limitations: lack of internet, difficulty in using app, set-up time. | No quotations |
| **Lang (2021) [27]** | 2-18 | 16 (16) | Institutional factor: Educational institutions/ employment |  |  |
|  | 2-18 | 16 (16) | Community factors and public policy: The broader environment |  |  |
| **Roberts (2021) [29]** | 2-18 | 9 (12) | Barriers to treatment: Structural |  |  |
|  | 2-18 | 9 (12) | Facilitators of treatment: Structural |  |  |
| **Zarnowiecki (2020) [30]** | >1 years | 9 (35) | Use ability, appeal and barriers |  |  |
| **Liu (2021) [28]** | 9-18 | 48 (48) | Time and cost |  |  |
| **Molina (2021) [20]** | NR | 10 (44) | Improvement of school nutrition and physical activity environments |  |  |
|  | NR | 10 (44) | Multi-sectoral actions |  |  |
