# Supplementary material for: Exploring the values and preferences of children and adolescents with obesity and their parents/caregivers concerning diet or physical activity interventions for weight management: Mega-ethnography of qualitative syntheses
Source: PLoS One. 2026 Jan 20;21(1):e0340875. doi: 10.1371/journal.pone.0340875 (PMC12818672; doi:10.1371/journal.pone.0340875)
Supplement: S9 Table — (DOCX) [file pone.0340875.s012.docx]

**Table S9. Summary of Qualitative findings: The nature of the diet or physical activity interventions**

| **First Author (year of publication)** | **Age of Children** | **Number of Qualitative studies** | **Third order constructs** | **Fourth order constructs** | **Illustrative quotations** |
| --- | --- | --- | --- | --- | --- |
| **Kebbe (2017) [24]** | 2-18 | 11 (17) | Enablers: Physical activity | **The types of diet or physical activity on offer is important; they should be social, fun, interactive, informal and regular; healthy food should be palatable**  Characteristics of intervention:   - Informal and fun preferred over highly structured or competitive; preferences for social / team / group activities with peers and friends - Preferences for exercise and activity rather than diet as a means of weight management - Preferences for regular or frequent exercise or physical activity - Preferences for group activities and programmes with peers and friends - Preferences for Lifestyle focused intervention, which incorporated nutrition, physical activity and behavioural components. - Intervention should be structured with meal plans and recipe ideas - If intervention includes digital tools, it should be easy to use. - Healthy food should be palatable - Intervention should also include psychological support | **Burchett 2018:**  “It wasn't just like ‘you need to do more exercise and you need to eat better’ – it actually taught us like how to” child),  “The team games were good. Boost their confidence to join in with their mates. Cause some of these kids are really isolated so they need team sports to encourage them to join in” Parent).  “Coming here with other children similar to himself and getting to speak to other parents dealing with like the same issues is really helpful for us.” Parent (Staniford et al., 2011) p236  “I found them fun because I was surrounded by different people who were in the situation that I was in” Child (female) (Lucas et al., 2014) p8  “I think Im glad I stayed at it because I’ve made more friends and confidence has built up a bit…and it's easier to talk to people because they don't tell you to go away or you know ‘you're not fit to be with us because so and so and how you look’ but they actually go ‘oh hi, how are you today? And ‘do you want to come and join us’ ….’  “The portion sizes [session] was very good. We are eating way too much of everything and need to cut down.”  ‘The best bit I liked was making the bread.’ |
| **Jones (2019) [23]** | 9-18 | 24 (28) | Active engagement |  |  |
|  | 9-18 | 24 (28) | Physical activity vs. diet |  |  |
|  | 9-18 | 24 (28) | Barriers to attending a weight management programme and being healthy - Prior fears of attending interventions |  |  |
|  | 9-18 | 24 (28) | Enjoyment from learning to eat healthily |  |  |
| **Lachal (2013) [26]** | 0-18 | 45 (45) | Treating others, treating oneself- Subjective evaluation of treatment |  |  |
|  | 0-18 | 45 (45) | Treating others, treating oneself- Subjective evaluation of treatment |  |  |
| **Kelleher (2017) [25]** | 2-18 | 6 (13) | Modifiable factors influencing initial attendance – Facilitators - Lifestyle-focused approach |  |  |
|  | 2-18 | 6 (13) | Modifiable factors influencing continued attendance – Facilitators - Social interaction and support; Practical sessions; Family-centred approach; Programme staff |  |  |
| **Burchett (2018) [21]** | 0-11 | 11 (11) | Practical physical activity sessions were widely and emphatically praised for giving children confidence and enabling them to experience enjoyment of being active |  |  |
|  | 0-11 | 11 (11) | Learning how to change: Practical experiences that show you how to change, not only telling you what to change |  |  |
| **Kebbe (2017) [24]** | 2-18 | 11 (17) | Enablers: Physical activity |  |  |
| **Lang (2021) [27]** | 2-18 | 16 (16) | Relationships with peers |  |  |
| **Stankov (2012) [19]** | 9-18 | 15 (15) | Lack of social support |  |  |
| **Lachal (2013) [26]** | 0-18 | 45 (45) | Treating others, treating oneself- Subjective evaluation of treatment |  |  |
| **Lang (2021) [27]** | 2-18 | 16 (16) | Relationships with health care professionals |  |  |
| **Roberts (2021) [29]** | 2-18 | 9 (12) | Barriers to treatment: Structural |  |  |
| **Molina (2021) [20]** | NR | 10 (44) | Primary health care promotion of healthy eating |  |  |
|  | NR | 10 (44) | Fiscal policies and regulation of food marketing and labelling |  |  |
| **Zarnowiecki (2020) [30]** | Mix | 9 (35) | Use ability, appeal and barriers |  |  |
| **Liu (2021) [28]** | 9-18 | 48 (48) | Child involvement |  |  |
|  | 9-18 | 48 (48) | Cultivation of preference |  |  |
| **Stankov (2012) [19]** | 9-18 | 15 (15) | Regulatory environment | **The types of diet or physical activity on offer should be appropriate to the child in terms of their age, gender, ethnicity, culture and physical capabilities**  Characteristics of intervention   - Concerns over required clothing for activities - Perceived as too demanding by professionals and the children and adolescents themselves - Characteristics of intervention: Perceived as too difficult to do at home - Intervention need be tailored and personalised e.g. portion size, recipes, age appropriate - Dietary strategies need to be suited to the child/ adolescent and to their expectations | **Stankov 2012:**  “And I don’t want to wear a swimsuit. I asked him [teacher] nicely, ‘please do I have to wear a swimsuit?’ ‘Yes, you got to wear a swimsuit. . .’And the only swimsuit I got is one that shows my back and I don’t want it to show my rolls-that’s gross. And then people  going to call me Free Willy or something».’ and “I hate gym class. I hate wearing shorts. I feel so embarrassed about how I look in shorts.  ‘“. . .the worst bit was getting changed and getting into the uniform for PE, which was shorts.”’.  “I’m not very good at a lot of things we do in PE so I get embarrassed when we do those things.” ([34], p.52)  Boys reported similar experiences:  “When we’d have PE day at school and your whole school goes out to PE, I never won anything. I never  won the kickball contest. I never won the basketball shooting contest, I never won the Frisbee throw.” ([27], p.277)  “I think the weight caused [failure in PE]. Because I was overweight I didn’t want to make an effort. I  didn’t want to try because I knew I wouldn’t be good at it.” ([27], p.283)  “. . .you knew it was all because you were overweight. I hated going to PE.” ([27], p.280)  “I really try and sometimes give up, but most of the time I watch. It is very hard” and “It seems like I’ve tried everything, watching my diet and exercising, and nothing works.”  “. . .I like to ride a bicycle, but I can only do so for a short period of time because I feel very, very tired, and  I have no strength.” ([25], p.174)  **Lang 2020**: “…hard at the start, but see once you get into a routine of knowing what you do, what you can eat, what you can't eat … it is quite easy.” |
|  | 9-18 | 15 (15) | Lack of motivation |  |  |
| **Lachal (2013) [26]** | 0-18 | 45 (45) | Treating others, treating oneself- Subjective evaluation of treatment |  |  |
| **Jones (2019) [23]** | 9-18 | 24 (28) | Tailored intervention |  |  |
| **Roberts (2021) [29]** | 2-18 | 9 (12) | Barriers to treatment: Structural |  |  |
|  | 2-18 | 9 (12) | Facilitators of treatment: Structural |  |  |
| **Zarnowiecki (2020) [30]** | >1 years | 9 (35) | Preferred content |  |  |
| **Lang (2021) [27]** | 2-18 | 16 (16) | Intrapersonal factor: Managing the challenges of change |  |  |
| **Roberts (2021) [29]** | 2-18 | 9 (12) | Barriers to treatment: Personal behaviours, motivation and expectations |  |  |
| **Kelleher (2017) [25]** | 2-18 | 6 (13) | Modifiable factors influencing continued attendance – Facilitators | **Children and adolescents feel that it is important to ‘fit in’ and not to ‘stand out’**  Characteristics of intervention   - Positive experience of being with other children who are the same (normalization) - Social interaction with peers in group session makes the child feel accepted and a sense of belonging - Perceived as stigmatizing, self-conscious - Characteristics of intervention: adopting healthy eating practices with others, such as helping with portion control and sharing food | **Stankov 2012**:  “Everyone stares at you, you become the target when it’s PE, even more so, even if you’re not scared because you think that you’re going to become the target, and you know that you can’t do that area or whatever and you become more self-conscious at which point you get bullied more.”.  “you were classed, if like in the first year, you were decided that you were one of the outcasts, you weren’t the same as everybody else you were pushed to the outside and you weren’t let in . . .” ([30], p.414)  **Burchett 2018**: “finding out you weren't alone in this [...] having an open forum to say my kid does that too, cause you feel so guilty.”  “Coming here with other children similar to himself and getting to speak to other parents dealing with like the same issues is really helpful for us.” [21][19](19)  “I think I′m glad I stayed at it because I′ve made more friends and confidence has built up a bit...and it's easier to talk to people because they don't tell you to go away or you know ‘you're not fit to be with us because so and so and how you look’ but they actually go ‘oh hi, how are you today? And ‘do you want to come and join us’ ....” [21][19](19)  “I found them fun because I was surrounded by different people who were in the situation that I was in.” |
|  | 2-18 | 6 (13) | Modifiable factors influencing continued attendance – Facilitators |  |  |
| **Jones (2019) [23]** | 9-18 | 24 (28) | Support - Professional support valued; Importance of family support; Peer support valued |  |  |
| **Burchett (2018) [21]** | 0-11 | 11 (11) | Social support: a safe space with similar others in which to gain confidence and skills |  |  |
| **Jones (2019) [23]** | 9-18 | 24 (28) | Barriers to attending a weight management programme and being healthy - Prior fears of attending interventions |  |  |
| **Kebbe (2017) [24]** | 2-18 | 11 (17) | Barriers: Nutrition – Interpersonal - Family and social network |  |  |
|  | 2-18 | 11 (17) | Enablers: Nutrition – Interpersonal - Family, professional and social network |  |  |
| **Lang (2021) [27]** | 2-18 | 16 (16) | Intrapersonal factor: Motivation versus ambivalence towards change |  |  |
|  | 2-18 | 16 (16) | Intrapersonal factor: Relationships with peers |  |  |
| **Skogen (2022) [32]** | 13-18 | 6 (12) | Physical activity with similar others |  |  |
| **Stankov (2012) [19]** | 9-18 | 15 (15) | Negative self-image |  |  |
|  | 9-18 | 15 (15) | Perceived victimization |  |  |
| **Haracz (2013) [22]** | 9-18 | 2 (22) | Focus of occupational therapy intervention - Increasing physical activity participation | **The amount and type of advice on diet or physical activity interventions given to children and families is important (what activities might be best, how to do them, goals to aim for)**  Characteristics of intervention:   - Preferences for the involvement of specialist - Preference for evidence based, trusted and endorsed information - Use of positive language - Needs to give participants new knowledge and skills - Preferences for autonomy in choice of activities rather than the involvement of specialist providers during and after intervention - Practical sessions preferred to theory - Active engagement - Needs to give participants new knowledge and skills ( understanding the nutritional content of different foods and drinks as well as giving them a better awareness of what foods should be eaten in moderation) - Prescriptive and regulated diet routine set by a health worker - Use of concise, clear, consistent, direct and practical messages/advice - Need for emotional and knowledge support | **Kelleher 2017**: ‘I want to start exercising, but I don’t know what exercises to do’  ‘For example one mother reported that ‘...you don’t want to hear theory when you’re a mum. You want to hear real-life experience and what’s practical for us’  **Burchett 2018**:  “They're [specialist advisers) friendly and kind, and they boost your confidence.” Child (Lewis et al., 2014) p1222  “It wasn't just like ‘you need to do more exercise and you need to eat better’ – it actually taught us like how to” child (Watson, 2012) p181  “The team games were good. Boost their confidence to join in with their mates. Cause some of these kids are really isolated so they need team sports to encourage them to join in” Parent, (Pittson, 2013) p177 |
| **Stankov (2012) [19]** | 9-18 | 15 (15) | Lack of motivation |  |  |
| **Jones (2019) [23]** | 9-18 | 24 (28) | Motivations |  |  |
|  | 9-18 | 24 (28) | Maintenance |  |  |
|  | 9-18 | 24 (28) | Active engagement |  |  |
|  | 9-18 | 24 (28) | Enjoyment from learning to eat healthily |  |  |
| **Roberts (2021) [29]** | 2-18 | 9 (12) | Personal behaviors, motivation and expectations |  |  |
| **Kebbe (2017) [24]** | 2-18 | 11 (17) | Barriers: Physical Activity – Interpersonal |  |  |
|  | 2-18 | 11 (17) | Enablers: Physical activity |  |  |
|  | 2-18 | 11 (17) | Enablers: Nutrition |  |  |
| **Kelleher (2017) [25]** | 2-18 | 6 (13) | Modifiable factors influencing continued attendance - Facilitators |  |  |
| **Burchett (2018) [21]** | 0-11 | 11 (11) | Learning how to change: Practical experiences that show you how to change, not only telling you what to change |  |  |
| **Roberts (2021) [29]** | 2-18 | 9 (12) | Facilitators of treatment: Structural |  |  |
|  | 2-18 | 9 (12) | Barriers to treatment: Structural |  |  |
| **Zarnowiecki (2020) [30]** | >1 years | 9 (35) | Preferred content |  |  |
|  | >1 years | 9 (35) | Preferred features and functionality |  |  |
|  | >1 years | 9 (35) | Functionality and delivery mode |  |  |
| **Lang (2021) [27]** | 2-18 | 16 (16) | Interpersonal factor: Relationships with health care professionals |  |  |
