# Supplementary material for: Exploring the values and preferences of children and adolescents with obesity and their parents/caregivers concerning diet or physical activity interventions for weight management: Mega-ethnography of qualitative syntheses
Source: PLoS One. 2026 Jan 20;21(1):e0340875. doi: 10.1371/journal.pone.0340875 (PMC12818672; doi:10.1371/journal.pone.0340875)
Supplement: S10 Table — (DOCX) [file pone.0340875.s013.docx]

**Table S10. Summary of Qualitative findings: Cost of diet and physical activity interventions**

| **First Author (year of publication)** | **Age of Children (review inclusion criteria)** | **Number of Qualitative studies** | **Third order constructs** | **Fourth order constructs** | **Illustrative quotations** |
| --- | --- | --- | --- | --- | --- |

| **Roberts (2021) [29]** | 2-18 | 9 (12) | Barriers: Financial and patient and family | **Cost can be a real concern for children and their families when seeking to engage with dietray or physical activity interventions**  Characteristic of intervention   - Concerns over costs and logistics of attendance - Concerns over cost of healthy food (believes healthy food is more expensive) - Concern over cost of tools and apps to engage with intervention | No quotations |
| --- | --- | --- | --- | --- | --- |
|  | 2-18 | 9 (12) | Facilitators of treatment: Structural |  |  |
| **Kebbe (2017) [24]** | 2-18 | 11 (17) | Barriers: Nutrition – Environmental - Home environment |  |  |
| **Lang (2021) [27]** | 2-18 | 16 (16) | Community factors and public policy: The broader environment |  |  |
| **Zarnowiecki (2020) [30]** | >1 years | 9 (35) | Use ability, appeal and barriers |  |  |
| **Liu (2021) [28]** | 9-18 | 48 (48) | Cultivation of preference |  |  |
|  | 9-18 | 48 (48) | Time and cost |  |  |
