# Supplementary material for: Exploring the values and preferences of children and adolescents with obesity and their parents/caregivers concerning diet or physical activity interventions for weight management: Mega-ethnography of qualitative syntheses
Source: PLoS One. 2026 Jan 20;21(1):e0340875. doi: 10.1371/journal.pone.0340875 (PMC12818672; doi:10.1371/journal.pone.0340875)
Supplement: Attachment — Submitted filename: PONE-D-24-58575_reviewer.RG.pdf [file pone.0340875.s014.pdf]

PLOS ONE

Re: Exploring the values and preferences of children and adolescents with obesity and their parents/caregivers concerning diet or physical activity interventions for weight management: Mega-ethnography of qualitative syntheses of systematic reviews

--Manuscript Draft--

**Manuscript Number:**	PONE-D-24-58575
**Article Type:**	Research Article
**Full Title:**	Re: Exploring the values and preferences of children and adolescents with obesity and their parents/caregivers concerning diet or physical activity interventions for weight management: Mega-ethnography of qualitative syntheses of systematic reviews
**Short Title:**	Views of diet and physical activity interventions for children and adolescents with obesity
**Corresponding Author:**	Munira Essat<br>The University of Sheffield<br>Sheffield, UNITED KINGDOM OF GREAT BRITAIN AND NORTHERN IRELAND
**Keywords:**	Diet; physical activity; exercise; qualitative research; review of reviews; mega-ethnography; Obesity
**Abstract:**	Introduction:<br>Obesity and overweight are a global health problem affecting over 2.6 billion people worldwide. The prevalence of overweight and obesity among children and adolescents aged 5-19 has risen dramatically from just 4% in 1975 to just over 18% in 2016. These worrying trends predict a generation of children and adolescents growing up with obesity and a greater risk of diseases. Diet and physical activity including exercise can play an important role in combatting obesity. The aim of this review of reviews was to explore the values and preferences of children, adolescents, and parents/caregivers that impact on the decision to engage with and/or maintain diet or physical activity interventions for management of obesity.<br>Methods:<br>Eleven electronic databases were searched (from 2010 onwards) to identify reviews that included qualitative research on values and preferences, attitudes or experiences of children or adolescents with obesity, or their caregivers, concerning diet or physical activity interventions. Supplementary grey literature searching, citation searching and examination of reference lists of included reviews was also undertaken. Methodological limitations of included reviews were assessed using the Swedish Agency for Health Technology Assessment (SBU) tool. Synthesis was performed using a mega-ethnography approach.<br>Results:<br>Fourteen reviews or syntheses were included. Most included studies were from high-income countries. Six key factors affect young people or their caregivers when initiating and continuing with physical activity and/or dietary management interventions; perceptions concerning the value of interventions; competing priorities; the role of support; the physical environment; the nature and content of the intervention; and costs.<br>Conclusion:<br>This review identified multifaceted factors that influenced how young people, or their caregivers engage with physical activity and/or dietary management programmes. Interventions are unlikely to achieve their impact in isolation. Young people often need emotional and psychological support within such programmes, alongside diet and physical activity. Our findings support the importance of parenting and parental health concerns in facilitating the development and engagement with weight management programmes, as well as the need for experienced and empathetic health professionals and supportive peers.
**Order of Authors:**	Munira Essat
	Christopher Carroll
	Andrew Booth

Powered by Editorial Manager® and ProduXion Manager® from Aries Systems Corporation

	Joanna Leaviss
	Diana Castelblanco Cuevas
	Roos Verstraeten,
**Additional Information:**	
**Question**	**Response**
**Financial Disclosure**<br><br>Enter a financial disclosure statement that describes the sources of funding for the work included in this submission. Review the submission guidelines for detailed requirements. View published research articles from *PLOS ONE* for specific examples.<br><br>This statement is required for submission and **will appear in the published article** if the submission is accepted. Please make sure it is accurate.<br><br>**Funded studies**<br>Enter a statement with the following details:<br>• Initials of the authors who received each award<br>• Grant numbers awarded to each author<br>• The full name of each funder<br>• URL of each funder website<br>• Did the sponsors or funders play any role in the study design, data collection and analysis, decision to publish, or preparation of the manuscript?<br><br>Did you receive funding for this work?	Yes
Please add funding details.<br>as follow-up to "**Financial Disclosure**<br><br>Enter a financial disclosure statement that describes the sources of funding for the work included in this submission. Review the submission guidelines for detailed requirements. View published research articles from *PLOS ONE* for specific examples.<br><br>This statement is required for submission and **will appear in the published article** if	The Department of Nutrition and Food Safety at the World Health Organization (WHO) commissioned and provided financial support to the University of Sheffield for this work. WHO acknowledges the financial support from the Norwegian Agency for Development Cooperation (NORAD), The Swedish International Development Cooperation Agency (SIDA), The Government of the Grand Duchy of Luxembourg, and the Government of Germany (BMG) to the Department of Nutrition and Food Safety.

Powered by Editorial Manager® and ProduXion Manager® from Aries Systems Corporation

the submission is accepted. Please make sure it is accurate.<br><br>**Funded studies**<br>Enter a statement with the following details:<br>• Initials of the authors who received each award<br>• Grant numbers awarded to each author<br>• The full name of each funder<br>• URL of each funder website<br>• Did the sponsors or funders play any role in the study design, data collection and analysis, decision to publish, or preparation of the manuscript?<br><br>Did you receive funding for this work?"	
Please select the country of your main research funder (please select carefully as in some cases this is used in fee calculation).<br>as follow-up to "**Financial Disclosure**<br><br>Enter a financial disclosure statement that describes the sources of funding for the work included in this submission. Review the submission guidelines for detailed requirements. View published research articles from *PLOS ONE* for specific examples.<br><br>This statement is required for submission and **will appear in the published article** if the submission is accepted. Please make sure it is accurate.<br><br>**Funded studies**<br>Enter a statement with the following details:<br>• Initials of the authors who received each award<br>• Grant numbers awarded to each author<br>• The full name of each funder<br>• URL of each funder website<br>• Did the sponsors or funders play any role in the study design, data collection and analysis, decision to publish, or preparation of the manuscript?	UNITED KINGDOM - GB

Did you receive funding for this work?"	
**Competing Interests**<br><br>Use the instructions below to enter a competing interest statement for this submission. On behalf of all authors, disclose any competing interests that could be perceived to bias this work—acknowledging all financial support and any other relevant financial or non-financial competing interests.<br><br>This statement is required for submission and **will appear in the published article** if the submission is accepted. Please make sure it is accurate and that any funding sources listed in your Funding Information later in the submission form are also declared in your Financial Disclosure statement.<br><br>View published research articles from *PLOS ONE* for specific examples.<br><br>**NO authors have competing interests**<br><br>Enter: *The authors have declared that no competing interests exist.*<br><br>**Authors with competing interests**<br><br>Enter competing interest details beginning with this statement:<br><br>*I have read the journal's policy and the authors of this manuscript have the following competing interests: [insert competing interests here]*<br><br>* typeset	The authors have declared that no competing interests exist
**Ethics Statement**<br><br>Enter an ethics statement for this submission. This statement is required if the study involved:<br><br>• Human participants<br>• Human specimens or tissue	N/A

• Vertebrate animals or cephalopods<br>• Vertebrate embryos or tissues<br>• Field research<br><br>Write "N/A" if the submission does not require an ethics statement.<br><br>General guidance is provided below. Consult the submission guidelines for detailed instructions. **Make sure that all information entered here is included in the Methods section of the manuscript.**	

Powered by Editorial Manager® and ProduXion Manager® from Aries Systems Corporation

**Format for specific study types**<br><br>**Human Subject Research (involving human participants and/or tissue)**<br>• Give the name of the institutional review board or ethics committee that approved the study<br>• Include the approval number and/or a statement indicating approval of this research<br>• Indicate the form of consent obtained (written/oral) or the reason that consent was not obtained (e.g. the data were analyzed anonymously)<br><br>**Animal Research (involving vertebrate animals, embryos or tissues)**<br>• Provide the name of the Institutional Animal Care and Use Committee (IACUC) or other relevant ethics board that reviewed the study protocol, and indicate whether they approved this research or granted a formal waiver of ethical approval<br>• Include an approval number if one was obtained<br>• If the study involved *non-human primates*, add *additional details* about animal welfare and steps taken to ameliorate suffering<br>• If anesthesia, euthanasia, or any kind of animal sacrifice is part of the study, include briefly which substances and/or methods were applied<br><br>**Field Research**<br><br>Include the following details if this study involves the collection of plant, animal, or other materials from a natural setting:<br>• Field permit number<br>• Name of the institution or relevant body that granted permission	
**Data Availability**<br><br>Authors are required to make all data underlying the findings described fully available, without restriction, and from the time of publication. PLOS allows rare exceptions to address legal and ethical concerns. See the PLOS Data Policy and FAQ for detailed information.	Yes - all data are fully available without restriction

A Data Availability Statement describing where the data can be found is required at submission. Your answers to this question constitute the Data Availability Statement and **will be published in the article**, if accepted.<br><br>**Important:** Stating 'data available on request from the author' is not sufficient. If your data are only available upon request, select 'No' for the first question and explain your exceptional situation in the text box.<br><br>Do the authors confirm that all data underlying the findings described in their manuscript are fully available without restriction?	
**Describe where the data may be found in full sentences. If you are copying our sample text, replace any instances of XXX with the appropriate details.**<br><br>• If the data are **held or will be held in a public repository**, include URLs, accession numbers or DOIs. If this information will only be available after acceptance, indicate this by ticking the box below. For example: *All XXX files are available from the XXX database (accession number(s) XXX, XXX.)*.<br>• If the data are all contained **within the manuscript and/or Supporting Information files**, enter the following: *All relevant data are within the manuscript and its Supporting Information files.*<br>• If neither of these applies but you are able to provide **details of access elsewhere**, with or without limitations, please do so. For example:<br><br>*Data cannot be shared publicly because of [XXX]. Data are available from the XXX Institutional Data Access / Ethics Committee (contact via XXX) for researchers who meet the criteria for access to confidential data.*<br><br>*The data underlying the results presented in the study are available from (include the name of the third party*	All relevant data are within the manuscript and its Supporting Information files.

and contact information or URL).<br>• This text is appropriate if the data are owned by a third party and authors do not have permission to share the data.<br><br>* typeset	
Additional data availability information:	Tick here if your circumstances are not covered by the questions above and you need the journal’s help to make your data available.

Powered by Editorial Manager® and ProduXion Manager® from Aries Systems Corporation

Manuscript

Click here to access/download;Manuscript;Essat at al Manuscript.docx

Title: Exploring the values and preferences of children and adolescents with obesity and their parents/caregivers concerning diet or physical activity interventions for weight management: Mega-ethnography of qualitative syntheses of systematic reviews

Authors:

*Munira Essat, Sheffield Centre for Health and Related Research, University of Sheffield, Sheffield, UK

Christopher Carroll, Sheffield Centre for Health and Related Research, University of Sheffield, Sheffield, UK

Andrew Booth, Sheffield Centre for Health and Related Research, University of Sheffield, Sheffield, UK

Joanna Leaviss, Sheffield Centre for Health and Related Research, University of Sheffield, Sheffield, UK

Diana Castelblanco Cuevas, Sheffield Centre for Health and Related Research, University of Sheffield, Sheffield, UK

Roos Verstraeten, European Commission – Joint Research Centre, Ispra, Italy

Running title: Views of diet and physical activity interventions for children and adolescents with obesity

***Corresponding author**: Regent Court, Regent Street, Sheffield, S1 4DA m.essat@sheffield.ac.uk

Abstract

Introduction:

Obesity and overweight are a global health problem affecting over 2.6 billion people worldwide. The prevalence of overweight and obesity among children and adolescents aged 5-19 has risen dramatically from just 4% in 1975 to just over 18% in 2016. These worrying trends predict a generation of children and adolescents growing up with obesity and a greater risk of diseases. Diet and physical activity including exercise can play an important role in combatting obesity. The aim of this review of reviews was to explore the values and preferences of children, adolescents, and parents/caregivers that impact on the decision to engage with and/or maintain diet or physical activity interventions for management of obesity.

Methods:

Eleven electronic databases were searched (from 2010 onwards) to identify reviews that included qualitative research on values and preferences, attitudes or experiences of children or adolescents with obesity, or their caregivers, concerning diet or physical activity interventions. Supplementary grey literature searching, citation searching and examination of reference lists of included reviews was also undertaken. Methodological limitations of included reviews were assessed using the Swedish Agency for Health Technology Assessment (SBU) tool. Synthesis was performed using a mega-ethnography approach.

Results:

Fourteen reviews or syntheses were included. Most included studies were from high-income countries. Six key factors affect young people or their caregivers when initiating and continuing with physical activity and/or dietary management interventions; perceptions concerning the value of interventions; competing priorities; the role of support; the physical environment; the nature and content of the intervention; and costs.

Discussion/Conclusion:

This review identified multifaceted factors that influenced how young people, or their caregivers engage with physical activity and/or dietary management programmes. Interventions are unlikely to

achieve their impact in isolation. Young people often need emotional and psychological support within such programmes, alongside diet and physical activity. Our findings support the importance of parenting and parental health concerns in facilitating the development and engagement with weight management programmes, as well as the need for experienced and empathetic health professionals and supportive peers.

Keywords:

Diet; physical activity; exercise; qualitative research; review of reviews; mega-ethnography; obesity

Introduction

In 2020, it was estimated that 2.6 billion people were overweight and obese (as classified by BMI $\geqslant$ 25kg/m^2) (1, 2). Recent projections estimate that this will increase to over 4 billion, totally over 50% of the global population, by 2035 (1, 2). The prevalence of overweight and obesity among children and adolescents aged 5-19 has risen dramatically from 4% in 1975 to just over 18% in 2016 (340 million) (3); with a projected estimate increase of 10% to 20% for boys and 8% to 18% for girls by 2035 (1, 2). Similarly, rates of overweight and obesity are also rising in children under the age of 5 years, with an estimated 39 million classified as overweight or obese in 2020 (4). Obesity also contributes to multiple comorbid and diet-related chronic medical conditions, including type 2 diabetes and some forms of cancer (5). A failure of prevention, management or treatment interventions could lead to a global economic impact of $4.32 trillion per year by 2035, with nearly 3% of global Gross Domestic Product (GDP) treating illnesses related to obesity (2). No country is on track to halt the rise of obesity and meet the World Health Organization (WHO) target of ‘no increase on 2010 levels by 2025 (1, 2). Similarly, very few countries are on course to meet the targets for diet-related non-communicable diseases, including obesity(1, 2, 6).

To increase efforts to prevent, manage and treat obesity throughout the life course, WHO recognized the need to supplement existing prevention-focused guidelines with normative guidance focusing on people-centred integrated management and care of children and adolescents with obesity. This is to be achieved using a primary health care approach that focuses on services providing health and obesity management advice and guidance for children with obesity and their families. To support these efforts, the WHO commissioned a series of parallel reviews to underpin guidance on this issue. The reviews examined evidence, from children and adolescents with obesity, or their parents and caregivers, concerning diet, physical activity including exercise, behavioural, pharmacological and bariatric surgery or weight loss device interventions for managing obesity. This paper includes the findings from two of these reviews, examining the evidence on dietary interventions and physical activity interventions. Although various reviews have synthesized the evidence on obesity management interventions for both strategies (diet and physical activity), but none have purely focused on values and preferences (7). Therefore, to provide evidence-based guidance for WHO and help inform the

development of future interventions, we report the first review of systematic reviews to explore the values and preferences of children and parents/caregivers concerning diet and physical activity interventions for children and adolescents with obesity with the aim of understanding why children engage (or do not engage) and continue with (or drop out of) such interventions.

Methods

This review of review used the EPOC's Protocol and Review Template for Qualitative Evidence Synthesis to ensure a consistent and coherent approach (8). Pre-specified review protocols were registered with the PROSPERO international prospective register of systematic reviews [diet: CRD42022312881 and physical activity: CRD42022310922].

Eligibility criteria

To be included in this combined intervention review, reviews had to satisfy the topic of interest, defined here using the PerSPECTiF framework (9): see **Table 1**.

Table 1. Problem definition (PerSPECTiF)

**Perspective**	**Setting**	**Phenomenon of Interest**	**Environment**	**Comparison**	**Time/ Timing**	**Findings**
Children with obesity	Home	Diet Intervention Physical Activity Interventions	High, Middle and Low Income Countries	PROGRESS+ Factors	Initiation	Themes
Adolescents with obesity	School				Continuation	Values
Parents and Caregivers of children and adolescents with obesity?	Clinical settings					Preferences
						Attitudes

The term "adolescent" is defined by the World Health Organization (WHO) as a person aged 10–19 years old, and "youth" as one between the age of 15–24 years old.(10)

PROGRESS+ Factors: Place of residence; Race/ethnicity/ culture/ language; Occupation; Gender/sex; Religion; Education; Socioeconomic status; Social capital
Plus refers to:
1) personal characteristics associated with discrimination (e.g., age, disability)
2) features of relationships (e.g., smoking parents, excluded from school
3) time-dependent relationships (e.g., leaving the hospital, respite care, other instances where a person may be temporarily at a disadvantage)

For inclusion reviews had to include at least one primary qualitative study and collect and analyse qualitative data using qualitative data collection and analysis methods. Mixed methods reviews were

included where it was possible to extract qualitative data from studies that had been collected and analysed using qualitative methods. Reviews could be either published or unpublished and not limited by language. We did not apply any 'quality threshold' for inclusion.

Physical activity interventions specifically relate to provision of selected and/or *structured* physical activity options for individuals and groups with obesity. Dietary interventions specifically relate to any intervention for dietary management with the aim of managing obesity, including nutritional options and diet concerns. Studies from January 2010 onwards were included to ensure the most contemporaneous evidence (although reviews published during this period would inevitably capture earlier relevant primary studies).

Reviews were excluded if the health effects of the interventions could not be attributed *distinctly* to diet or physical activity or explored diet or physical activity for obesity *prevention*. Typically, the focus of this review was on qualitative data describing the values and preferences of children and adolescents, and of their parents/caregivers, rather than focusing on a specific type of exercise or physical activity involved or the identifiable composition of the diet. The views of other adults involved in dietary management or physical activities e.g., clinicians, schoolteachers or other staff, although acknowledged as important, were excluded from the scope of the review.

Search methods for identification of studies

The search strategies for each database was based on previous reviews conducted on the topic of obesity (11). The search approach used was documented using the STARLITE elements and presented in **Table 2**. Searches for reviews were conducted in January 2022 and updated in June 2024. A methodological filter was used to populate the Cochrane Qualitative and Implementation Methods Group register of qualitative evidence syntheses. Additional supplementary searches included a grey literature search in the following sources: OpenGrey, Grey Literature Report, Agency for Healthcare Research and Quality, National Institute for Health and Clinical Excellence website and Eldis; citation reference search for all included studies; checking reference lists of reviews from a previous mega-aggregation conducted for the WHO (11); and of all the included reviews and key references. We also actively pursued related studies identified through shared authorship, citation networks or related

articles features (12). The Ovid MEDLINE search strategy, which was adapted for other databases is presented in **Supplementary Appendix 1**.

Table 2. The STARLITE elements

	**What matters to children and adolescents with obesity and their parents and caregivers concerning diet or physical activity interventions for children and adolescents with obesity?**
**Sampling strategy**	Comprehensive: samples from international and regional databases
**Type of study**	Systematic reviews that include qualitative research studies
**Approaches**	Backwards citation chasing (reference checking) and Forwards citation chasing (Google Scholar citation searching using Publish or Perish)
**Range of years**	January 1, 2010 – June 2024
**Limits**	No language limits; Human only
**Inclusions and Exclusions**	Inclusion (Mega-aggregation): Reviews reporting perceptions of children, parents or caregivers concerning intervention for dietary management or physical activity; desires, fears, experiences, coping strategies etc., Reviews exploring initiation, contemplation or continuation of diet or physical activity interventions or services
**Terms used**	See Appendix 1 for full strategy. Conceptualised as Obesity concepts AND Child/Adolescent/Parent terms AND Qualitative Review labels
**Electronic sources**	11 data sources: AJOL; ASSIA; CINAHL (Ovid); EMBASE (Ovid); EPISTEMONIKOS; Google Scholar; LILACS; MEDLINE (Ovid); PsycINFO (Ovid); Scopus; WoS
AJOL, African Journals Online; ASSIA, Applied Social Sciences Index and Abstracts; CINAHL (Ovid), Cumulative Index of Nursing and Allied Health Literature; LILACS, Latin American and Caribbean Health Sciences Literature; WoS, Web of Science.	

Selection of studies

Once acceptable agreement was reached on the eligibility criteria following use of a test set of 100 references, the review team divided the remaining references between themselves for title and abstract screening. A second reviewer checked 10% of the excluded references for each reviewer. Any discrepancies were discussed and resolved within the entire review team. Full text was obtained for all papers identified as potentially relevant. The lead reviewer and an independent additional reviewer then assessed these papers for final inclusion. Disagreements were resolved by discussion or, when required, by involving a third reviewer.

Data extraction

Data was extracted in a pre-piloted data extraction form by one reviewer and checked by a second reviewer (CC or ME). Inconsistencies were resolved by discussion and if necessary, consultation with

a third reviewer (AB). The following data were extracted: author; year; aim of review; type of review; databases searched; inclusion criteria (age groups and regions/countries covered); number of included qualitative and mixed-method studies; appraisal tool used; type of synthesis conducted; potential additional relevant references. The form also extracted synthesis data: relevant themes identified in the review; relevant themes identified by the authors of the primary studies (second order constructs); and any data from the primary studies supporting that theme (first order constructs).

Assessing the methodological limitations of included studies

The methodological limitations of included reviews of qualitative evidence was assessed using the Swedish Agency for Health Technology Assessment (SBU) tool (13) by one reviewer and checked by a second reviewer. Disagreements were resolved by discussion. Data richness is an important consideration within qualitative research. We applied a previously used QES rating scale (14), modified from a scale originally developed for primary qualitative studies (15). This 3-point score system considers the amount and depth of qualitative data and its relevance to the research question. A QES is scored 3 (the highest score of data richness) when it includes large quantities of qualitative studies (>20) or qualitative data (that is, illustrative quotations from primary supporting studies); 2 for a substantive quantity of qualitative studies (10-20) or qualitative data; and 1 (lowest data richness score) if there are few qualitative studies (<10) or very little or no qualitative data.

Data analysis and synthesis

Synthesis was performed using a mega-ethnography approach (16). Mega-ethnography requires the extraction of diverse findings from included reviews: first order constructs (any relevant participant verbatim comments from the original primary research studies); second order constructs (primary research authors' statements of findings); and third order constructs (the review authors' own themes of statements of findings emerging from their synthesis). Constructs were extracted independently by a single reviewer and checked by the second reviewer (CC or ME), inconsistencies were resolved by discussion and if necessary, consultation with a third reviewer (AB). Synthesis involved the

interpretation of the first, second and third order constructs by one reviewer (CC or ME) to develop new, fourth-order constructs. These constructs were grouped into themes and used to develop the review findings.

Review author reflexivity

As a review team, we were very aware of the need to consider our own biases when conducting this work. A full reflexivity statement is available in **Supplementary Appendix 2**.

Results

Results of the search

A total of 3343 titles and abstracts were screened, and 146 papers (including 40 known relevant reviews) were selected for full-paper screening. From these, fourteen reviews and syntheses were identified that satisfied the inclusion criteria (17-30). Of these, seven reviews (19, 21-25, 27) were included in both the dietary management review and physical activity review; four reviews (17, 20, 29, 30) were included in the physical activity review only; and three reviews (18, 26, 28) were included in the dietary management review only. The overall process is detailed in the PRISMA flow diagram **(Figure 1)**. Details of reviews excluded at full text stage with reasons for exclusion are available in **Supplementary Appendix 3.**

[INSERT FIGURE 1 HERE]

Figure 1: PRISMA 2020 flow diagram (31)

Description of the included reviews

Details of the included reviews are presented in **Tables 3 and 4**. The fourteen included reviews or syntheses (17-30) were published in English between 2013 and 2024. One review (19) included only studies of children (up to the age of 11 years); five reviews (17, 20, 21, 26, 30) included only studies of adolescents (aged 9-18 years); one review (18) did not specify the age. The remaining seven reviews (22-25, 27-29) included studies relating to both children and adolescents (from 0/6 to 18 years). Eleven (17, 19, 21-28, 30) of the fourteen reviews included study populations from Europe, eleven (17, 18, 21-28, 30) from North America, and eight (17, 20, 21, 23-26, 28) from the Western Pacific region (Australia and New Zealand), including one review (20) that drew upon evidence exclusively from that region. Only three reviews included primary studies from Africa, (17, 24, 26) and four from south-east Asia (21, 25, 26, 30). One review did not report the origins of the included studies (29). The evidence were predominantly from high-income countries.

The number of qualitative studies in total, reported as included, across the reviews was 227, and the number of qualitative studies included in any review ranged from two (20) to 48 (26). The included reviews had searched between three (18) and 11 (19) bibliographic databases. The majority of reviews conducted thematic synthesis.

Table 3. Review characteristics

First Author (year of publication)	Review type	Age of Children (review inclusion criteria)	Regions	Number of Qualitative studies	Method of Synthesis
**Dietary management and physical activity management**					
Burchett (2018) (19)	Systematic review using QCA	0-11	Europe	11 (11)	Thematic analysis
Jones (2019) (21)	QES	9-18	North America, Europe, South East Asia, Western Pacific	24 (28)	Thematic Synthesis
Kebbe (2017) (22)	Thematic synthesis	2-18	North America, Europe	11 (17)	Thematic synthesis
Kelleher (2017) (23)	Systematic review	2-18	North America, Europe, Western Pacific	6 (13)	Narrative synthesis
Lachal (2013) (24)	QES	0-18	Africa, North America, Central America, Eastern Mediterranean, Europe, Western Pacific	45 (45)	Meta-synthesis, adapted from meta-ethnography
Lang (2021) (25)	QES	2-18	North America, Europe, South East Asia, Western Pacific	16 (16)	Thematic synthesis
Roberts (2021) (27)	Other – Scoping review	2-18	North America, Europe	9 (12)	Summative content analysis (SCA)
**Physical activity management (including exercise) only**					
Chen (2024) (29)	Systematic review	6-18	NR	15 (31)	Thematic synthesis
Haracz (2013) (20)	Integrative review	9-18	Western Pacific	2 (22)	Thematic synthesis
Skogen (2022) (30)	Scoping review	13-18	North America, Europe, South East Asia	6 (12)	Thematic synthesis
Stankov (2012) (17)	QES	9-18	Africa, North America, Europe, Western Pacific	15 (15)	Miles and Huberman's cross-case analysis integrated with thematic network analysis
**Dietary management only**					
Liu (2021) (26)	QES	9-18	Africa, North America, Central America, Eastern Mediterranean, Europe, South East Asia, Western Pacific	48 (48)	Thematic synthesis
Molina (2021) (18)	Scoping review	NR	North America	10 (44)	Narrative synthesis

Zarnowiecki (2020) (28)	Systematic review	>1 years	North America, Europe, Western Pacific	9 (35)	Narrative synthesis

NR: Not reported; QCA: Qualitative Comparative Analysis QES: Qualitative evidence synthesis; SCA: Summative content analysis

Table 4. Review methods

First Author (year of publication)	Search Dates	Total Number of Databases Listed	Main Databases Used	Additional Databases	Supplementary Search (i.e. non-Database) Methods	Quality Assessment Tool(s)	GRADE-CERQual Used
**Dietary management and physical activity management**							
Burchett (2018) (19)	1990 – March 2016	11	MEDLINE, CINAHL, PsycINFO, ASSIA, Web of Knowledge / Science Citation Index / Social Science Citation Index, British Education Index, Education Resources Information Centre (ERIC), Index to Theses in Great Britain and Ireland, HMIC, PubMed, Social Policy and Practice	HMIC, PubMed, Social Policy and Practice	Studies from existing reviews with update search in December 2015 - details not reported	Other	No
Jones, (2019) (21)	Database inception to July 2018	6	MEDLINE, CINAHL, EMBASE, PsycINFO, ASSIA, Web of Knowledge / Science Citation Index/ Social Science Citation Index	None	Relevant systematic reviews, key journals and reference lists of included studies were manually screened. A specialist librarian was consulted to refine the search.	EPPI-Centre Tool	No
Kebbe (2017) (22)	1980–June 2016	6	CINAHL, EMBASE, MEDLINE, PsycINFO, ProQuest Dissertations and Theses and Scopus)	None	RLIS	MMAT	No

Kelleher, (2017) (23)	Database inception to 2015	4	MEDLINE, CINAHL, EMBASE, PsycINFO	None	RLIS	Other - Bowling's quality checklist	No
Lachal (2013) (24)	1990 – 2011	5	MEDLINE, CINAHL, EMBASE, PsycINFO, Scopus	None	RLIS	Other	No
Lang (2021) (25)	Database inception to January 2019; January 2019 to April 2020	6	MEDLINE In-Process and other nonindexed citations, EMBASE, CINAHL Plus, PsycINFO, Ovid Emcare, and Scopus	None	RLIS	CASP	No
Roberts (2021) (27)	2006 - February 2018	6	PubMed, CINAHL, Scopus, PsycINFO, Cochrane Reviews, and Embase	None	An ancestral search of reference lists from seminal papers	None	No
**Physical activity management (including exercise) only**							
Chen (2024) (29)	2003 - 2023	3	Web of Science, Scopus, and PubMed	None	None	CASP	No
Haracz (2013) (20)	2002-2012	4	CINAHL, Medline, AMED and PsycINFO,	None	Searches of nine occupational therapy journals	McMaster University Guidelines and Appraisal Forms for Quantitative and Qualitative Research	No
Skogen 2022 (30)	Inception to 2020	4	PubMed, Web of Science, SportDiscuss, and Cinahl		Reference list of all included studies and reviews found through database searches	None	No
Stankov (2012) (17)	1950 – 2009	6	MEDLINE, CINAHL, EMBASE, PsycINFO, SportsDiscus and Academic Search Premier	None	Searches of non-peer reviewed 'grey' literature (i.e. Google Scholar, World Wide Web and professional networks) and pearling reference lists	CASP	No
**Dietary management only**							
Liu (2021) (26)	Database inception to 31 July 2020	4	PubMed, Web of Science, PsycINFO, and EMBASE	None	RLIS	Adapted CASP	No

Molina (2021) (18)	Database inception to February to April 2020.	3	PubMed, LILACS and EconLit	Governmental websites of each of the 19 countries (e.g., Ministry of Health), Governmental press releases or other official documents. websites of WHO, PAHO and the World Cancer Research<br><br>Fund International (WCRFI) Google Scholar	Reference list of all retrieved documents or publications	None	No
Zarnowiecki (2020) (28)	1946 to October 22, 2018 restricted 2013 to 2018	5	MEDLINE (Ovid capturing PubMed) and translated for use in EMCARE (Ovid), PsychINFO (Ovid), Scopus and Pro-Quest databases.	None	RLIS and relevant reviews identified in the search	None	No

CASP: Critical Appraisal Skills Programme; MMAT: Mixed Methods Assessment Tool; NR: Not Reported; RLIS: Reference Lists of Included Studies

Methodological limitations of the studies

Details of the quality and richness assessments are presented in **Table 5**. The methodological assessment judged six of the reviews to have been conducted with only a minor risk to rigour (17, 21-25); for six reviews the risk to rigour was deemed to be moderate (18-20, 26, 29, 30); and for two reviews the risk to rigour was assessed as high (27, 28). Three reviews were considered to be supported by rich data (21, 24, 26), two of which were also assessed as being conducted with only a minor risk to rigour (21, 24) and four were deemed to be supported by moderately rich data (17, 19, 25, 29). The data were assessed as meagre in the remaining seven reviews (18, 20, 22, 23, 27, 28, 30).

Table 5. Quality Assessment (risk to rigour evaluated using the SBU tool) and Richness Assessment

	Burchett 2018 (19)	Chen 2024(29)	Jones 2019 (21)	Kebbe 2017 (22)	Kelleher 2017 (23)	Lachal 2013 (24)	Lang 2021 (25)	Roberts 2021 (27)	Haracz 2013 (20)	Stankov 2014 (17)	Skogen 2022(30)	Liu 2021 (26)	Molina 2021 (18)	Zarnowiecki 2020 (28)
1. Aim	L	M	L	L	L	L	M	L	M	L	L	L	L	L
2. Search approach	M	L	L	L	L	L	L	L	L	L	L	L	L	L
3. Inclusion criteria	L	L	L	L	L	L	L	L	L	L	L	L	M	L
4. Competence	M	M	M	L	M	M	L	H	L	M	L	M	M	H
5. Search strategy	M	L	L	L	L	L	M	M	M	L	L	M	H	L
6. Study screening	L	L	L	L	M	L	L	H	L	M	L	L	M	L
7. Appraisal tool	H	L	L	M	L	L	L	H	L	L	H	M	H	M
8. Appraisal process	H	L	L	L	L	M	L	H	H	L	H	H	M	L
9. Synthesis (method appropriateness)	L	L	L	L	L	L	H	M	L	L	M	L	L	M
10. Synthesis process	L	L	L	L	M	L	H	M	M	L	M	L	L	M
11. Synthesis output: clearly grounded	L	L	L	L	L	L	H	L	H	L	L	L	L	H

in primary studies														
12. Synthesis output: beyond a summary of primary studies	H	M	M	L	L	M	L	L	M	L	M	L	H	H
13. Confidence in finding (CERQual)	H	H	L	H	H	H	H	H	H	H	H	H	H	H
Overall verdict (concerns)	Mod-erate	Moderate	Minor	Minor	Minor	Minor	Minor	High	Mod-erate	Minor	Moderate	Moderate	Moderate	High
Data richness score	2	2	3	1	1	3	2	1	1	2	1	3	1	1

H: High risk to rigour. M: Moderate risk to rigour. L: Low risk to rigour

Data richness: 3=rich data; 2=moderately rich data; 1=meagre data

Review findings

A summary of the review findings (the fourth-order constructs), and a list of the supporting reviews for each finding, is presented in **Table 6**. Full details of the findings of the synthesis: illustrative text extracts, the novel, fourth-order constructs, their basis in the third-order constructs of the included reviews, and the details of the reviews underpinning each finding, are presented in the **Supplementary Appendix 4 Tables S1-S6.** A narrative detailing each of the findings is presented below.

Table 6. Overall summary of qualitative findings (diet and physical activity interventions)

Supporting studies	Findings
**Perceptions of the value (for full details see Table S1)**	
Chen (2024) (29) Jones (2019) (21) Kebbe (2017) (22) Kelleher (2017) (23) Lachal (2013) (24) Lang (2021) (25) Liu (2021) (26) Roberts (2021) (27) Skogen (2022) (17) (n=9)	**Children and adolescents can be motivated to engage with obesity health services that focus on diet or physical activity interventions because of the perceived benefits of such activities**<br>— Benefits: weight loss<br>— Benefits: more than just weight loss<br>— Benefits: can differ by gender<br>— Motivations: Observing success in others
Chen (2024) (29) Kebbe (2017) (22) Lachal (2013) (24) Lang (2021) (25) Stankov (2012 (17) Skogen (2022) (17) (n=6)	**Children and adolescents can view the idea of diet or physical activity interventions negatively, and question the value of such activities**<br>— Value of intervention questioned
**Competing priorities (for full details see Table S2)**	
Kebbe (2017) (22) Lang (2021) (25) Liu (2021) (26) Roberts (2021) (27) Stankov (2012) (17) (n=5)	**Individual children, adolescents and families have many commitments, and competing priorities can represent a problem when it comes to engaging in dietary or physical activity interventions**<br>— Opportunities: Absence of structured activities<br>— Opportunities: Insufficient time (for exercise or time to prepare meals, engage and implement intervention, encourage, and discuss healthy diet)
**The role of support (for full details see Table S3)**	
Chen (2024) (29) Burchett (2018) (19) Jones (2019) (21) Kebbe (2017) (22) Kelleher (2017) (23) Lachal (2013) (24) Lang (2021) (25) Liu (2021) (26) Roberts (2021) (27) Stankov (2012) (17)	**Family involvement and support is important if children and adolescents are to engage with dietary or physical activity interventions**<br>— Concerns over parents' and siblings' levels of engagement with the intervention<br>— Preferences for high levels of engagement with the intervention by parents and siblings<br>— Competing priorities<br>— Parents need to act as good role models by participating in exercise and physical activity

Skogen (2022) (17) (n=11)	— Reliance on parents to provide access to and means of doing adequate physical activity<br>— Reliance on parents for resource to access and to provide means of healthy food including purchasing and preparing healthy meals<br>— Barriers: Challenging family dynamics – conflicting opinions and approaches to lifestyle change
Chen (2024) (29) Jones (2019) (21) Kebbe (2017) (22) Lachal (2013) (24) Lang (2021) (25) Roberts (2021) (27) Stankov (2012) (17) Zarnowiecki (2020) (28) (n=8)	**Health worker support is important if children and adolescents are to engage with dietary or physical activity interventions**<br>— Health workers need to provide supportive, structured guidance<br>— Advice and support from health workers is valued<br>— Concerns over health worker levels of engagement with the intervention<br>— Health workers need to provide optimal care, be knowledgeable, share information and be empathetic<br>— Barrier: Negative experience with health workers and lack of support<br>— Concerns over stigmatization
Chen (2024) (29) Burchett (2018) (19) Jones (2019) (21) Kebbe (2017) (22) Kelleher (2017) (23) Lang (2021) (25) Roberts (2021) (27) Stankov (2012) (17) Skogen (2022) (17) (n=9)	**Peer involvement and support is important if children and adolescents are to engage with dietary or physical activity interventions**<br>— Friends and peers need to act as good role models by participating in exercise and physical activity<br>— Friends and peers need to offer support<br>— Friends and peers Involvement and engagement with the intervention makes individual feel a sense of belonging and being accepted<br>— Barrier: Peer pressure, influence of bad behaviour from peers
**The environment (for full details see Table S4)**	
Chen (2024) (29) Jones (2019) (21) Lang (2021) (25) Stankov (2012) (17) (n=4)	**Physical resources at school (spaces, equipment and facilities) need to be accessible and appropriate if children and adolescents are to engage with physical activity interventions**<br>— Appropriate changing and exercise facilities need to be accessible at time of intervention and beyond (school)
Chen (2024) (29) Kebbe (2017) (22) Lang (2021) (25) (n=3)	**Physical resources at home and in the community (safe spaces to play and exercise) need to be accessible and appropriate if children and adolescents are to engage with physical activity interventions**<br>— Appropriate exercise facilities need to be accessible at time of intervention and beyond (home and community)
Kebbe (2017) (22) Stankov (2012) (17) (n=2)	**If the weather conditions are inappropriate to the type of planned physical activity, then children and adolescents may not be motivated to engage in physical activity**<br>— Opportunities: weather can limit scope for exercise and physical activity
Kelleher (2017) (23) Lang (2021) (25) Liu (2021) (26) Molina (2021) (18) Roberts (2021) (27) Zarnowiecki (2020) (28) (n=6)	**Resource, programme and facilities at school, home and in the community need to be accessible for children, adolescents and their families to engage with dietary interventions**<br>— Accessibility to programmes and facilities: Clinic Location<br>— Accessibility to programme and facilities: Timing and frequency of appointments<br>— Accessibility to programme and facilities: Logistic and transport<br>— Availability and access to healthy food: Options at school<br>— Availability and access to healthy food: Social and cultural environment<br>— Resource limitations: lack of internet, difficulty in using app, set-up time
**The nature of the diet or physical activity interventions (for full details see Table S5)**	

Burchett (2018) (19) Jones (2019) (21) Kebbe (2017) (22) Kelleher (2017) (23) Lachal (2013) (24) Lang (2021) (25) Liu (2021) (26) Molina (2021) (18) Roberts (2021) (27) Stankov (2012) (17) Zarnowiecki (2020) (28) (n=11)	**The types of diet or physical activity on offer is important; they should be social, fun, interactive, informal and regular; healthy food should be palatable**<br>Characteristics of intervention:<br>— Informal and fun preferred over highly structured or competitive; preferences for social / team / group activities with peers and friends<br>— Preferences for exercise and activity rather than diet as a means of weight management<br>— Preferences for regular or frequent exercise or physical activity<br>— Preferences for group activities and programmes with peers and friends<br>— Preferences for Lifestyle focused intervention, which incorporated nutrition, physical activity and behavioural components.<br>— Intervention should be structured with meal plans and recipe ideas<br>— If intervention includes digital tools, it should be easy to use.<br>— Healthy food should be palatable<br>— Intervention should also include psychological support
Jones (2019) (21) Lachal (2013) (24) Lang (2021) (25) Roberts (2021) (27) Stankov (2012) (17) Zarnowiecki (2020) (28) (n=6)	**The types of diet or physical activity on offer should be appropriate to the child in terms of their age, gender, ethnicity, culture and physical capabilities**<br>Characteristics of intervention<br>— Concerns over required clothing for activities<br>— Perceived as too demanding by professionals and the children and adolescents themselves<br>— Characteristics of intervention: Perceived as too difficult to do at home<br>— Intervention need be tailored and personalised e.g., portion size, recipes, age appropriate<br>— Dietary strategies need to be suited to the child/ adolescent and to their expectations
Burchett (2018) (19) Jones (2019) (21) Kebbe (2017) (22) Kelleher (2017) (23) Lang (2021) (25) Skogen (2022) (17) Stankov (2012) (17) (n=7)	**Children and adolescents feel that it is important to 'fit in' and not to 'stand out'**<br>Characteristics of intervention<br>— Positive experience of being with other children who are the same (normalization)<br>— Social interaction with peers in group session makes the child feel accepted and a sense of belonging<br>— Perceived as stigmatizing, self-conscious<br>— Characteristics of intervention: adopting healthy eating practices with others, such as helping with portion control and sharing food
Burchett (2018) (19)<br>Jones (2019) (21)<br>Haracz (2013) (20)<br>Kebbe (2017) (22)<br>Kelleher (2017) (23)<br>Lang (2021) (25)<br>Roberts (2021) (27)<br>Stankov (2012) (17)<br>Zarnowiecki (2020) (28)<br>(n=9)	**The amount and type of advice on diet or physical activity interventions given to children and families is important (what activities might be best, how to do them, goals to aim for)**<br>Characteristics of intervention:<br>— Preferences for the involvement of specialist<br>— Preference for evidence based, trusted and endorsed information<br>— Use of positive language<br>— Needs to give participants new knowledge and skills<br>— Preferences for autonomy in choice of activities rather than the involvement of specialist providers during and after intervention<br>— Practical sessions preferred to theory<br>— Active engagement<br>— Needs to give participants new knowledge and skills (understanding the nutritional content of different foods and drinks as well as giving them a better awareness of what foods should be eaten in moderation)<br>— Prescriptive and regulated diet routine set by a health worker<br>— Use of concise, clear, consistent, direct and practical messages/advice<br>Need for emotional and knowledge support
**Cost (for full details see Table S6)**	

Kebbe (2017) (22) Lang (2021) (25) Liu (2021) (26) Roberts (2021) (27) Zarnowiecki (2020) (28) (n=5)	**Cost can be a real concern for children and their families when seeking to engage with dietary or physical activity interventions**<br>— Concerns over costs and logistics of attendance<br>— Concerns over cost of healthy food (believes healthy food is more expensive)<br>— Concern over cost of tools and apps to engage with intervention

Figure 2 illustrates the factors that influence whether children and adolescents with obesity, or their caregivers, are willing or able to engage or continue participating over time in physical activity programs or dietary interventions.

[INSERT FIGURE 2 HERE]

Figure 2. Factors affecting willingness or ability to engage with diet, physical activity and exercise interventions

Interventions to manage weight loss based on the views and experiences of children and adolescents with obesity and their caregivers.

Perceptions of the value of interventions

Children and adolescents with obesity can be motivated to engage with obesity health services that focus on diet or physical activity interventions because of the perceived benefits of such activities. Children and adolescents with obesity reported being motivated by several factors to engage with the interventions: not only the expected and hoped-for benefits of weight loss (25, 30), but also other benefits, such as to prevent health sequelae, to be socially accepted, and to build self confidence and self-esteem (21, 23, 25, 26, 29, 30) Adolescents highlighted that their desire for weight loss was partly driven by wanting to take responsibility / have agency for their condition and to follow a healthy lifestyle (21). Some children and adolescents also reported being motivated by observing the achievements of other children and adolescents who have engaged, or are engaging, with such activities (25), as well as recognising their own success (21, 29) Other factors that particularly

motivated adolescents to engage with physical activity include being able to attend a gym (21, 22). Benefits differ by gender, with girls hoping for improvements in weight loss and physical appearance, while boys wanted to build muscle and become good at sport (24).

This finding is based on nine reviews (21-27, 29, 30) (with 180 included primary qualitative studies). Most of the studies had minor or moderate concerns regarding methodological limitations but the data richness score varied amongst the studies.

Children and adolescents with obesity can view the idea of diet or physical activity interventions negatively, and question the value of such activities

Some children and adolescents with obesity viewed the idea of dietary intervention, exercise or physical activity interventions negatively and questioned the value of such activities. However, reasons for questioning the value of these interventions differed between dietary interventions and physical activity interventions. Some children and adolescents with obesity did not engage in physical activity as they gained no pleasure from exercise (22); while some reported that even the idea of such activities occasioned a negative self-image because they were worried about not being able to do the activities due to their weight or a lack of the necessary skills (22) (24, 29, 30).
Where the impact of dietary interventions was small and unfavourable with intangible benefit, this reduced adolescents and children's desire to sustain dietary change (24, 25).

Some participants reported difficulties in engaging with the intervention during periods of increased stress, the problems of coping with hunger, and a dislike of the lifestyle changes required to maintain weight (25).

This finding is based on six reviews (17, 22, 24, 25, 29, 30) (with 108 included primary qualitative studies), with minor or moderate concerns regarding methodological limitations, but variable richness data.

Competing priorities

Individual children, adolescents and families have many commitments; such competing priorities can represent a problem when it comes to thinking about or engaging in diet and physical activity interventions

Children and adolescents with obesity reported that they had few opportunities for structured activity when engaging with exercise and physical activities (25) (22), in part because as they became older, they became more autonomous and did less with family and at school; they increasingly just engaged in sedentary activities (25). They also described having other commitments, such as school work and seeing friends (22) or just lacked the time and energy for such activities (25). Other family members also reported competing priorities of work and family, which reduced the time available for such activities (22). There was also a reported gender and cultural element: African-American adolescent girls reported that physical activity were simply not seen as a priority (17).

In terms of implementing dietary interventions, parents of children and adolescents with obesity reported that due to their multiple commitments, busy schedule and lack of time, it was difficult to make time for discussions about health and diet with their children (25, 27), to implement healthy eating habits (26) or to prepare meals in accordance with the intervention (22, 26).

This finding is based on five reviews (17, 22, 25-27) (with 99 included primary qualitative studies). The methodological quality and the richness of the data varied amongst the studies.

The role of support

Family involvement and support is important if children and adolescents with obesity are to engage with diet or physical activity interventions.

Children and adolescents with obesity reported that they liked parents and family to be involved as much as possible in their dietary management and physical activities; having family actively involved with the intervention (exercise or diet) acted as a motivator to engage and continue engagement with such interventions (22, 23, 29).

Parents also reported valuing such activities (23). Some children and adolescents - of both genders - reported that they valued the support and involvement of mothers the most. The types of support

adolescents preferred from their families differed. Some adolescents with obesity reported that they preferred emotional, motivational or verbal support from family members, including parents and siblings (22, 26, 30). Others preferred active participation in the intervention by family members (17, 22, 24,26). Children and adolescents reported that the failure of parents and siblings to engage with the intervention, or their apparent lack of interest, understanding or knowledge about the need and value of such activities adversely affected whether they were likely to participate (29). This could create a sense of self-blaming (17, 22). Some parents had cultural beliefs suggesting that thinness is a sign of sickness and convinced adolescents that body weight is not an important negative indicator of health (26). The conflicting opinions and approaches to healthy lifestyle from parents and other family members could also be confusing for children and adolescents, preventing them from engaging successfully with the intervention (27).

Some children and adolescents also reported that parents were important role models: adolescents considered it frustrating when parents failed to model appropriate behaviours (17, 22). Some adolescents also reported that personal norms, such as family members themselves living with their own obesity, made it more difficult for them to engage with the intervention (17). Conversely, it was reported that if a family had an active lifestyle, then this made participation with the intervention much easier (23).

Some children and adolescents reported how they depended on their parents either for access to, or to provide the means for participating in, certain exercise and physical activity interventions, such as providing transport to gyms (22, 25). Children and adolescents may also rely on their parents to purchase, prepare and provide intervention-specific food choices (22, 26). Hence, some adolescents described a lack of control and influence over their diet, while others reported that being away from the home and their parents made it difficult for them to maintain healthy lifestyle (22).

This finding is based on eleven reviews (17, 19, 21-27, 29, 30) (with 206 included primary qualitative studies). Most of the studies had minor or moderate concerns regarding methodological limitations, but concerns regarding richness varied.

Health worker support is important if children and adolescents are to engage with diet or physical activity interventions.

Children and adolescents with obesity reported that the failure of health workers to act as role models (24), or to support children and adolescents with structured advice on diet, nutrition, exercise and physical activities, adversely affected their participation in interventions (22, 29). Conversely, sensitive and supportive health workers offered motivation for some children and adolescents (25). Adolescents and their parents valued health workers who were experienced, knowledgeable and who specialised in supporting children with obesity (21). Parents respond best to health workers who are empathetic, direct and clear (24). Parents viewed good staff-participant relationships as vital for continued attendance voicing their intention to drop out of treatment if staff lack experience, enthusiasm or group management skills. Some adolescents also reported that education and physical exercise professionals, rather than supporting those with obesity, contributed to stigmatization, for example, by punishing the whole class for the slowest person or highlighting their weight as an issue (17).

This finding is based on eight reviews (17, 21, 22, 24, 25, 27-29) (with 144 included primary qualitative studies). Most of the studies had minor concerns regarding methodological limitations, but variable concerns regarding richness.

Peer involvement and support is important if children and adolescents are to engage with diet or physical activity interventions.

Children and adolescents with obesity reported that support and reaction from friends and peers was highly influential when deciding to engage with, or reject, dietary management or physical activity interventions. Adolescents in one study described being around their peers as a security blanket, allowing them to feel comfortable and confident (21). Having peer support gave them a sense of belonging by allowing them to talk to others in a similar situation to them, sharing their struggles and issues and this motivated them to take part in the interventions (21, 30). In particular, group sessions were described as having a positive impact on children's confidence and viewed as fundamental to both initiation and maintenance of health behaviour changes (21, 23, 27). Adolescents also reported

that peers needed to act as role models (17). Conversely, the failure of peers to support them in their engagement with healthy diet or physical activity adversely affected the likelihood of their participation with interventions (17, 22, 29, 30) The personal norms of peers could even contribute to the stigmatizing of exercise and physical activities (22). Concerns about stigmatization by peers – either due to the physical activities involved or clothing required, especially for girls – was reported by adolescents as a barrier to engaging with such activities (17, 29).

This finding is based on nine reviews (17, 19, 21-23, 25, 27, 29, 30) (with 113 included primary qualitative studies). Most studies had minor or moderate concerns regarding methodological limitations, but concerns regarding richness varied.

The environment

Physical resources at school (spaces, equipment and facilities) need to be accessible and appropriate if children and adolescents are to engage with physical activity interventions.

Adolescents with obesity reported that they needed their schools to have accessible and appropriate facilities, both during and after school, if they were to be able to engage with physical activity interventions, and to maintain exercise activities after the programme has ended (17, 21, 25, 29).

Some adolescent girls with obesity also pointed out that provision of appropriate facilities includes changing room and exercise areas where they do not feel 'on display' (17, 29). The non-availability of preferred activities was also reported by some adolescents to be an issue: caps on numbers and certain activities only being offered at particular times of the year prevented engagement in preferred exercise interventions (17).

This finding is based on four reviews (17, 21, 25, 29) (with 70 included primary qualitative studies). There were minor to moderate concerns regarding methodological limitations and minor to moderate concerns regarding richness.

Physical resources at home and in the community (safe spaces to play and exercise) need to be accessible and appropriate if children and adolescents are to engage with physical activity interventions.

Some children and adolescents with obesity reported that they wanted access to physical spaces and facilities both at home (22) and in their community (22, 25) to support their engagement with exercise or physical activity interventions, and that such physical spaces should be local and safe (22, 29).

This finding is based on three reviews (22, 25, 29) (with 42 included primary qualitative studies). There were minor to moderate concerns regarding methodological limitations but moderate to serious concerns about richness.

If the weather conditions are inappropriate for the type of planned physical activity, then children and adolescents may not be motivated to engage in physical activity.

Children and adolescents with obesity report concerns about how excessively hot or cold weather limits their opportunities to engage with exercise or physical activity interventions (17, 22).

This finding is based on two reviews (17, 22) (with 26 included primary qualitative studies); there were minor concerns regarding methodological limitations, but serious concerns about richness.

Resource, programme and facilities at school, home and in the community need to be accessible for children, adolescents and their families to engage with dietary interventions.

Adolescents with obesity reported that limited access to intervention-specific food options, as well as facilities that promote healthy eating within school and within the broader social and cultural environment (18, 25), prevented them from engaging with dietary management interventions (26). By contrast, easy access to restaurants and food outlets attracted families to consume ‘unhealthy’ food (26). Moreover clinic hours and scheduling conflicts such as school holidays and after-school activities, clinic location and a lack of transport to programme location and length of visit were reported reasons for families not engaging with the dietary intervention programmes (23, 27, 28).

This finding is based on six reviews (18, 23, 25-28) (with 98 included primary qualitative studies). The methodological quality and the richness of the data varied between the reviews.

The nature of the diet or physical activity intervention

The types of diet or physical activity on offer is important; they should be social, fun, informal and regular.

Parents preferred to enrol their children in programmes that focused on lifestyle (i.e., incorporated nutrition, physical activity and behavioural components). They favoured programmes that took a holistic approach to weight management rather than those that focused on weight loss or dieting alone (23). Adolescents expressed preference for group/team activities and programmes with peers and friends. Some adolescents rued the absence of support from peers when exercising (17), although some adolescents also reported that going to the gym by themselves was best (21). They wanted exercise interventions to be available regularly and frequently, rather than being accessible only intermittently (25) and expressed a greater preference for physical activity interventions over dietary interventions (24).

Children, adolescents and their caregivers also reported that they preferred obesity interventions that were highly practical (19), but also informal and fun (19, 21, 22, 24). They did not like exercise and physical activities that were highly structured or competitive (24). Practical and interactive healthy eating sessions were highly valued such as cooking and using visual approaches e.g., to illustrate portion size (19). Parents and adolescents wanted interventions to be structured with meal plans and recipe ideas (22, 27) and wanted healthy food that tasted good (18, 22).

Overall, obesity management interventions should factor in longer term support that considers the mental health of adolescents and psychological support, as obesity management brings about feelings of failure, guilt and shame (21, 27).

This finding is based on eleven reviews (17-19, 21-28) (with 204 included primary qualitative studies). Concerns over the methodological limitations of the studies and richness of the data varied between the studies.

The types of diet or physical activity on offer should be appropriate to the child in terms of their age, gender, ethnicity, and physical capabilities.

Some children and adolescents with obesity and their parents reported that they wanted weight management programmes to be tailored or personalised (28). The programme should be appropriate for the child/adolescent's age, ability, culture and ethnicity (21, 27). Adolescents expressed strong feelings about the lack of services aimed at them (21). They wanted to engage with obesity interventions that were created with their age group in mind, with strategies that matched their needs and expectations (27).

Some children and adolescents with obesity reported that they found some exercises too demanding, struggling with them on account of their weight or lack of necessary skills (17, 24). The exercises left some adolescents in pain or discomfort, or too tired to do anything else (17). As a result, some adolescents reported a history of 'failure', and that they felt frequently judged when in classes (17). These problems adversely affected children and adolescent engagement and participation. Some adolescents also reported that the required exercises or activities were too difficult to do at home (17).

Dietary management interventions should also take into account parental ability to accommodate recommendations; and give detailed plans to follow – parents noted that they wanted specific and relevant content, such as portion sizes for different ages and appropriate recipes that would be suitable for all family members (27).

This finding is based on six reviews (17, 21, 24, 25, 27, 28) (with 118 included primary qualitative studies). Concerns over the methodological limitations of the studies and richness of the data varied between the studies.

Children and adolescents report that it is important not to 'stand out'.

Some children and adolescents with obesity and their parents report that engaging in physical activities and dietary programmes can be a positive experience if the group is restricted to children and adolescents with obesity, as this offers a normalizing environment where they feel socially accepted (19, 23, 25). Some parents who participated in this study felt it was good to attend and '*speak to other*

parents who are trying to change things', while their children '*could make friends with other kids'* who could '*play on the same level*' as their own child (23). Conversely, standing-out in non-intervention activities was reported by some adolescents to be a highly stigmatizing experience, contributing to negative self-image, especially for girls (17, 30).

This finding is based on seven reviews (17, 19, 21-23, 25, 30) (with 837 included primary qualitative studies); there were minor-to-moderate concerns regarding methodological limitations but mixed concerns regarding richness.

The amount and nature of the advice on diet or physical activity interventions given to children and families is important (what activities might be best, how to do them, goals to aim for)

Some children and adolescents with obesity, and their caregivers, report that the interventions need to give them new skills and knowledge, and practical instruction was much preferred to theory (19, 22, 23). They liked gaining evidence-based knowledge (21, 23). Some adolescents report that they liked specialist providers, who could give them structured guidance, including clear advice on goal-setting, structured meal plans, recipes, quantity and quality of foods (21, 22, 25, 27, 28); and they wanted to know exactly what physical activities were best (17, 20-22). In addition, a non-forceful approach from health workers was appreciated; and they placed value on gentle encouragement and receiving support that focused on more than just weight loss, such as self-esteem and well-being (21).

However, some children and adolescents also report that they prefer a degree of autonomy in their choice of activities, want to set their own targets during interventions (21, 27), rejecting the need for maintenance of support after the intervention had ended (21).

This finding is based on nine reviews (17, 19-23, 25, 27, 28) (with 103 included primary qualitative studies); there were mixed concerns regarding methodological limitations and richness.

Costs

Cost can be a real concern for children and their families when seeking to engage with dietary interventions or with physical activity interventions.

Concerns were expressed over costs: not just attending clinics but also accessing necessary resources (27). Some parents reported that the need to pay for services, or a lack of insurance coverage, made it difficult to engage with the intervention (27). Other parents were concerned at the cost of using digital tools and other resources (28). Some parents and adolescents reported that limited finances made it difficult to eat intervention-specific foods or engage in certain exercise and physical activities (22, 26).

This finding is based on five reviews (22, 25-28) (with 93 included primary qualitative studies); there were mix concerns regarding methodological limitations and richness.

Discussion

This review of qualitative evidence syntheses (mega-ethnography) explored the perspectives of children and adolescents with obesity, as well as their parents and caregivers, regarding the idea and experience of physical activity interventions and/or dietary intervention to manage obesity. By synthesising the evidence from all relevant reviews and their included primary qualitative studies, the current review creates a broader, deeper overview of the evidence base on this topic to inform evidence-supported guidance. Several factors were identified that affect whether children and adolescents with obesity are prepared to consider, participate in and continue with physical activity interventions and/or dietary interventions to manage obesity. Children and adolescents are not always convinced of the value of dietary and or physical interventions. Where they and their family are knowledgeable and/or can see ready benefits from engagement they may be in favour of engaging with the intervention programmes. However, the interventions must contend with many competing priorities both for the young people and their families. This helps to explain why family involvement and support are crucial if children and adolescents are to engage in dietary and physical interventions. Involvement and support from health workers and other support is key in encouraging engagement with dietary and/or physical interventions and this needs to be enhanced by involvement and support from peers to help the child or adolescent to fit in and to feel accepted. It is not just enough to navigate the young person through the pathway of the intervention, but resources and support needs to be

available post-intervention in the form of long-term and ongoing support. Health workers need to be able to tailor the amount and type of advice to the needs of the young person and their family.

Difficulties in offering physical and/or dietary management interventions relate to their cost. Such as engaging in certain physical activities and healthy foods are not always able to compete on price with less-healthy foods. Equity considerations are therefore important, particularly for low-income families. Low-income families may also face challenges accessing resources, facilities and the programmes themselves. Ongoing attendance may be supported by flexible work schedules, choice of appointment times, adequate family financial resources, and children's motivation (32).

Findings specific to dietary management interventions suggest several key areas of focus. Children and adolescents and their parents or caregivers wanted to be equipped with the necessary skills to prepare tasty, healthy, and low-cost meals. In addition, they valued the intervention to be interactive, social, fun, structured and appropriate to the young person’s age, ethnicity, and culture. These practical skills related to food preparation help motivate individuals to make more health-conscious food choices and prioritize nutrition and make healthy eating more accessible and enjoyable.

Findings specific to physical activity interventions highlight the importance of non-competitive activities to ensure inclusivity and reduce pressure, particularly for young people who may feel discouraged by competition. Additionally, the need for adequate resources is emphasized, including safe spaces and equipment for physical activities. These resources should be accessible both in the community (where children live) and in schools. Furthermore, weather conditions also play a crucial role in ensuring that outdoor activities can be consistently accessible, suggesting the importance of planning for both indoor and outdoor options.

Reviews included in this synthesis confirm that adolescents highly value active engagement when learning about healthy eating and physical activities; a finding also found in younger children, who prefer practical and interactive experiences, rather than receiving didactic information(19, 21). Other reviews confirm the pivotal role that parents play, not only in the provision of food in the household and participation in physical activity but also in parent modelling of healthy lifestyles influence their

children's choices (33). The value of paediatric weight management programmes that seek to educate the whole family is corroborated by many studies (34). Research findings again suggest that young people and their parents alike prefer services that provide a patient-and-family centred approach, provided that it is also tailored through the use of individualized information and resources. Given that children who experience obesity in everyday life often face stigmatisation or even bullying (21, 24, 30, 35), it is understandable why group support from peers is perceived as critical in obesity management (19). However, it is important to recognise that social comparison, a recognised feature of group approaches, may also have unintended effects when a child perceives themselves to be different from the group (35). Another important finding, that corresponds with findings from health promotion to young people (36), is that improved health is not a principal motivation for adolescents with obesity who attend a weight management intervention (21). Instead, the motivation to lose weight is more commonly related to a desire to be "normal" and socially desirable rather than preventing health consequences. Motivators for weight loss thus should resonate with the adolescent in the short-term; suggestions include increased clothing choices to attend social gatherings, reduced bullying, fewer unwelcome comments by family members, and, in more affluent contexts, material rewards for weight loss such as jewellery, games, or outings (37).

This review addresses dietary and physical exercise components often included in weight management programmes, however, in isolation these intervention components are unlikely to achieve their full impact. Adolescents often need emotional and psychological support within a weight management programme, alongside nutrition and physical activity (38). This is confirmed by at least one of the featured reviews included in this synthesis (21). Support must also extend longer term beyond the engagement phase. Longer-term weight loss interventions may decrease the risk of subsequent development of eating disorders (39) and are affirmed by qualitative data that reveals that young people value longer-term support once they have completed a weight loss intervention (21). Health services should consider whether more costly longer-term interventions may realise benefits in terms of maintenance of weight loss behaviours that mean that they don’t encounter ongoing repeat presentation from young person continuing onwards into adulthood.

Limitations of review

The main limitation of this review is the transferability of the findings. Most of the included studies were from high-income countries in the continents of Europe and North America with very few studies from Africa, South East Asia, South America or the Western Pacific. This is of great concern since lower-income countries, mainly in Africa and Asia are facing rapid increases in obesity prevalence. Also, the reviews did not usually focus on differences between children and adolescents. Instead, they discussed age groups and parental attitudes in general. Only one review focused exclusively on the attitudes or experiences of children who are less than ten years old (19). Few studies looked at how the acceptability of certain types of physical activity may differ according to variations in religion, age, culture and ethnicity.

Although eligibility criteria required one qualitative study to be included in source reviews for inclusion in this overview of reviews, five reviews were identifiable as full qualitative evidence syntheses(17, 19, 24-26). These five reviews performed well for both methodological limitations (minor-to-moderate concerns) and richness of data (two rich and three moderate) and made a major contribution to the findings. While many findings from these five reviews were corroborated by reviews with methodological limitations and less richness, this review would have been strengthened by the availability of a greater number of formally conducted qualitative evidence syntheses.

A further limitation of this review of reviews is the decision to focus on “obesity management”. Wider public health initiatives taking place further ‘upstream’ are classified as “obesity prevention”. While some values and preferences relating to obesity management are shared with obesity prevention, only the former have been systematically harvested to populate this review. The focus of this review on the values and preferences of children, adolescents, parents and caregivers means we did not explore the views of other adults, such as health workers, schoolteachers or other education or social care workers, even though these are also important. Indeed, their role was referenced by adolescents and caregivers in the included reviews. In addition, the data did not allow examination of specific individual exercise regimes or diet plans nor differences in types of exercise/activity or diet composition but focussed only on the values and preferences regarding the programmes themselves.

Implications for practice

Some children and adolescents are highly motivated to participate in diet or physical activity to help with their obesity, but others do not see the value of such activities or are reluctant to be involved.

Practitioners seeking to encourage children and adolescents with obesity to engage and participate in physical activities should consider the following:

(i) Are the planned activities fun, social, non-competitive, and frequent?

(ii) Are the planned activities appropriate for the child or adolescent, in terms of their age, gender and what they are physically capable of doing?

(iii) Do the planned activities seek to harness the support of family and friends?

(iv) Are safe and appropriate physical spaces, equipment and facilities available and accessible both where they live and at school, so that they can pursue such activities?

Practitioners seeking to encourage children and adolescents with obesity to engage and participate in dietary management intervention should consider the following:

(v) Is the dietary management programme interactive, social and fun?

(vi) Does the programme provide structured and coherent advice on diet and nutrition as well as information on health outcomes?

(vii) Does the dietary programme provide recipes and meal plans that are appropriate to the child and adolescent in terms of age, ethnicity and culture?

(viii) Are the recommended diet options palatable and affordable?

(ix) Is continuous and ongoing long-term support available from health workers, parents and peers throughout the dietary management programme and post-intervention?

Implications for future research

Findings were derived from fourteen reviews drawing upon a global evidence base from across 227 qualitative and mixed method studies, although evidence mostly derived from high income countries.

More research from Low- and Middle-Income Countries (LMICs) is needed. Although some included reviews reported population characteristics such as gender, age, ethnicity, and socio-economic status, these factors were largely absent from the evidence and analysis of most existing reviews. Other factors such as disability were not considered. The provision of such research data would facilitate granular analysis and nuanced understanding of factors that might apply to particular groups of young people.

Conclusion

This review identified multifaceted factors that influenced how young people with obesity and their caregivers engaged with physical activity (including exercise) and/or dietary management programmes. Interventions are unlikely to achieve any potential impact in isolation and young people often need emotional and psychological support within such programmes, alongside diet and physical activity. Our findings indicate that to encourage children, adolescents, parents, and caregivers to engage with weight management programmes, whole family involvement should be sought, and health workers should create a trusting relationship offering structured and coherent advice, that is appropriate and personalised in terms of both content and quantity. Furthermore, initiation and continued engagement with weight management programmes require ongoing support from family, health workers and peers in the form of anticipatory guidance and motivation. However, it should be noted that the evidence came predominantly from high-income countries and therefore potentially has limited generalisability.

Declarations

Acknowledgements: When preparing this review, we used EPOC's Protocol and Review Template for Qualitative Evidence Synthesis (Glenton C, Bohren MA, Downe S, Paulsen EJ, Lewin S, on behalf of Effective Practice and Organisation of Care (EPOC). EPOC Qualitative Evidence Synthesis: Protocol and review template. Version 1.1. EPOC Resources for review authors. Oslo: Norwegian

Institute of Public Health; 2020. Available at: http://epoc.cochrane.org/epoc-specific-resources-review-authors)

Availability of data and materials: The datasets used and/or analysed during the current study are available from the corresponding author on reasonable request

Competing interests: The authors declare that they have no competing interests
AB, CC, DC, JL, ME declared no financial conflicts of interest. AB, CC, JL, ME conducted work under an exclusive contract with the World Health Organization. DC worked on the reviews under a Sheffield Centre for Health and Related Research (SCHARR), University of Sheffield internship. Non-financial issues, including personal, political and academic factors, could have influenced the review authors' input when conducting this review.

Funding: The Department of Nutrition and Food Safety at the World Health Organization (WHO) commissioned and provided financial support to the University of Sheffield for this work. WHO acknowledges the financial support from the Norwegian Agency for Development Cooperation (NORAD), The Swedish International Development Cooperation Agency (SIDA), The Government of the Grand Duchy of Luxembourg, and the Government of Germany (BMG) to the Department of Nutrition and Food Safety.

References

1. Lobstein TJ-L, R.; Powis, J.; Brinsden, H.; and Gray, M. World Obesity Federation, World Obesity Atlas 2023 2023 [Available from: https://data.worldobesity.org/publications/?cat=19.

2. Okunogbe A, Nugent R, Spencer G, Powis J, Ralston J, Wilding J. Economic impacts of overweight and obesity: current and future estimates for 161 countries. BMJ global health. 2022;7(9):e009773.

3. (NCD-RisC). NRFC. Worldwide trends in body-mass index, underweight, overweight, and obesity from 1975 to 2016: a pooled analysis of 2416 population-based measurement studies in 128·9 million children, adolescents, and adults. Lancet. 2017;390:2627-42.

4. World Health Organization (WHO), United Nations Children's Fund (UNICEF), Bank. W. Levels and trends in child malnutrition: UNICEF / WHO / The World Bank Group joint child malnutrition estimates: key findings of the 2021 edition. World Health Organization. 2021.

5. Fruh SM. Obesity: Risk factors, complications, and strategies for sustainable long‐term weight management. Journal of the American association of nurse practitioners. 2017;29(S1):S3-S14.

6. United N, Affairs. DoEaS. Transforming our world: the 2030 Agenda for Sustainable Development https://sdgs.un.org/2030agenda (Accessed 01/09/2021). 2016.

7. Jackson Leach R, Powis J, Baur LA, Caterson ID, Dietz W, Logue J, et al. Clinical care for obesity: A preliminary survey of sixty‐eight countries. Clinical obesity. 2020;10(2):e12357.

8. Glenton C, Lewin S, Downe S, Paulsen E, Munabi-Babigumira S, Johansen M, et al. Qualitative evidence syntheses within Cochrane Effective Practice and Organisation of Care: Developing a template and guidance. International Journal of Qualitative Methods. 2021;20:16094069211041959.

9. Booth A, Noyes J, Flemming K, Moore G, Tunçalp Ö, Shakibazadeh E. Formulating questions to explore complex interventions within qualitative evidence synthesis. BMJ Glob Health. 2019;4(Suppl 1):e001107.

10. Organization WH. Health for the World's Adolescents: A Second Chance in the Second Decade. World Health Organization.; 2014.

11. Carroll C, Sworn K, A B, H P-H. What matters to children, parents or caregivers in child and adolescent obesity management: complementary syntheses (Mega-aggregation & Mega-ethnography) of qualitative systematic reviews. . Sheffield: School of Health and Related Research (ScHARR), University of Sheffield; 2021.

12. Booth A, Harris J, Croot E, Springett J, Campbell F, Wilkins E. Towards a methodology for cluster searching to provide conceptual and contextual "richness" for systematic reviews of complex interventions: case study (CLUSTER). BMC Med Res Methodol. 2013;13:118.

13. SBU:. Tool to assess methodological limitations of qualitative evidence synthesis. In. Stockholm, Sweden; 2019.

14. Carroll C, Sworn K, Booth A, Pardo‐Hernandez H. Stakeholder views of services for children and adolescents with obesity: Mega‐ethnography of qualitative syntheses. Obesity. 2022;30(11):2167-84.

15. Ames H, Glenton C, Lewin S. Purposive sampling in a qualitative evidence synthesis: a worked example from a synthesis on parental perceptions of vaccination communication. BMC Med Res Methodol. 2019;19(1):26.

16. Toye F, Seers K, Hannink E, Barker K. A mega-ethnography of eleven qualitative evidence syntheses exploring the experience of living with chronic non-malignant pain. BMC Med Res Methodol. 2017;17(1):116.

17. Stankov I, Olds T, Cargo M. Overweight and obese adolescents: what turns them off physical activity? Int. 2012;9:53.

18. Molina M, Anderson LN, Guindon GE, Tarride JE. A review of implementation and evaluation of Pan American Health Organization's policies to prevent childhood obesity in Latin America. Obes Sci Pract. 2021.

19. Burchett HED, Sutcliffe K, Melendez-Torres GJ, Rees R, Thomas J. Lifestyle weight management programmes for children: A systematic review using Qualitative Comparative Analysis to identify critical pathways to effectiveness. Prev Med. 2018;106:1-12.

20. Haracz K, Ryan S, Hazelton M, James C. Occupational therapy and obesity: An integrative literature review. Australian Occupational Therapy Journal. 2013;60(5):356-65.

21. Jones HM, Al-Khudairy L, Melendez-Torres GJ, Oyebode O. Viewpoints of adolescents with overweight and obesity attending lifestyle obesity treatment interventions: a qualitative systematic review. Obesity Reviews. 2019;20(1):156-69.

22. Kebbe M, Damanhoury S, Browne N, Dyson MP, McHugh T-LF, Ball GDC. Barriers to and enablers of healthy lifestyle behaviours in adolescents with obesity: a scoping review and stakeholder consultation. Obesity reviews : an official journal of the International Association for the Study of Obesity. 2017;18(12):1439-53.

23. Kelleher E, Davoren MP, Harrington JM, Shiely F, Perry IJ, McHugh SM. Barriers and facilitators to initial and continued attendance at community-based lifestyle programmes among families of overweight and obese children: a systematic review. Obesity Reviews. 2017;18(2):183-94.

24. Lachal J, Orri M, Speranza M, Falissard B, Lefevre H, Moro MR, et al. Qualitative studies among obese children and adolescents: A systematic review of the literature. Obesity Reviews. 2013;14(5):351-68.

25. Lang S, Gibson S, Ng KW, Truby H. Understanding children and young people's experiences pursuing weight loss maintenance using the Socio-ecological Model: A qualitative systematic literature review. Obesity Reviews. 2021;22(5):15.

26. Liu KSN, Chen JY, Ng MYC, Yeung MHY, Bedford LE, Lam CLK. How Does the Family Influence Adolescent Eating Habits in Terms of Knowledge, Attitudes and Practices? A Global Systematic Review of Qualitative Studies. Nutrients. 2021;13(11).

27. Roberts KJ, Binns HJ, Vincent C, Koenig MD. A Scoping Review: Family and Child Perspectives of Clinic-Based Obesity Treatment. Journal of Pediatric Nursing. 2021;57:56-72.

28. Zarnowiecki D, Mauch CE, Middleton G, Matwiejczyk L, Watson WL, Dibbs J, et al. A systematic evaluation of digital nutrition promotion websites and apps for supporting parents to influence children's nutrition. International Journal of Behavioral Nutrition and Physical Activity. 2020;17(1).

29. Chen J, Bai Y, Ni W. Reasons and promotion strategies of physical activity constraints in obese/overweight children and adolescents. Sports medicine and health science. 2024;6(1):25-36.

30. Skogen IB, Båtevik FO, Krumsvik RJ, Høydal KL. Weight-based victimization and physical activity among adolescents with overweight or obesity: a scoping review of quantitative and qualitative evidence. Frontiers in sports and active living. 2022;4:732737.

31. Page MJ, McKenzie JE, Bossuyt PM, Boutron I, Hoffmann TC, Mulrow CD, et al. The PRISMA 2020 statement: an updated guideline for reporting systematic reviews. International journal of surgery. 2021;88:105906.

32. Ball GD, Sebastianski M, Wijesundera J, Keto‐Lambert D, Ho J, Zenlea I, et al. Strategies to reduce attrition in managing paediatric obesity: A systematic review. Pediatric obesity. 2021;16(4):e12733.

33. Cislak A, Safron M, Pratt M, Gaspar T, Luszczynska A. Family‐related predictors of body weight and weight‐related behaviours among children and adolescents: A systematic umbrella review. Child: care, health and development. 2012;38(3):321-31.

34. Sung‐Chan P, Sung Y, Zhao X, Brownson R. Family‐based models for childhood‐obesity intervention: a systematic review of randomized controlled trials. Obesity Reviews. 2013;14(4):265-78.

35. Watson PM, Dugdill L, Pickering K, Hargreaves J, Staniford LJ, Owen S, et al. Distinguishing factors that influence attendance and behaviour change in family‐based treatment of childhood obesity: A qualitative study. British Journal of Health Psychology. 2021;26(1):67-89.

36. Shepherd J, Harden A, Rees R, Brunton G, Garcia J, Oliver S, et al. Young people and healthy eating: a systematic review of research on barriers and facilitators. Health education research. 2006;21(2):239-57.

37. Gow ML, Pham-Short A, Jebeile H, Varley BJ, Craig ME. Current perspectives on the role of very-low-energy diets in the treatment of obesity and type 2 diabetes in youth. Diabetes, Metabolic Syndrome and Obesity. 2021:215-25.

38. Jones HM, Oyebode O, Melendez-Torres G, Al-Khudairy L. Professional stakeholder's views of adolescent weight management programmes: a qualitative study. BMC Research Notes. 2021;14:1-6.

39. Jebeile H, Gow ML, Baur LA, Garnett SP, Paxton SJ, Lister NB. Treatment of obesity, with a dietary component, and eating disorder risk in children and adolescents: a systematic review with meta‐analysis. Obesity Reviews. 2019;20(9):1287-98.

Figure 1

Click here to access/download;Figure;Figure 1.tif

Figure 1:

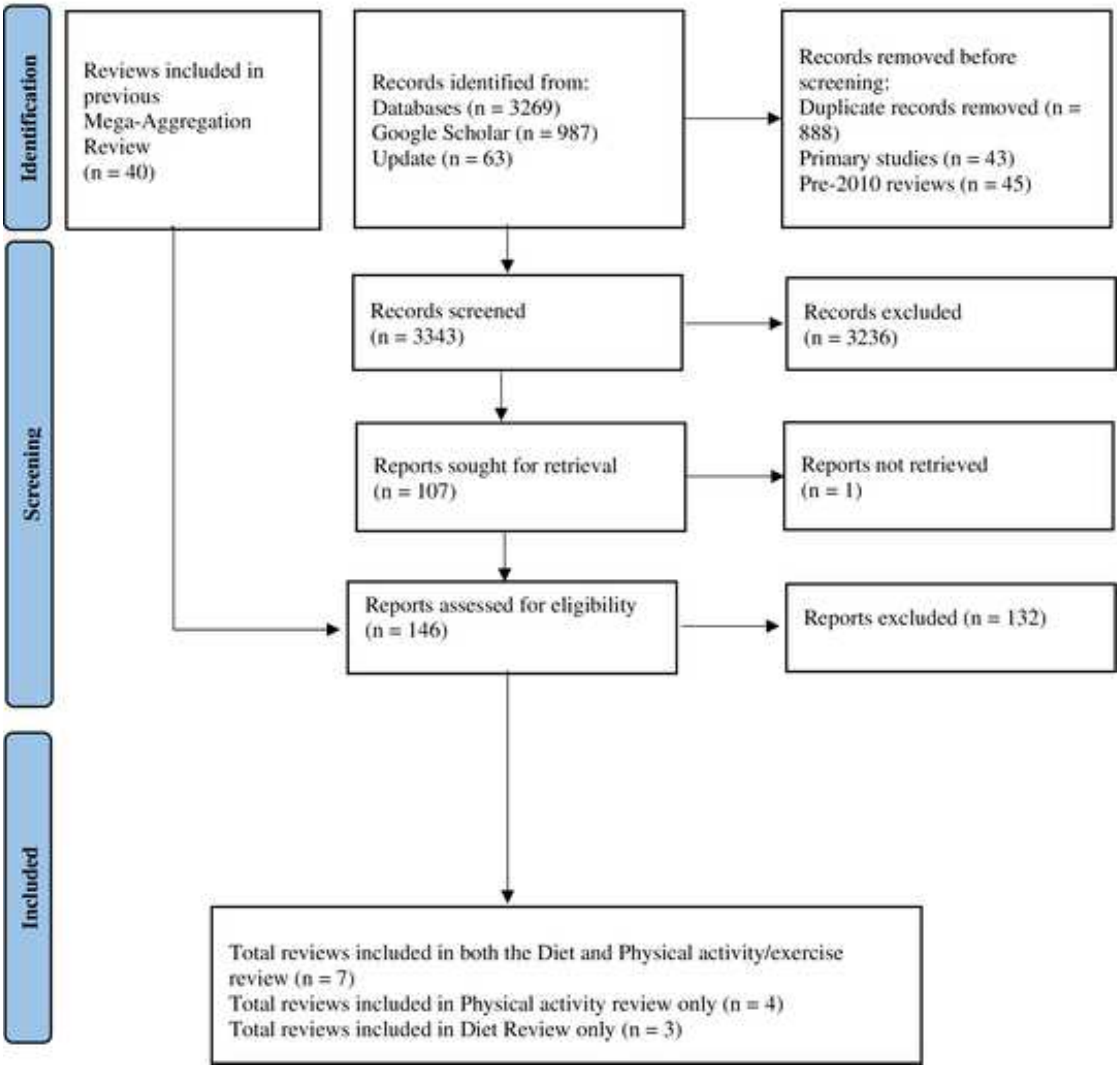

Figure 2

Click here to access/download;Figure;Figure 2.tif

Figure 2.

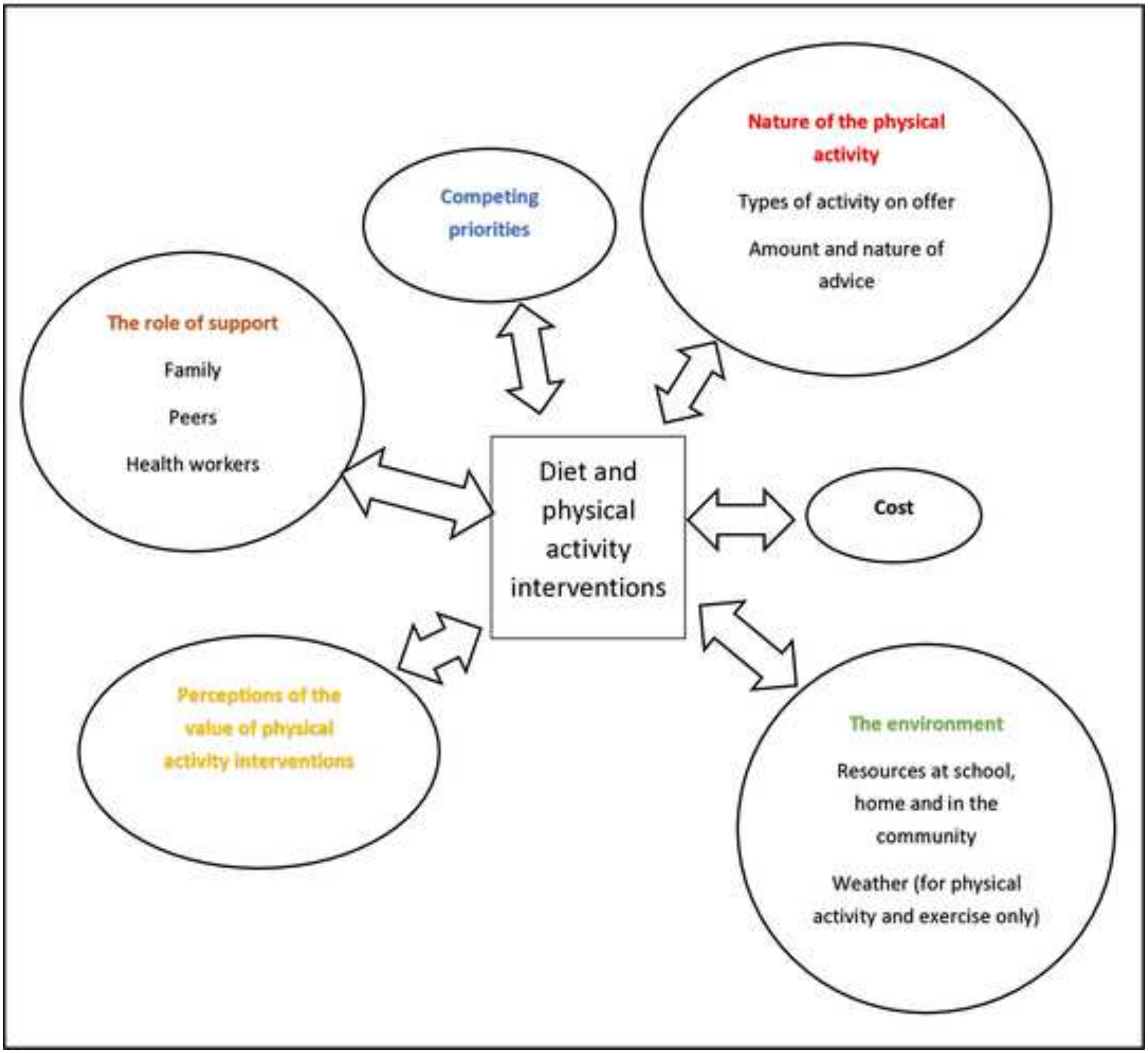

Click here to access/download
Supporting Information
PRISMA_2020_checklist Essat et al.docx

Click here to access/download
Supporting Information
Essat et al Supp Information.docx
