# Supplementary material for: Exploring the values and preferences of children and adolescents with obesity and their parents/caregivers concerning diet or physical activity interventions for weight management: Mega-ethnography of qualitative syntheses
Source: PLoS One. 2026 Jan 20;21(1):e0340875. doi: 10.1371/journal.pone.0340875 (PMC12818672; doi:10.1371/journal.pone.0340875)
Supplement: Attachment — Submitted filename: RG.Observations of the research artcle on Obesity.pdf [file pone.0340875.s015.pdf]

REVIEWER'S COMMENT

PLOS ONE Re: Exploring the values and preferences of children and adolescents with obesity and their parents/caregivers concerning diet or physical activity interventions for weight management: Mega-ethnography of qualitative syntheses of systematic reviews

Very Noble topic & Concept for Review

The manuscript explores the values and preferences of children and adolescents with obesity, as well as their parents/caregivers, regarding diet and physical activity interventions for weight management. It synthesizes findings from multiple qualitative systematic reviews using a mega-ethnography approach.

Observations

- ✓ The title of the study may be reconsidered. The title may be added Systemic Review, add problems and Remedies in the title. Also the age bracket like 5-18 years rather the generalized " Children and Adolescents"

- ✓ The manuscript acknowledges the predominance of studies from high-income countries. By including more studies from low- and middle-income countries would improve the generalizability of the findings. India and China may have the maximum children and adolescents with obesity. However, the review papers that were included in the analysis have minimal representation from these countries.

- ✓ While the manuscript discusses age and gender differences, a more detailed analysis of how these factors specifically influence engagement with interventions would be valuable.

- ✓ The manuscript briefly mentions ethical considerations but could provide a more detailed discussion on the ethical implications of the research, particularly regarding the involvement of children and adolescents.

- ✓ The discussion section remains silent on the following
  -The Lancet commission on obesity published its recommendations earlier this year (Definition and diagnostic criteria of clinical obesity - The Lancet Diabetes & Endocrinology). In light of these findings, the manuscript doesn't seem contemporary.

- ✓ -In light of widespread, at times off-label use of anti-obesity/chronic weight management therapies like semaglutide and tirzepatide in adolescents, relevance of non-pharmacological interventions need to be relooked.
