# Supplementary material for: Exploring the values and preferences of children and adolescents with obesity and their parents/caregivers concerning diet or physical activity interventions for weight management: Mega-ethnography of qualitative syntheses
Source: PLoS One. 2026 Jan 20;21(1):e0340875. doi: 10.1371/journal.pone.0340875 (PMC12818672; doi:10.1371/journal.pone.0340875)
Supplement: Attachment — Submitted filename: Response to Reviewers Essat et al.docx [file pone.0340875.s017.docx]

Dear Editor and Reviewers,

Thank you for reviewing our manuscript, we welcomed your constructive criticism and have improved the manuscript accordingly.

Below are responses to each comment raised.

|  | Editor’s Comments | Author’s Response |
| --- | --- | --- |
| 1. | Please ensure that your manuscript meets PLOS ONE's style requirements, including those for file naming. The PLOS ONE style templates can be found at  <https://journals.plos.org/plosone/s/file?id=wjVg/PLOSOne_formatting_sample_main_body.pdf> and  [https://journals.plos.org/plosone/s/file?id=ba62/PLOSOne_formatting_sample_title_authors_affiliations.pdf](https://track.editorialmanager.com/CL0/https:%2F%2Fjournals.plos.org%2Fplosone%2Fs%2Ffile%3Fid=ba62%2FPLOSOne_formatting_sample_title_authors_affiliations.pdf/1/010f01969f085fcd-0796c619-8583-466d-962c-1208b3739393-000000/CIaqZFdBdWW_7FnoC59TGvgCRpUuOK-TO_PGwb67Kek=209). | The manuscript has been updated to reflect PLOS ONE’S style requirements |
| 2. | Thank you for stating in your Funding Statement:  “The Department of Nutrition and Food Safety at the World Health Organization (WHO) commissioned and provided financial support to the University of Sheffield for this work. WHO acknowledges the financial support from the Norwegian Agency for Development Cooperation (NORAD), The Swedish International Development Cooperation Agency (SIDA), The Government of the Grand Duchy of Luxembourg, and the Government of Germany (BMG) to the Department of Nutrition and Food Safety.”  Please provide an amended statement that declares *all* the funding or sources of support (whether external or internal to your organization) received during this study, as detailed online in our guide for authors at <http://journals.plos.org/plosone/s/submit-now>.  Please also include the statement “There was no additional external funding received for this study.” in your updated Funding Statement.  Please include your amended Funding Statement within your cover letter. We will change the online submission form on your behalf. | The Funding statement has been updated as below and included in the cover letter:  The Department of Nutrition and Food Safety at the World Health Organization (WHO) commissioned and provided financial support to the University of Sheffield for this work. WHO acknowledges the financial support from the Norwegian Agency for Development Cooperation (NORAD), The Swedish International Development Cooperation Agency (SIDA), The Government of the Grand Duchy of Luxembourg, and the Government of Germany (BMG) to the Department of Nutrition and Food Safety. There was no additional external funding received for this study. |
| 3. | We note that your Data Availability Statement is currently as follows: [All relevant data are within the manuscript and its Supporting Information files.]  Please confirm at this time whether or not your submission contains all raw data required to replicate the results of your study. Authors must share the “minimal data set” for their submission. PLOS defines the minimal data set to consist of the data required to replicate all study findings reported in the article, as well as related metadata and methods (<https://journals.plos.org/plosone/s/data-availability#loc-minimal-data-set-definition>).  For example, authors should submit the following data:  - The values behind the means, standard deviations and other measures reported;  - The values used to build graphs;  - The points extracted from images for analysis.  Authors do not need to submit their entire data set if only a portion of the data was used in the reported study.  If your submission does not contain these data, please either upload them as Supporting Information files or deposit them to a stable, public repository and provide us with the relevant URLs, DOIs, or accession numbers. For a list of recommended repositories, please see <https://journals.plos.org/plosone/s/recommended-repositories>.  If there are ethical or legal restrictions on sharing a de-identified data set, please explain them in detail (e.g., data contain potentially sensitive information, data are owned by a third-party organization, etc.) and who has imposed them (e.g., an ethics committee). Please also provide contact information for a data access committee, ethics committee, or other institutional body to which data requests may be sent. If data are owned by a third party, please indicate how others may request data access. | We confirm our submission contains all raw data required to replicate the results of our review. The data is available either in the main manuscript or in the supplementary file (see response to point 5). As this is an overview of reviews – a secondary analysis, all data used are secondary data which are publicly available. The search strategy used to identify the studies are provide in the supplementary file. In addition we have added an ethical statement to the methods section of the manuscript (Page 5, line 115). |
| 4. | Thank you for stating the following in the Acknowledgments Section of your manuscript:  “The Department of Nutrition and Food Safety at the World Health Organization (WHO) commissioned and provided financial support to the University of Sheffield for this work. WHO acknowledges the financial support from the Norwegian Agency for Development Cooperation (NORAD), The Swedish International Development Cooperation Agency (SIDA), The Government of the Grand Duchy of Luxembourg, and the Government of Germany (BMG) to the Department of Nutrition and Food Safety. “  We note that you have provided additional information within the Acknowledgements Section that is not currently declared in your Funding Statement. Please note that funding information should not appear in the Acknowledgments section or other areas of your manuscript. We will only publish funding information present in the Funding Statement section of the online submission form.  Please remove any funding-related text from the manuscript and let us know how you would like to update your Funding Statement. Currently, your Funding Statement reads as follows:  “The Department of Nutrition and Food Safety at the World Health Organization (WHO) commissioned and provided financial support to the University of Sheffield for this work. WHO acknowledges the financial support from the Norwegian Agency for Development Cooperation (NORAD), The Swedish International Development Cooperation Agency (SIDA), The Government of the Grand Duchy of Luxembourg, and the Government of Germany (BMG) to the Department of Nutrition and Food Safety.  “  Please include your amended statements within your cover letter; we will change the online submission form on your behalf. | Thank you for highlighting this.  The Funding Statement has been removed from the Acknowledgement Section. An updated funding statement has been included in the cover letter. See point 2. |
| 5. | As required by our policy on Data Availability, please ensure your manuscript or supplementary information includes the following:  (i) A numbered table of all studies identified in the literature search, including those that were excluded from the analyses.  For every excluded study, the table should list the reason(s) for exclusion.  If any of the included studies are unpublished, include a link (URL) to the primary source or detailed information about how the content can be accessed.  (ii) A table of all data extracted from the primary research sources for the systematic review and/or meta-analysis. The table must include the following information for each study:  Name of data extractors and date of data extraction  Confirmation that the study was eligible to be included in the review.  All data extracted from each study for the reported systematic review and/or meta-analysis that would be needed to replicate your analyses.  If data or supporting information were obtained from another source (e.g. correspondence with the author of the original research article), please provide the source of data and dates on which the data/information were obtained by your research group.  (iii) If applicable for your analysis, a table showing the completed risk of bias and quality/certainty assessments for each study or outcome.  Please ensure this is provided for each domain or parameter assessed. For example, if you used the Cochrane risk-of-bias tool for randomized trials, provide answers to each of the signalling questions for each study. If you used GRADE to assess certainty of evidence, provide judgements about each of the quality of evidence factor. This should be provided for each outcome.  An explanation of how missing data were handled.  This information can be included in the main text, supplementary information, or relevant data repository. Please note that providing these underlying data is a requirement for publication in this journal, and if these data are not provided your manuscript might be rejected | (i) Thank you for your comment. In accordance with the journal’s Data Availability policy and the PRISMA 2020 guidelines we included a supplementary table (Supplementary Table S1) listing all studies that were excluded at the full-text screening stage, along with the specific reason(s) for exclusion. This is also consistent with guidance provided in the Cochrane Handbook for Systematic Reviews of Interventions which recommends transparent reporting of study selection decisions. In addition a PRISMA flow diagram has been included as Fig 1, documenting the number of studies identified by the searches and the final number of reviews included.  We confirm that all included studies are published and accessible via the provided references. No unpublished studies were included in the review  (ii) A table of all data extracted from the primary research sources has been captured in the main manuscript (Page 12, Table 3 and Page 13, Table 4) and additional data been added to Supplementary file, S2 and S3 Tables.  (iii) A completed quality assessment for each review has been presented in the main manuscript (Page 17, Table 5) and details of signalling questions and assessment are provided in Supplementary file S4 Table. |
|  |  |  |
|  | Reviewer’s comments | Authors’ response |
|  | Reviewer #1: |  |
| 6. | A very Noble topic for Review | Thank you |
| 7. | The title of the study may be reconsidered. The title may be added Systemic Review, add problems and Remedies in the title. Also the age bracket like 5-18 years rather the generalized “ Children and Adolescents” | Current title: Exploring the values and preferences of children and adolescents with obesity and their parents/caregivers concerning diet or physical activity interventions for weight management: Mega-ethnography of qualitative syntheses of systematic reviews  Thank you for your suggestion. After careful consideration, we believe that the current title reflects the scope and focus of the study as per our PROSPERO protocol.  We have intentionally used the broader terms “children and adolescents” to encompass the full age range by the included studies, rather than specifying a narrower age bracket. Additionally, the phrase “mega-ethnography of qualitative syntheses” precisely describes the methodology and nature of the review, which we feel is important to convey clearly in the title. As requested Systematic Review has been removed. |
| 8. | The manuscript acknowledges the predominance of studies from high-income countries. By including more studies from low- and middle-income countries would improve the generalizability of the findings. India and China may have the maximum children and adolescents with obesity. However, the review papers that were included in the analysis have minimal representation from these countries. | Thank you for your comment. We agree that including more studies from low- and middle-income countries would improve the generalisability of the findings. As noted in the Limitations Section of our Discussion, we identified very few eligible reviews including studies from these regions for inclusion in our analysis. This highlights an important gap in the existing literature and underscores the need for further research in low- and middle-income countries, including those with large populations of children and adolescents affected by obesity, such as India and China. The discussion section has been edited to reflect this (see Page 35, line 644) |
| 9. | While the manuscript discusses age and gender differences, a more detailed analysis of how these factors specifically influence engagement with interventions would be valuable. | Thank you for your valuable comment. We have expanded our Discussion to address this point in more detail “… while many reviews grouped children and adolescents together, limited age-specific analysis restricts understanding of how engagement with obesity management programmes might differ across developmental stages. Only one review focused exclusively on children under ten years old, which raises concerns about whether the perspectives and needs of younger children have been sufficiently captured. This could lead to an underrepresentation of age-specific barriers and facilitators in the findings.” (see Page 35, line 655). |
| 10. | The manuscript briefly mentions ethical considerations but could provide a more detailed discussion on the ethical implications of the research, particularly regarding the involvement of children and adolescents | Thank you for your comment. We have reviewed the manuscript and note that ethical considerations were not explicitly discussed in the manuscript. As this study is a systematic review synthesizing existing published studies, it does not involve primary data collection or direct participant involvement. Therefore, traditional ethical considerations such as informed consent etc, are not applicable. However, we acknowledge the importance of ethical issues involved with speaking sensitively to children and adolescents about their weight. The following has been added to the Methods section.  “This review did not require separate ethical approval, as it involved secondary analysis of anonymised data from previously published studies. Accordingly, all included primary studies had obtained appropriate ethical approval.” (Page 5, line 115) |
| 11 | The discussion section remains silent on the following -The Lancet commission on obesity published its recommendations earlier this year (Definition and diagnostic criteria of clinical obesity - The Lancet Diabetes & Endocrinology). In light of these findings, the manuscript doesn’t seem contemporary  -In light of widespread, at times off-label use of anti-obesity/chronic weight management therapies like semaglutide and tirzepatide in adolescents, relevance of non-pharmacological interventions need to be relooked | Thank you for your detailed and thoughtful feedback. Although pharmacological therapies fall outside the scope of our current review, we appreciate the importance of the recent Lancet commission’s recommendations on the definition and diagnostic criteria of clinical obesity, as well as the evolving landscape of obesity management, including the increasing use of pharmacological therapies. As such we have added the following to our Discussion Section:  “In addition, whilst dietary and physical activity interventions have traditionally formed the cornerstone of obesity management, the increasing adoption of pharmacological therapies, particularly GLP-1 receptor agonists warrants scrutiny of the evolving evidence base,[40] especially in adolescent populations. It is essential to assess the short-term acceptability of these agents to patients and to examine how this acceptability may influence longer-term outcomes. This includes monitoring for adverse events and evaluating the potential for relapse into unhealthy dietary and physical activity patterns following discontinuation of pharmacotherapy treatment.” (Page 38, line 730) |
|  |  |  |
|  | Reviewer #2: |  |
| 12. | The research appears ethically sound, with no overt ethical issues in participant recruitment or data handling. | Thank you |
| 13. | The objectives and background are well motivated and framed within the context of public health needs. | Thank you |
| 14. | The methodology section lacks sufficient detail to ensure full reproducibility. For example, information about the data collection process, survey tools, or any instruments used is limited. | Thank you for your comment. We appreciate your emphasis on the importance of methodological transparency and reproducibility. We believe that we have provided the necessary information regarding the data collection process and instruments used. Specifically, this information is detailed in the Methods Section on Pages 5 to 9, including eligibility criteria, methods used for study selection, the type of data collected, and the quality assessment tool used to assess the methodological quality of the included reviews. However, we recognize that certain aspects may not have been as clearly presented as intended. In response to your helpful suggestion, we have revised the Methods Section to improve clarity and ensure that all relevant details are more explicitly described. |
| 15. | Statistical methods are not comprehensively described—clarify which tests were used, why, and include confidence intervals or effect sizes. | Thank you for your comment. We would like to clarify that this review employed mega-ethnography, which is a qualitative evidence synthesis method. As such, statistical analyses including hypothesis tests, effect sizes, or confidence intervals are not applicable in this context. |
| 16. | There is no clear data availability statement, which is a core requirement of PLOS ONE. | See response to editor’s comments 3 and 5 |
| 17. | The manuscript does not mention whether datasets are publicly accessible or under restricted access. | See response to editor’s comments 3 and 5 |
| 18. | The structure follows a standard IMRAD format (Introduction, Methods, Results, and Discussion) | Thank you |
| 19. | The abstract provides a useful summary, though it could be tightened for clarity. | Thank you.  The abstract has been reviewed and revised for clarity. |
| 20. | There are grammatical issues and awkward phrasing in several sections (e.g., Introduction and Results). | Thank you for pointing this out. We have carefully reviewed the manuscript and revised sections of the Introduction, Results, and other relevant parts to improve grammar, clarity, and phrasing. |
| 21. | Transitions between paragraphs are sometimes abrupt, reducing narrative coherence. | See response to comment 14 and 20. |
| 22. | Conclusions are consistent with the reported findings. | Thank you |
| 23. | The discussion links findings back to the literature. | Thank you |
| 24. | Some conclusions may overstate the findings given the study’s limitations (e.g., generalizability or potential biases). | Thank you for your valuable feedback. We appreciate the importance of appropriately framing our conclusions in light of the study’s limitations. In response, we have revised the Conclusion Section to more clearly acknowledge the potential limitations related to generalizability and possible biases. (Page 38 -39) |
| 25. | Limitations are acknowledged but not deeply discussed. | Thank you for your insightful comment. We have expanded the Limitations section to provide a more in-depth discussion of the review’s potential weaknesses. |
| 26. | Tables summarize data well and are mostly easy to interpret. | Thank you |
| 27. | Tables lack clear titles and captions in some cases. | Thank you for your valuable feedback. We have amended this. |
| 28. | No figures or supplementary files were referenced | Thank you for your comment. We have carefully reviewed the manuscript and confirmed that all figures and supplementary files are referenced appropriately within the main text. If there are any specific sections where you feel references are missing, we would appreciate your guidance so we can address them accordingly |
