# Supplementary material for: Exploring the values and preferences of children and adolescents with obesity and their parents/caregivers concerning diet or physical activity interventions for weight management: Mega-ethnography of qualitative syntheses
Source: PLoS One. 2026 Jan 20;21(1):e0340875. doi: 10.1371/journal.pone.0340875 (PMC12818672; doi:10.1371/journal.pone.0340875)
Supplement: Attachment — Submitted filename: Response to Reviewers comments Essat et al R.docx [file pone.0340875.s018.docx]

Dear Editor and Reviewer,

Thank you for reviewing our manuscript. We welcomed your constructive criticism and have improved the manuscript accordingly.

Below are responses to each comment raised.

| PLoS ONE and Reviewer’s Comments | Author Response |
| --- | --- |
| PLoS ONE |  |
| 1. Please ensure that this letter is addressed specifically to PLoS ONE.  Please also include * why this manuscript is suitable for publication in PLoS ONE. * how does your paper provide a worthwhile addition to the scientific literature? * how does your paper relate to previously published work? * which types of scientists do you believe will be most interested in your study? | We have updated the cover letter as requested |
| 2. Please amend the title either on the online submission form or in your so that they are identical. | We have checked the tile and both the online submission title and the title in our manuscript are identical “Exploring the values and preferences of children and adolescents with obesity and their parents/caregivers concerning diet or physical activity interventions for weight management: Mega-ethnography of qualitative syntheses” |
| **Reviewer #3:** |  |
| I agree with earlier reviewers that this study addresses an important topic, and the results are valuable for informing the refinement and redesign of lifestyle interventions for children and adolescents with obesity. My suggestions and concerns are mainly related to the study framing and rationale, integration of the study aims and results into the broader literature, and discussion of how the results could be applied. | Thank you for your helpful suggestions. The manuscript has been updated accordingly. |
| 1. I would recommend that the introduction provide an argument for why it is important to understand the values and preferences of patients, and their respective ‘communities’ (families), in relation to interventions and treatment. This argument can draw on literature from community-engaged research and translational science principles, and the evidence base documenting how program/treatment adoption and efficacy can be improved by ensuring interventions align with patient and community priorities. I would also recommend this be highlighted in the discussion as motivation for why interventionists should use the results from this study. | Thank you for the helpful suggestion.  The Introduction and Discussion section has been updated. |
| 2. Line 98-99: This sentence is a little confusing: “This paper includes the findings from two of these reviews, examining the evidence on dietary and physical activity interventions”. What are the “two reviews”? Are they separate reviews on (i) diet interventions for children and adolescents, and (ii) physical activity interventions for children and adolescents? Please clarify. | For clarity, this has been amended to:  This paper reports the findings of the two systematic reviews that examine the evidence related to (i) dietary interventions for children and adolescents living with obesity, and (ii) physical activity interventions for children and adolescents living with obesity. |
| 3. Related to the item above, please clarify in the introduction, methods, and results if the goal of the review was to review interventions that targeted (i) diet only, OR (ii) physical activity only, OR (iii) diet and physical activity jointly (but not other health behaviors). | As suggested, for clarity text has been added to the introduction, methods and results that the goal of the review was to review interventions that targeted (i) diet only and/OR (ii) physical activity only. |
| 4. Line 100-101: Please clarify whose ‘values and preferences’ have been excluded (children, parents, both?), as well as clarify what these values and preference are of (e.g., values related to the treatment for obesity in children and adolescents)? | Thank you for your comment. The text has been amended as follows:  values and preferences of children, adolescents, and/ or their parents/caregivers, that influence engagement and adherence to dietary and physical activity interventions for obesity management in children and adolescents. |
| Methods  5. Table 1: There are several aspects of this table that I found unclear or confusing: (i) -Please clarify what “Setting” refers to. E.g., is the setting in which the intervention is delivered? (ii) -Please clarify why the focal ‘Setting’ for children is “Home” (but not childcare or school), and why the focal ‘Setting’ for adolescents is “School” (but not home). And why is the “Setting” for parents/caregivers only “Clinical settings”? (iii)-Under “Phenomenon of Interest”: use punctuation to make it clear there are two ‘phenomenon’ listed here (Diet intervention; Physical activity intervention) (iv) -I am not clear how to interpret the correspondence of the rows under the “Time/Timing” and “Findings columns: is Time = “Initiation” only applicable to the “Children with Obesity” row; and “Continuation” only applicable to the “Adolescents with obesity row”. I have the same confusion interpreting the rows under “Findings”. (v) -Line 24, replace “Plus” with “+” so it is clear you are referencing this symbol in “PROGRESS+” | Thank you for bringing this to our attention.   (i) Setting (Intervention delivery) (ii) This is a formatting error and has been resolved (iii) Punctuation has been added (iv) This is a formatting error and has been resolved (v) “Plus” has been replaced with “+” |
| 6. Line 137: Revise “for individuals and groups with obesity” to “for individuals with obesity, either delivered in an individual or group setting”. Similarly, could the diet interventions (lines 137-139) be delivered in individual or group formats? | This has been revised as suggested. |
| 7. Lines 142-143: I do not understand what this sentence means – can you revise for clarity? “Reviews were excluded if the health effects of the interventions could not be attributed distinctly to diet or physical activity or explored diet or physical activity for obesity prevention.” E.g., reviews were only included if the outcomes being measured (e.g., weight loss) were attributed to an intervention component focused on dietary change, or physical activity change, which presumably was a randomized controlled trial testing effects of specific components? It’s unclear how this applies to qualitative research. I also do not understand the point being made in the second part of the sentence, about prevention. | Thank you. We have amended the paragraph as follows:  The focus of this review was on qualitative data describing the values and preferences of children and adolescents, and of their parents/caregivers, regarding these types of interventions generally. Reviews focusing only on a specific type of exercise or physical activity, or composition of diet were excluded. Furthermore, reviews exploring diet or physical activity for prevention of obesity (i.e. in population who were not obese) were excluded. |
| 8. Data Extraction (lines 178-189). Could you provide more detail about the method for extracting themes of qualitative analysis results that were summarized in the reviews? E.g., were all themes or topics in the reviews transferred verbatim to a qualitative data management tool, or did any synthesis or transformation of the text about these themes occur at this data extraction stage? | Thank you for your suggestion. We have now included additional text to clarify the process. Furthermore, this process is, in part, depicted in the supplementary tables, where the third and related fourth-order constructs, and any supporting first order constructs (illustrative quotations) are provided. |
| 9. Line 195: Is QES an acronym that stands for something? If so, please define it here. | Thanks for highlighting this.  QES – has been written in full: Qualitative Evidence Syntheses |
| 10. Line 238: What does “0/6” years mean, when referring to a child’s age? | Thank you. This was a typo and has been amended |
| 11. Lines 241: In the sentence “one review [20] that drew upon evidence exclusively from that region” please state what region this is referring to. | Thank you – This has been amended.  one review [20] that drew upon evidence exclusively from Western Pacific region. |
| 12. Table 3: Under the column “Number of Qualitative studies” please define what the numbers reported represent (i.e., what is the first number, and what is the second bracketed number?). | Thank you - This has been added: (Number of total studies in the review) |
| 13. Table 5: Representing the 3 categories as colors (e.g., low = green, orange = medium, red = high) could provide an easier visual interpretation of the patterns of ‘risk to rigor’ across studies and across evaluation features. | Thank you for the helpful suggestion. This has now been done |
| 14. Table 6: I’d suggest revising the header “The role of support” as “The role of social support”, so it is clear this supporting is coming from interpersonal relationships (and to use this label throughout the manuscript). | This has been revised throughout the manuscript |
| 15. Figure 2: Why are the arrows between “interventions” and each of the elicited ‘factors that influence engagement in interventions’ bidirectional? The bidirectional arrows imply the factors influence the intervention, and that the intervention influences the factors – is that the intended meaning? | Thank you for highlighting this.  The original intention of the bidirectional arrows was to indicate potential dynamic interactions between interventions and influencing factors; however, we acknowledge that this may have introduced ambiguity regarding causal direction. To improve clarity and align with the primary focus of the figure i.e., identifying the factors that influence engagement in interventions. we have amended the figure so that the arrows point from "interventions" to the influencing factors only. |
| 16. Lines 383-396: With regards to the findings on health worker support, can you clarify in this section if the health workers being referred to are predominantly (or only) people who are delivering the intervention, or if the scope of the referent group for health workers is broader and might include health care providers that are not directly providing or delivering the intervention? | Thankyou – The text has been amended for clarity as follows:  The findings are referring to health workers delivering the session – This has been amended in text for clarity. |
| 17. Line 468: This sentence states “Parents preferred to enrol their children in programmes that focused on lifestyle..”. Please clarify what they preferred this over (i.e., they preferred enrolling their kids in programs focused on lifestyle changes, over what other options/what other programs that have a different focus)? | Thank you. For clarity the following text has been added:  “Parents preferred to enrol their children in programmes that focused on lifestyle (i.e., incorporated nutrition, physical activity and behavioural components) over programs with other primary focuses, such as those centered mainly on medication, clinical treatment, or short-term weight loss interventions.” |
| 18. Results: Why are quotes from parents and children so rarely included in the results, given they were included in the data extraction protocol? Their inclusion in lines 516-518 helps to integrate the patient/caregiver voice about their perspectives into the summary of the results. I would recommend including more of these quotes to illustrate key themes. | Quotes from parents and children have been added to the result section. |
| 19. Lines 528-529: The argument in the first few lines of this section is not clear. There are statements that children and caregivers prefer ‘knowledge’, ‘practical instruction’, and ‘evidence-based knowledge’, but they do not prefer ‘theory’. How does ‘evidence based knowledge’ differ to ‘theory’ when provided in the context of the interventions? | For clarity the sentence has been amended as follows:  Some children and adolescents with obesity, along with their caregivers, reported that interventions should provide them with new skills and knowledge. Some preferred practical, hands-on instruction over theory *“...you don’t want to hear theory when you’re a mum. You want to hear real-life experience and what’s practical for us”* , while others appreciated gaining evidence-based knowledge |
| 20. The discussion rarely integrates the study findings with the broader literature, apart from one paragraph (lines 593-613). I’d suggest that the first few paragraphs not just restate the findings, but interpret them in light of existing evidence and theory, and discuss how they could guide changes to intervention strategies. For example, in relation to the preferences for social support, refer to the extensive literature showing that family-based interventions are more effective than interventions that focus only on the child, and that social support is a known moderator of intervention outcomes; then, provide some examples of how interventions can be design to address this need (e.g., prioritize whole family interventions, include interventions with components to build social support among family and peers). The section “Implications for practice” provides questions that interventionists should ask themselves, but I think it would be useful to additionally provide examples of intervention strategies/protocols/components that actually address specific needs and preferences. E.g. child preferences for autonomy and choice could be addressed by the integration of motivational interviewing in the intervention delivery. | We thank the reviewer for this helpful suggestion. We have revised the Discussion to integrate the findings with existing literature and relevant theoretical frameworks (e.g., family-based intervention effectiveness, social support as a moderator, Self-Determination Theory).  We have also added examples of intervention strategies that align with the preferences and needs identified in the review (e.g., motivational interviewing, peer mentoring, family cooking sessions).  Additionally, the “Implications for Practice” section has been updated to include examples of programme components that address specific barriers and motivators. |
| 21. Lines 707-709: I think there is an opportunity here to highlight that pharmacological therapies, such as GLPs, can be effective for weight loss and reduced caloric intake, but may not be effective in addressing other health goals: e.g., increasing some healthy dietary behaviors (e.g., consumption of more nutrient dense foods or healthy eating habits, or increased physical activity). Arguably their delivery in adolescent populations should be complemented with lifestyle behavior change interventions, particularly to achieve sustainable improvements in child health outcomes. And so, the results of this study could be very valuable in informing those complimentary lifestyle intervention designs. | Thank you for the suggestion. The following has been added:  Furthermore, while pharmacological therapies such as GLP-1 receptor agonists can be effective in promoting weight loss and reducing caloric intake, they may be less effective in supporting other important health goals such as increasing the consumption of nutrient-dense foods, establishing healthy eating habits, or promoting regular physical activity. Delivery of pharmacological therapy in adolescent populations could be complemented with lifestyle behavior change interventions, particularly to achieve sustainable improvements in health outcomes. The findings of this review could be valuable in informing the design of such complementary lifestyle interventions. |
