# Supplementary material for: Exploring the values and preferences of children and adolescents with obesity and their parents/caregivers concerning diet or physical activity interventions for weight management: Mega-ethnography of qualitative syntheses
Source: PLoS One. 2026 Jan 20;21(1):e0340875. doi: 10.1371/journal.pone.0340875 (PMC12818672; doi:10.1371/journal.pone.0340875)
Supplement: Attachment — Submitted filename: Reponse to Reviewers Comments Essat el R2.docx [file pone.0340875.s019.docx]

Dear Editor and Reviewer,

Thank you for reviewing our manuscript. We welcomed your constructive criticism and have improved the manuscript accordingly.

Below are responses to each comment raised.

| **Reviewer’s Comments** | **Author’s Response** |
| --- | --- |
| **Reviewer #4:** |  |
| General observation: The search methods and eligibility criteria described is very nice and comprehensive. However, I could not find any guiding research questions in the introduction, objectives or purpose to help in finding relevant reviews and articles and guide the whole process of the research. | Thank you for this insightful observation. We agree that while our aim was stated, explicit research questions were missing, which are necessary to ground the search strategy and synthesis.  We have revised the final paragraph of the Introduction to explicitly list the two research questions that guided this review. These questions focus on identifying specific values and preferences, and understanding how they impact adherence and engagement. |
| 76 Is it possible to specify the geographical location? Whether it is globally or in America or Europe? Similarly, regarding the geographic scope of this review, I understand it includes reviews and studies across continents. Is this review aiming to inform WHO regarding the preferences and values of young people and their caregiver’s globally? Although it does mention it cannot be generalized for every setting as the studies included are mostly from high income countries in the limitation. Hence, is it possible to give a geographic scope to the review? | Thank you for the suggestion. We have made it clearer the specific geographical location and geographic scope of this review in both the introduction and method section. |
| 142 Comma (,) after the word inclusion | Comma added as requested |
| 143 Instead of “Mixed methods reviews were included”, which although correct is a little awkward, is it possible to change it to” Reviews that used mixed methods approach were included”. | Rephrased as suggested |
| 316 The sentence structure is a little awkward to read. Maybe rephrase “such as to prevent health sequelae “to “such as preventing health sequelae”. rephrase “to be” to “being” rephrase “to gain” to “gaining” | Rephrased as suggested |
| 351 Add the word “their” in front of the word “families” Add the word “other” between the words “many commitments” | This has been done as suggested |
| 354 Change the word “few” to a comparison word such as “fewer” or rephrase the sentence to a comparison sentence. | Rephrased to “fewer” |
| 382 The starting sentence is a little unclear. What do you mean by “such activities”? “The intervention activities such as physical exercises? Or diet management? | The sentence has been re-worded as suggested |
| 393 The information” This could create a sense of self-blaming.” Needs to be continued or explained as the following sentence does not explain it or connect with it. | Thank you for highlighting this – it is an error and has been removed. |
| 411 Delete the word “the” in front of the word “home” | “the” has been deleted as suggested |
| 512 “On offer” sounds more casual. Can we change it to “available” | The change has been done as suggested |
| 521 Is it possible to change “rued” to “complained” or another similar word. “Rued” sounds a little dramatic. | “Rued” has been changed to “complained” as suggested |
| 540 “On offer” if we can change it to another appropriate word | The word “on offer” has been changed to “available” |
| 578 The title “The amount and nature….” This section does not specify anything about the amount of advice given. In my opinion it is more about the quality or nature of advice on types of intervention. | This has been re-worded to “The quality and nature of advice on types of diet or physical activity interventions given to children and families is important” |
| 581 Delete the letter “e” between the words “provide” and “them, | Letter “e” has been deleted |
| 598 Maintain the consistency and add “adolescents “to the topic as well? | As suggested “adolescents” has been added to the topic |
